# Supplementary material for: What techniques might be used to harness placebo effects in non-malignant pain? A literature review and survey to develop a taxonomy
Source: BMJ Open. 2017 Jun 30;7(6):e015516. doi: 10.1136/bmjopen-2016-015516 (PMC5734496; doi:10.1136/bmjopen-2016-015516)
Supplement: Supplementary material 1 [file bmjopen-2016-015516supp001.pdf]

Supplementary Digital Content

List of Included Studies

[1-169]

[1] Alfano AP, Taylor AG, Foresman PA, Dunk PR, McConnell GG, Gillies GT. Static magnetic fields for treatment of fibromyalgia: A randomized controlled trial. . J Altern Complement Med 2001;7(1):53.

[2] Alford JW, Fadale PD. Evaluation of postoperative bupivacaine infusion for pain management after anterior cruciate ligament reconstruction. Arthroscopy-the Journal of Arthroscopic and Related Surgery 2003;19(8):855.

[3] Amanzio M, Benedetti F. Neuropharmacological Dissection of Placebo Analgesia: Expectation-Activated Opioid Systems versus Conditioning-Activated Specific Subsystems. The Journal of Neuroscience 1999;19(1):484-494.

[4] Amanzio M, Pollo A, Maggi G, Benedetti F. Response variability to analgesics: a role for non-specific activation of endogenous opioids. Pain 2001;90(3):205-215.

[5] Amlie E, Weber H, Holme I. Treatment of Acute Low-back Pain with Piroxicam: Results of a Double-blind Placebo-controlled Trial. Spine 1987;12(5):473-476.

[6] Andersen AN, Damm P, Tabor A, Pedersen IM, Harring M. Prevention of Breast Pain and Milk Secretion with Bromocriptine After Second-Trimester Abortion. Acta Obstet Gynecol Scand 1990;69(3):235-238.

[7] Aslaksen PM, Flaten MA. The Roles of Physiological and Subjective Stress in the Effectiveness of a Placebo on Experimentally Induced Pain. Psychosom Med 2008;70(7):811-818.

[8] Atkinson JH, Slater MA, Williams RA, Zisook S, Patterson TL, Grant I, Wahlgren DR, Abramson I, Garfin SR. A placebo-controlled randomized clinical trial of nortriptyline for chronic low back pain. Pain 1998;76(3):287-296.

[9] Bannwarth B, Allaert FA, Avouac B, Rossignol M, Rozenberg S, Valat JP. A randomized, double-blind, placebo controlled triphosphate in study of oral adenosine subacute low back pain. J Rheumatol 2005;32(6):1114-1117.

[10] Basford JR, Sheffield CG, Harmsen WS. Laser therapy: a randomized, controlled trial of the effects of low-intensity Nd:YAG laser irradiation on musculoskeletal back pain. Arch Phys Med Rehabil 1999;80(6):647-652.

[11] Basmajian JV. Acute back pain and spasm. A controlled multicenter trial of combined analgesic and antispasm agents. Spine (Phila Pa 1976) 1989;14(4):438-439.

[12] Benedetti F. The opposite effects of the opiate antagonist naloxone and the cholecystokinin antagonist proglumide on placebo analgesia. Pain 1996;64(3):535-543.

[13] Benedetti F, Amanzio M, Baldi S, Casadio C, Cavallo A, Mancuso M, Ruffini E, Oliaro A, Maggi G. The specific effects of prior opioid exposure on placebo analgesia and placebo respiratory depression. Pain 1998;75(2-3):313-319.

[14] Benedetti F, Amanzio M, Casadio C, Oliaro A, Maggi G. Blockade of nocebo hyperalgesia by the cholecystokinin antagonist proglumide. Pain 1997;71(2):135-140.

[15] Benedetti F, Amanzio M, Maggi G. Potentiation of placebo analgesia by proglumide. The Lancet 1995;346(8984):1231.

[16] Benedetti F, Amanzio M, Vighetti S, Asteggiano G. The Biochemical and Neuroendocrine Bases of the Hyperalgesic Nocebo Effect. The Journal of Neuroscience 2006;26(46):12014-12022.

[17] Benedetti F, Arduino C, Amanzio M. Somatotopic activation of opioid systems by target-directed expectations of analgesia. J Neurosci 1999;19(9):3639-3648.

[18] Benedetti F, Arduino C, Costa S, Vighetti S, Tarenzi L, Rainero I, Asteggiano G. Loss of expectation-related mechanisms in Alzheimer’s disease makes analgesic therapies less effective. Pain 2006;121(1):133-144.

[19] Benedetti F, Pollo A, Lopiano L, Lanotte M, Vighetti S, Rainero I. Conscious expectation and unconscious conditioning in analgesic, motor, and hormonal placebo/nocebo responses. The Journal of Neuroscience 2003;23(10):4315-4323.
[20] Benedetti FMD, Amanzio MMS, Casadio CMD, Cavallo AMD, Cianci RMD, Giobbe RMD, Mancuso MMD, Ruffini EMD, Maggi GMD. Control of Postoperative Pain by Transcutaneous Electrical Nerve Stimulation After Thoracic Operations. The Annals of Thoracic Surgery 1997;63(3):773-776.
[21] Berry H, Hutchinson DR. A multicentre placebo-controlled study in general practice to evaluate the efficacy and safety of tizanidine in acute low-back pain. J Int Med Res 1988;16(2):75-82.
[22] Bingel U, Lorenz J, Schoell E, Weiller C, Büchel C. Mechanisms of placebo analgesia: rACC recruitment of a subcortical antinociceptive network. Pain 2006;120(1):8-15.
[23] Birbara CA, Puopolo AD, Munoz DR, Sheldon EA, Mangione A, Bohidar NR, Geba GP. Treatment of chronic low back pain with etoricoxib, a new cyclo-oxygenase-2 selective inhibitor: improvement in pain and disability--a randomized, placebo-controlled, 3-month trial. The journal of pain : official journal of the American Pain Society 2003;4(6):307-315.
[24] Biro P, Meier T, Cummins AS. Comparison of topical anaesthesia methods for venous cannulation in adults. Eur J Pain 1997;1(1):37-42.
[25] Blanchard EB, Appelbaum KA, Radnitz CL, Michultka D, Morrill B, Kirsch C, Hillhouse J, Evans DD, Guarnieri P, Attanasio V, et al. Placebo-controlled evaluation of abbreviated progressive muscle relaxation and of relaxation combined with cognitive therapy in the treatment of tension headache. J Consult Clin Psychol 1990;58(2):210-215.
[26] Blanchard EB, Appelbaum KA, Radnitz CL, Morrill B, Michultka D, Kirsch C, Guarnieri P, Hillhouse J, Evans DD, Jaccard J, et al. A controlled evaluation of thermal biofeedback and thermal biofeedback combined with cognitive therapy in the treatment of vascular headache. J Consult Clin Psychol 1990;58(2):216-224.
[27] Boissel JP, Philippon AM, Gauthier E, Schbath J, Destors JM. Time course of long-term placebo therapy effects in angina pectoris. Eur Heart J 1986;7(12):1030-1036.
[28] Bova JG, Bhattacharjee N, Jurdi R, Bennett WF. Comparison of no medication, placebo, and hyoscyamine for reducing pain during a barium enema. Am J Roentgenol 1999;172(5):1285-1287.
[29] Brinkhaus B, Witt CM, Jena S, Linde K, Streng A, Wagenpfeil S, Irnich D, Walther HU, Melchart D, Willich SN. Acupuncture in patients with chronic low back pain - A randomized controlled trial. Arch Intern Med 2006;166(4):450-457.
[30] Camilleri M, Northcutt AR, Kong S, Dukes GE, McSorley D, Mangel AW. Efficacy and safety of alosetron in women with irritable bowel syndrome: a randomised, placebo-controlled trial. The Lancet 2000;355(9209):1035-1040.
[31] Carbajal R, Chauvet X, Couderc S, Olivier-Martin M. Randomised trial of analgesic effects of sucrose, glucose, and pacifiers in term neonates. BMJ 1999;319(7222):1393-1397.
[32] Carette S, Leclaire R, Marcoux S, Morin F, Blaise GA, St-Pierre A, Truchon R, Parent F, Levesque J, Bergeron V, Montminy P, Blanchette C. Epidural corticosteroid injections for sciatica due to herniated nucleus pulposus. N Engl J Med 1997;336(23):1634-1640.
[33] Charron J, Rainville P, Marchand S. Direct comparison of placebo effects on clinical and experimental pain. Clin J Pain 2006;22(2):204-211.
[34] Cherkin DC, Sherman KJ, Avins AL, et al. A randomized trial comparing acupuncture, simulated acupuncture, and usual care for chronic low back pain. Arch Intern Med 2009;169(9):858-866.
[35] Chrubasik S, Eisenberg E, Balan E, Weinberger T, Luzzati R, Conradt C. Treatment of low back pain exacerbations with willow bark extract: a randomized double-blind study. Am J Med 2000;109(1):9-14.

[36] Chrubasik S, Junck H, Breitschwerdt H, Conradt C, Zappe H. Effectiveness of Harpagophytum extract WS 1531 in the treatment of exacerbation of low back pain: a randomized, placebo-controlled, double-blind study. Eur J Anaesthesiol 1999;16(2):118-129.
[37] Chung SK, Price DD, Verne GN, Robinson ME. Revelation of a personal placebo response: Its effects on mood, attitudes and future placebo responding. Pain 2007;132(3):281-288.
[38] Classen W, Feingold E, Netter P. Influence of sensory suggestibility on treatment outcome in headache patients. Neuropsychobiology 1983;10(1):44-47.
[39] Coats TL, Borenstein DG, Nangia NK, Brown MT. Effects of valdecoxib in the treatment of chronic low back pain: results of a randomized, placebo-controlled trial. Clin Ther 2004;26(8):1249-1260.
[40] Colloca L, Benedetti F. How prior experience shapes placebo analgesia. Pain 2006;124(1-2):126-133.
[41] Colloca L, Benedetti F. Placebo analgesia induced by social observational learning. Pain 2009;144(1-2):28-34.
[42] Colloca L, Petrovic P, Wager TD, Ingvar M, Benedetti F. How the number of learning trials affects placebo and nocebo responses. Pain 2010;151(2):430-439.
[43] Colloca L, Sigaudo M, Benedetti F. The role of learning in nocebo and placebo effects. Pain 2008;136(1-2):211-218.
[44] Conn IG, Marshall AH, Yadav SN, Daly JC, Jaffer M. Transcutaneous electrical nerve stimulation following appendicectomy: the placebo effect. Ann R Coll Surg Engl 1986;68(4):191-192.
[45] Corson SL, Batzer FR, Gocial B, Kelly M, Gutmann JN, Go KJ, English ME. Is paracervical block anesthesia for oocyte retrieval effective? Fertil Steril 1994;62(1):133-136.
[46] Costello M, Ramundo M, Christopher NC, Powell KR. Ethyl Vinyl Chloride Vapocoolant Spray Fails to Decrease Pain Associated with Intravenous Cannulation in Children. Clin Pediatr (Phila) 2006;45(7):628-632.
[47] Coyne PJ, MacMurren M, Izzo T, Kramer T. Transcutaneous electrical nerve stimulator for procedural pain associated with intravenous needlesticks. J Intraven Nurs 1995;18(5):263-267.
[48] Dapas F, Hartman SF, Martinez L, Northrup BE, Nussdorf RT, Silberman HM, Gross H. Baclofen for the treatment of acute low-back syndrome. A double-blind comparison with placebo. Spine (Phila Pa 1976) 1985;10(4):345-349.
[49] De Pascalis V, Chiaradia C, Carotenuto E. The contribution of suggestibility and expectation to placebo analgesia phenomenon in an experimental setting. Pain 2002;96(3):393-402.
[50] deCharms RC, Maeda F, Glover GH, Ludlow D, Pauly JM, Soneji D, Gabrieli JDE, Mackey SC. Control over brain activation and pain learned by using real-time functional MRI. Proc Natl Acad Sci U S A 2005;102(51):18626-18631.
[51] Defrin R, Ariel E, Peretz C. Segmental noxious versus innocuous electrical stimulation for chronic pain relief and the effect of fading sensation during treatment. Pain 2005;115(1):152-160.
[52] Dickens C, Jayson M, Sutton C, Creed F. The relationship between pain and depression in a trial using paroxetine in sufferers of chronic low back pain. Psychosomatics 2000;41(6):490-499.
[53] Ditto B, France CR. The effects of applied tension on symptoms in French-speaking blood donors: a randomized trial. Health Psychol 2006;25(3):433-437.
[54] Ditto B, France CR, Lavoie P, Roussos M, Adler PS. Reducing reactions to blood donation with applied muscle tension: a randomized controlled trial. Transfusion (Paris) 2003;43(9):1269-1275.
[55] Dreiser RL, Marty M, Ionescu E, Gold M, Liu JH. Relief of acute low back pain with diclofenac-K 12.5 mg tablets: a flexible dose, ibuprofen 200 mg and placebo-controlled clinical trial. Int J Clin Pharmacol Ther 2003;41(9):375-385.
[56] Erdogmus CB, Resch KL, Sabitzer R, Muller H, Nuhr M, Schoggl A, Posch M, Osterode W, Ungersbock K, Ebenbichler GR. Physiotherapy-based rehabilitation following disc herniation

operation: results of a randomized clinical trial. Spine (Phila Pa 1976) 2007;32(19):2041-2049.
[57] Faas A, Chavannes AW, van Eijk JT, Gubbels JW. A randomized, placebo-controlled trial of exercise therapy in patients with acute low back pain. Spine (Phila Pa 1976) 1993;18(11):1388-1395.
[58] Fanti L, Gemma M, Passaretti S, Guslandi M, Testoni PA, Casati A, Torri G. Electroacupuncture Analgesia for Colonoscopy: A Prospective, Randomized, Placebo-Controlled Study. Am J Gastroenterol 2003;98(2):312-316.
[59] Forster EL, Kramer JF, Lucy SD, Scudds RA, Novick RJ. EFfect of tens on pain, medications, and pulmonary function following coronary artery bypass graft surgery. CHEST Journal 1994;106(5):1343-1348.
[60] Foster KA, Liskin J, Cen S, Abbott A, Armisen V, Globe D, Knox L, Mitchell M, Shtir C, Azen S. The Trager approach in the treatment of chronic headache: a pilot study. Altern Ther Health Med 2004;10(5):40-46.
[61] Foster NE, Thomas E, Barlas P, Hill JC, Young J, Mason E, Hay EM. Acupuncture as an adjunct to exercise based physiotherapy for osteoarthritis of the knee: randomised controlled trial. BMJ 2007;335(7617):436.
[62] Frega A, Stentella P, Di Renzi F, Gallo G, Palazzetti PL, Del Vescovo M, Ciccarone M, Pachi A. Pain evaluation during carbon dioxide laser vaporization for cervical intraepithelial neoplasia: a randomized trial. Clin Exp Obstet Gynecol 1994;21(3):188-191.
[63] Gale GD, Rothbart PJ, Li Y. Infrared therapy for chronic low back pain: a randomized, controlled trial. Pain Res Manag 2006;11(3):193-196.
[64] Geers A, Helfer S, Weiland P, Kosbab K. Expectations and Placebo Response: A Laboratory Investigation into the Role of Somatic Focus. J Behav Med 2006;29(2):171-178.
[65] Geers AL, Wellman JA, Fowler SL, Helfer SG, France CR. Dispositional optimism predicts placebo analgesia. J Pain 2010;11(11):1165-1171.
[66] Goffaux P, Redmond WJ, Rainville P, Marchand S. Descending analgesia--when the spine echoes what the brain expects. Pain 2007;130(1-2):137-143.
[67] Goodenough B, Kampel L, Champion GD, Laubreaux L, Nicholas MK, Ziegler JB, McInerney M. An investigation of the placebo effect and age-related factors in the report of needle pain from venipuncture in children. Pain 1997;72(3):383-391.
[68] Goodkin K, Gullion CM, Agras WS. A randomized, double-blind, placebo-controlled trial of trazodone hydrochloride in chronic low back pain syndrome. J Clin Psychopharmacol 1990;10(4):269-278.
[69] Grevert P, Albert LH, Goldstein A. Partial antagonism of placebo analgesia by naloxone. Pain 1983;16(2):129-143.
[70] Haake M, Muller HH, Schade-Brittinger C, Basler HD, Schafer H, Maier C, Endres HG, Trampisch HJ, Molsberger A. German acupuncture trials (GERAC) for chronic low back pain. Randomized, multicenter, blinded, parallel-group trial with 3 groups Arch Intern Med 2007;167(17):1892-1898.
[71] Hale ME, Ahdieh H, Ma T, Rauck R. Efficacy and safety of OPANA ER (oxymorphone extended release) for relief of moderate to severe chronic low back pain in opioid-experienced patients: a 12-week, randomized, double-blind, placebo-controlled study. The journal of pain : official journal of the American Pain Society 2007;8(2):175-184.
[72] Hargreaves A, Lander J. Use of Transcutaneous Electrical Nerve Stimulation For Postoperative Pain. Nurs Res 1989;38(3):159-160.
[73] Hargreaves KM, Dionne RA, Mueller GP. Plasma Beta-Endorphin-like Immunoreactivity, Pain and Anxiety Following Administration of Placebo in Oral Surgery Patients. J Dent Res 1983;62(11):1170-1173.
[74] Hashish I, Hai HK, Harvey W, Feinmann C, Harris M. Reduction of postoperative pain and swelling by ultrasound treatment: a placebo effect. Pain 1988;33(3):303-311.

[75] Hashish I, Harvey W, Harris M. Anti-inflammatory effects of ultrasound therapy: evidence for a major placebo effect. Rheumatology (Oxford) 1986;25(1):77-81.
[76] Helms JM. Acupuncture for the management of primary dysmenorrhea. Obstet Gynecol 1987;69(1):51-56.
[77] Hoirus KT, Pfleger B, McDuffie FC, Cotsonis G, Elsangak O, Hinson R, Verzosa GT. A randomized clinical trial comparing chiropractic adjustments to muscle relaxants for subacute low back pain. J Manipulative Physiol Ther 2004;27(6):388-398.
[78] Hong C-Z, Chen Y-C, Pon CH, Yu J. Immediate Effects of Various Physical Medicine Modalities on Pain Threshold of an Active Myofascial Trigger Point. Journal of Musculoskeletal Pain 1993;1(2):37-53.
[79] Hruby G, Ames C, Chen C, Yan Y, Sagar J, Baron P, Landman J. Assessment of efficacy of transcutaneous electrical nerve stimulation for pain management during office-based flexible cystoscopy. Urology 2006;67(5):914-917.
[80] Hyland MR, Webber-Gaffney A, Cohen L, Lichtman SW. Randomized Controlled Trial of Calcaneal Taping, Sham Taping, and Plantar Fascia Stretching for the Short-Term Management of Plantar Heel Pain. J Orthop Sports Phys Ther 2006;36(6):364-371.
[81] Johansen O, Brox J, Flaten MA. Placebo and Nocebo Responses, Cortisol, and Circulating Beta-Endorphin. Psychosom Med 2003;65(5):786-790.
[82] Kaptchuk TJ, Stason WB, Davis RB, Legedza ATR, Schnyer RN, Kerr CE, Stone DA, Nam BH, Kirsch I, Goldman RH. Sham device v inert pill: randomised controlled trial of two placebo treatments. Br Med J 2006;332(7538):391-394.
[83] Katz J, Pennella-Vaughan J, Hetzel RD, Kanazi GE, Dworkin RH. A Randomized, Placebo-Controlled Trial of Bupropion Sustained Release in Chronic Low Back Pain. J Pain 2005;6(10):656-661.
[84] Katz N, Ju WD, Krupa DA, Sperling RS, Bozalis Rodgers D, Gertz BJ, Gimbel J, Coleman S, Fisher C, Nabizadeh S, Borenstein D. Efficacy and safety of rofecoxib in patients with chronic low back pain: results from two 4-week, randomized, placebo-controlled, parallel-group, double-blind trials. Spine (Phila Pa 1976) 2003;28(9):851-858; discussion 859.
[85] Katz N, Rauck R, Ahdieh H, Ma T, Gerritsen van der Hoop R, Kerwin R, Podolsky G. A 12-week, randomized, placebo-controlled trial assessing the safety and efficacy of oxymorphone extended release for opioid-naive patients with chronic low back pain. Curr Med Res Opin 2007;23(1):117-128.
[86] Keltner JR, Furst A, Fan C, Redfern R, Inglis B, Fields HL. Isolating the Modulatory Effect of Expectation on Pain Transmission: A Functional Magnetic Resonance Imaging Study. The Journal of Neuroscience 2006;26(16):4437-4443.
[87] Kerr AR, Drexel CA, Spielman AI. The efficacy and safety of 50 mg penicillin G potassium troches for recurrent aphthous ulcers. Oral Surg Oral Med Oral Pathol Oral Radiol Endod 2003;96(6):685-694.
[88] Ketenci A, Ozcan E, Karamursel S. Assessment of efficacy and psychomotor performances of thiocolchicoside and tizanidine in patients with acute low back pain. Int J Clin Pract 2005;59(7):764-770.
[89] Klaber Moffett JA, Richardson PH, Frost H, Osborn A. A placebo controlled double blind trial to evaluate the effectiveness of pulsed short wave therapy for osteoarthritic hip and knee pain. Pain 1996;67(1):121-127.
[90] Klein RG, Eek BC. Low-energy laser treatment and exercise for chronic low back pain: double-blind controlled trial. Arch Phys Med Rehabil 1990;71(1):34-37.
[91] Kober A, Scheck T, Greher M, Lieba F, Fleischhackl R, Fleischhackl S, Randunsky F, Hoerauf K. Prehospital Analgesia with Acupressure in Victims of Minor Trauma: A Prospective, Randomized, Double-Blinded Trial. Anesth Analg 2002;95(3):723-727 710.1213/00000539-200209000-200200035.

[92] Kong J, Gollub RL, Polich G, Kirsch I, LaViolette P, Vangel M, Rosen B, Kaptchuk TJ. A Functional Magnetic Resonance Imaging Study on the Neural Mechanisms of Hyperalgesic Nocebo Effect. The Journal of Neuroscience 2008;28(49):13354-13362.
[93] Kong J, Gollub RL, Rosman IS, Webb JM, Vangel MG, Kirsch I, Kaptchuk TJ. Brain activity associated with expectancy-enhanced placebo analgesia as measured by functional magnetic resonance imaging. J Neurosci 2006;26(2):381-388.
[94] Kotani N, Kushikata T, Suzuki A, Hashimoto H, Muraoka M, Matsuki A. Insertion of intradermal needles into painful points provides analgesia for intractable abdominal scar pain. Reg Anesth Pain Med 2001;26(6):532-538.
[95] Kupers R, Maeyaert J, Boly M, Faymonville ME, Laureys S. Naloxone-insensitive epidural placebo analgesia in a chronic pain patient. Anesthesiology 2007;106(6):1239-1242.
[96] Lander J, Fowler-Kerry S. TENS for children's procedural pain. Pain 1993;52(2):209-216.
[97] Leibing E, Leonhardt U, Koster G, Goerlitz A, Rosenfeldt J, Hilgers R, Ramadori G. Acupuncture treatment of chronic low-back pain - a randomized, blinded, placebo-controlled trial with 9-month follow-up. Pain 2002;96 189-196.
[98] Levine JD, Gordon NC. Influence of the method of drug administration on analgesic response. Nature 1984;312(5996):755-756.
[99] Licciardone JC, Stoll ST, Fulda KG, Russo DP, Siu J, Winn W, Swift JJ. Osteopathic Manipulative Treatment for Chronic Low Back Pain: A Randomized Controlled Trial. Spine 2003;28(13):1355-1362.
[100] Lieberman MD, Jarcho JM, Berman S, Naliboff BD, Suyenobu BY, Mandelkern M, Mayer EA. The neural correlates of placebo effects: a disruption account. Neuroimage 2004;22(1):447-455.
[101] Limoges MF, Rickabaugh B. Evaluation of TENS During Screening Flexible Sigmoidoscopy. Gastroenterol Nurs 2004;27(2):61-68.
[102] Lin J-G, Lo M-W, Wen Y-R, Hsieh C-L, Tsai S-K, Sun W-Z. The effect of high and low frequency electroacupuncture in pain after lower abdominal surgery. Pain 2002;99(3):509-514.
[103] Linde K, Streng A, Jurgens S, Hoppe A, Brinkhaus B, Witt C, Wagenpfeil S, Pfaffenrath V, Hammes MG, Weidenhammer W, Willich SN, Melchart D. Acupuncture for patients with migraine. A randomized controlled trial. Journal of the American Medical Association 2005;293 (2118):2125.
[104] Liossi C, Hatira P. Clinical Hypnosis in the Alleviation of Procedure-Related Pain in Pediatric Oncology Patients. Int J Clin Exp Hypn 2003;51(1):4-28.
[105] Lipman JJ, Miller BE, Mays KS, Miller MN, North WC, Byrne WL. Peak B endorphin concentration in cerebrospinal fluid: reduced in chronic pain patients and increased during the placebo response. Psychopharmacology (Berl) 1990;102(1):112-116.
[106] Manchikanti L, Pampati V, Damron K. The role of placebo and nocebo effects of perioperative administration of sedatives and opioids in interventional pain management. Pain Physician 2005;8(4):349-355.
[107] Martikainen IK, Hagelberg N, Mansikka H, Hietala J, Någren K, Scheinin H, Pertovaara A. Association of striatal dopamine D2/D3 receptor binding potential with pain but not tactile sensitivity or placebo analgesia. Neurosci Lett 2005;376(3):149-153.
[108] Matre D, Casey KL, Knardahl S. Placebo-Induced Changes in Spinal Cord Pain Processing. The Journal of Neuroscience 2006;26(2):559-563.
[109] Mayberg HS, Silva JA, Brannan SK, Tekell JL, Mahurin RK, McGinnis S, Jerabek PA. The functional neuroanatomy of the placebo effect. A J Psychiatry 2002;159(5):728-737.
[110] Melchart D, Streng A, Hoppe A, Brinkhaus B, Witt C, Wagenpfeil S, Pfaffenrath V, Hammes M, Hummelsberger J, Irnich D, Weidenhammer W, Willich SN, Linde K. Acupuncture in patients with tension-type headache: randomised controlled trial. Br Med J 2005;331 376-382.
[111] Molsberger AF, Mau J, Pawelec DB, Winkler J. Does acupuncture improve the orthopedic management of chronic low back pain – a randomized, blinded, controlled trial with 3 months follow up. Pain 2002;99(3):579-587.

[112] Montgomery G, Kirsch I. Mechanisms of Placebo Pain Reduction: An Empirical Investigation. Psychological Science 1996;7(3):174-176.
[113] Montgomery GH, Kirsch I. Classical conditioning and the placebo effect. Pain 1997;72(1-2):107-113.
[114] Morton DL, Watson A, El-Deredy W, Jones AKP. Reproducibility of placebo analgesia: Effect of dispositional optimism. Pain 2009;146(1â€"2):194-198.
[115] Muehlbacher M, Nickel MK, Kettler C, Tritt K, Lahmann C, Leiberich PK, Nickel C, Krawczyk J, Mitterlehner FO, Rother WK, Loew TH, Kaplan P. Topiramate in treatment of patients with chronic low back pain: a randomized, double-blind, placebo-controlled study. Clin J Pain 2006;22(6):526-531.
[116] Nemoto H, Nemoto Y, Toda H, Mikuni M, Fukuyama H. Placebo analgesia: a PET study. Exp Brain Res 2007;179(4):655-664.
[117] Ockene JK, Barad DH, Cochrane BB, Larson JC, Gass M, Wassertheil-Smoller S, Manson JE, Barnabei VM, Lane DS, Brzyski RG, Rosal MC, Wylie-Rosett J, Hays J. Symptom experience after discontinuing use of estrogen plus progestin. JAMA 2005;294(2):183-193.
[118] Pallay RM, Seger W, Adler JL, Ettlinger RE, Quaidoo EA, Lipetz R, O'Brien K, Mucciola L, Skalky CS, Petruschke RA, Bohidar NR, Geba GP. Etoricoxib reduced pain and disability and improved quality of life in patients with chronic low back pain: a 3 month, randomized, controlled trial. Scand J Rheumatol 2004;33(4):257-266.
[119] Pariente J, White P, Frackowiak RSJ, Lewith G. Expectancy and belief modulate the neuronal substrates of pain treated by acupuncture. Neuroimage 2005;25(4):1161-1167.
[120] Peloso PM, Fortin L, Beaulieu A, Kamin M, Rosenthal N. Analgesic efficacy and safety of tramadol/ acetaminophen combination tablets (Ultracet) in treatment of chronic low back pain: a multicenter, outpatient, randomized, double blind, placebo controlled trial. J Rheumatol 2004;31(12):2454-2463.
[121] Petrovic P, Kalso E, Petersson KM, Ingvar M. Placebo and opioid analgesia-- imaging a shared neuronal network. Science 2002;295(5560):1737-1740.
[122] Pollo A, Amanzio M, Arslanian A, Casadio C, Maggi G, Benedetti F. Response expectancies in placebo analgesia and their clinical relevance. Pain 2001;93(1):77-84.
[123] Pollo A, Vighetti S, Rainero I, Benedetti F. Placebo analgesia and the heart. Pain 2003;102(1-2):125-133.
[124] Preyde M. Effectiveness of massage therapy for subacute low-back pain: a randomized controlled trial. Can Med Assoc J 2000;162(13):1815-1820.
[125] Price DD, Craggs J, Nicholas Verne G, Perlstein WM, Robinson ME. Placebo analgesia is accompanied by large reductions in pain-related brain activity in irritable bowel syndrome patients. Pain 2007;127(1):63-72.
[126] Price DD, Long S, Wilsey B, Rafii A. Analysis of peak magnitude and duration of analgesia produced by local anesthetics injected into sympathetic ganglia of complex regional pain syndrome patients. Clin J Pain 1998;14(3):216-226.
[127] Price DD, Milling LS, Kirsch I, Duff A, Montgomery GH, Nicholls SS. An analysis of factors that contribute to the magnitude of placebo analgesia in an experimental paradigm. Pain 1999;83(2):147-156.
[128] Rainville P, Duncan GH, Price DD, Carrier B, Bushnell MC. Pain affect encoded in human anterior cingulate but not somatosensory cortex. Science 1997;277(5328):968-971.
[129] Rawling MJ, Wiebe ER. A randomized controlled trial of fentanyl for abortion pain. Am J Obstet Gynecol 2001;185(1):103-107.
[130] Ristikankare M, Hartikainen J, Heikkinen M, Janatuinen E, Julkunen R. Is routinely given conscious sedation of benefit during colonoscopy? Gastrointest Endosc 1999;49(5):566-572.
[131] Robinson R, Darlow S, Wright SJ, Watters C, Carr I, Gadsby G, Mayberry J. Is transcutaneous electrical nerve stimulation an effective analgesia during colonoscopy? Postgrad Med J 2001;77(909):445-446.

[132] Rowbotham MC, Davies PS, Verkempinck C, Galer BS. Lidocaine patch: double-blind controlled study of a new treatment method for post-herpetic neuralgia. Pain 1996;65(1):39-44.
[133] Ruoff GE, Rosenthal N, Jordan D, Karim R, Kamin M. Tramadol/Acetaminophen combination tablets for the treatment of chronic lower back pain: A multicenter, randomized, double-blind, placebo-controlled outpatient study. Clin Ther 2003;25(4):1123-1141.
[134] Sanders GE, Reinert O, Tepe R, Maloney P. Chiropractic adjustive manipulation on subjects with acute low back pain: visual analog pain scores and plasma beta-endorphin levels. J Manipulative Physiol Ther 1990;13(7):391-395.
[135] Scharf HP, Mansmann U, Streitberger K, Witte S, Kramer J, Maier C, Trampisch HJ, Victor N. Acupuncture and knee osteoarthritis: A three-armed randomized trial. Ann Intern Med 2006;145(1):12-20.
[136] Scharff L, Marcus DA, Masek BJ. A Controlled Study of Minimal-Contact Thermal Biofeedback Treatment in Children With Migraine. J Pediatr Psychol 2002;27(2):109-119.
[137] Schnebel BE, Simmons JW. The use of oral colchicine for low-back pain. A double-blind study. Spine (Phila Pa 1976) 1988;13(3):354-357.
[138] Schnitzer TJ, Gray WL, Paster RZ, Kamin M. Efficacy of tramadol in treatment of chronic low back pain. J Rheumatol 2000;27(3):772-778.
[139] Scott DJ, Stohler CS, Egnatuk CM, Wang H, Koeppe RA, Zubieta J-K. Individual Differences in Reward Responding Explain Placebo-Induced Expectations and Effects. Neuron 2007;55(2):325-336.
[140] Scott DJ, Stohler CS, Egnatuk CM, Wang H, Koeppe RA, Zubieta J. PLacebo and nocebo effects are defined by opposite opioid and dopaminergic responses. Arch Gen Psychiatry 2008;65(2):220-231.
[141] Snyder-Mackler L, Barry AJ, Perkins AI, Soucek MD. Effects of helium-neon laser irradiation on skin resistance and pain in patients with trigger points in the neck or back. Phys Ther 1989;69(5):336-341.
[142] Soriano F, Rios R. Gallium arsenide laser treatment of chronic low back pain: A prospective, randomized and double blind study. Laser Therapy 1998;10(4):175-180.
[143] Stransky M, Rubin A, Lava NS, Lazaro RP. Treatment of carpal tunnel syndrome with vitamin B6: a double-blind study. South Med J 1989;82(7):841-842.
[144] Szpalski M, Hayez JP. Objective functional assessment of the efficacy of tenoxicam in the treatment of acute low back pain. A double-blind placebo-controlled study. Br J Rheumatol 1994;33(1):74-78.
[145] Tashjian RZ, Banerjee R, Bradley MP, Alford W, Fadale PD. Zolpidem reduces postoperative pain, fatigue, and narcotic consumption following knee arthroscopy: a prospective randomized placebo-controlled double-blinded study. J Knee Surg 2006;19(2):105-111.
[146] Theroux MC, West DW, Corddry DH, Hyde PM, Bachrach SJ, Cronan KM, Kettrick RG. Efficacy of intranasal midazolam in facilitating suturing of lacerations in preschool children in the emergency department. Pediatrics 1993;91(3):624-627.
[147] Thomas KS, Muir KR, Doherty M, Jones AC, O'Reilly SC, Bassey EJ. Home based exercise programme for knee pain and knee osteoarthritis: randomised controlled trial. BMJ 2002;325(7367):752.
[148] Toya S, Motegi M, Inomata K, Ohshiro T, Macda T. Report on a computer-randomized double blind clinical trial to determine the effectiveness of the GaAlAs (830 nm) diode laser for pain attenuation in selected pain groups. Laser Therapy 1994;6:143-148.
[149] Tritrakarn T, Lertakyamanee J, Koompong P, Soontrapa S, Somprakit P, Tantiwong A, Jittapapai S. Both EMLA and Placebo Cream Reduced Pain during Extracorporeal Piezoelectric Shock Wave Lithotripsy with the Piezolith 2300. Anesthesiology 2000;92(4):1049-1054.
[150] Vase L, Robinson ME, Verne GN, Price DD. The contributions of suggestion, desire, and expectation to placebo effects in irritable bowel syndrome patients: An empirical investigation. Pain 2003;105(1-2):17-25.

[151] Vase L, Robinson ME, Verne GN, Price DD. Increased placebo analgesia over time in irritable bowel syndrome (IBS) patients is associated with desire and expectation but not endogenous opioid mechanisms. Pain 2005;115(3):338-347.
[152] Verne GN, Robinson ME, Vase L, Price DD. Reversal of visceral and cutaneous hyperalgesia by local rectal anesthesia in irritable bowel syndrome (IBS) patients. Pain 2003;105(1):223-230.
[153] Vondrackova D, Leyendecker P, Meissner W, Hopp M, Szombati I, Hermanns K, Ruckes C, Weber S, Grothe B, Fleischer W, Reimer K. Analgesic efficacy and safety of oxycodone in combination with naloxone as prolonged release tablets in patients with moderate to severe chronic pain. The journal of pain : official journal of the American Pain Society 2008;9(12):1144-1154.
[154] Vorsanger GJ, Xiang J, Gana TJ, Pascual ML, Fleming RR. Extended-release tramadol (tramadol ER) in the treatment of chronic low back pain. J Opioid Manag 2008;4(2):87-97.
[155] Voudouris NJ, Peck CL, Coleman G. Conditioned placebo responses. J Pers Soc Psychol 1985;48(1):47-53.
[156] Voudouris NJ, Peck CL, Coleman G. Conditioned response models of placebo phenomena: further support. Pain 1989;38(1):109-116.
[157] Voudouris NJ, Peck CL, Coleman G. The role of conditioning and verbal expectancy in the placebo response. Pain 1990;43(1):121-128.
[158] Wager TD, Matre D, Casey KL. Placebo effects in laser-evoked pain potentials. Brain Behav Immun 2006;20(3):219-230.
[159] Wager TD, Rilling JK, Smith EE, Sokolik A, Casey KL, Davidson RJ, Kosslyn SM, Rose RM, Cohen JD. Placebo-Induced Changes in fMRI in the Anticipation and Experience of Pain. Science 2004;303(5661):1162-1167.
[160] Wager TD, Scott DJ, Zubieta J-K. Placebo effects on human μ-opioid activity during pain. Proceedings of the National Academy of Sciences 2007;104(26):11056-11061.
[161] Walton RE, Chiappinelli J. Prophylactic penicillin: effect on posttreatment symptoms following root canal treatment of asymptomatic periapical pathosis. J Endod 1993;19(9):466-470.
[162] Wang B, Tang J, White PF, Naruse R, Sloninsky A, Kariger R, Gold J, Wender RH. Effect of the Intensity of Transcutaneous Acupoint Electrical Stimulation on the Postoperative Analgesic Requirement. Anesth Analg 1997;85(2):406-413.
[163] Watson A, El-Deredy W, Bentley DE, Vogt BA, Jones AKP. Categories of placebo response in the absence of site-specific expectation of analgesia. Pain 2006;126(1):115-122.
[164] Watson A, El-Deredy W, Vogt BA, Jones AKP. Placebo analgesia is not due to compliance or habituation: EEG and behavioural evidence. Neuroreport 2007;18(8):771-775 710.1097/WNR.1090b1013e3280c1091e1092a1098.
[165] Webster LR, Butera PG, Moran LV, Wu N, Burns LH, Friedmann N. Oxytrex minimizes physical dependence while providing effective analgesia: a randomized controlled trial in low back pain. The journal of pain : official journal of the American Pain Society 2006;7(12):937-946.
[166] Witt C, Brinkhaus B, Jena S, Linde K, Streng A, Wagenpfeil S, Hummelsberger J, Walther HU, Melchart D, Willich SN. Acupuncture in patients with osteoarthritis of the knee: a randomised trial. Lancet 2005;366 136-143.
[167] Wu M-T, Sheen J-M, Chuang K-H, Yang P, Chin S-L, Tsai C-Y, Chen C-J, Liao J-R, Lai P-H, Chu K-A, Pan H-B, Yang C-F. Neuronal Specificity of Acupuncture Response: A fMRI Study with Electroacupuncture. Neuroimage 2002;16(4):1028-1037.
[168] Zubieta JK, Bueller JA, Jackson LR, Scott DJ, Xu Y, Koeppe RA, Nichols TE, Stohler CS. Placebo Effects Mediated by Endogenous Opioid Activity on æ-Opioid Receptors. The Journal of Neuroscience 2005;25(34):7754-7762.
[169] Zubieta JK, Yau WY, Scott DJ, Stohler CS. Belief or Need? Accounting for individual variations in the neurochemistry of the placebo effect. Brain Behav Immun 2006;20(1):15-26.
