# Supplementary material for: What techniques might be used to harness placebo effects in non-malignant pain? A literature review and survey to develop a taxonomy
Source: BMJ Open. 2017 Jun 30;7(6):e015516. doi: 10.1136/bmjopen-2016-015516 (PMC5734496; doi:10.1136/bmjopen-2016-015516)
Supplement: Author's manuscript [file bmjopen-2016-015516.draft_revisions.pdf]

BMJ Open

A Taxonomy of Techniques with Potential to Harness Placebo Effects in Non-Malignant Pain

Journal:	*BMJ Open*
Manuscript ID	bmjopen-2016-015516
Article Type:	Research
Date Submitted by the Author:	12-Dec-2016
Complete List of Authors:	Bishop, Felicity; University of Southampton, Psychology<br>Coghlan, Beverly; University of Southampton<br>Geraghty, Adam; University of Southampton, Primary Care and Population Sciences<br>Everitt, Hazel; University of Southampton, Primary Care and Population Sciences, Faculty of Medicine<br>Little, Paul; University of Southampton, Primary Care and Population Science;<br>Holmes, Michelle; University of Southampton, Psychology<br>Seretis , Dionysis ; University of Southampton, Psychology<br>Lewith, George; University of Southampton
**Primary Subject Heading**:	Complementary medicine
Secondary Subject Heading:	General practice / Family practice, Research methods, Rheumatology, Rehabilitation medicine
Keywords:	placebos, placebo effect, nocebo effect, translational research, review, classification

SCHOLARONE™
Manuscripts

A Taxonomy of Techniques with Potential to Harness Placebo Effects in Non-Malignant Pain

Running head: Taxonomy of techniques to harness placebo effects

Authors: Felicity L Bishop PhD [a,b], Beverly Coghlan MSc [b], Adam WA Geraghty PhD [b], Hazel Everitt PhD [b], Paul Little FMedSci [b], Michelle M Holmes MRes [a], Dionysis Seretis MRes [a], George Lewith MRCGP [b]

a. Department of Psychology, Faculty of Social Human and Mathematical Sciences, Building 44 Highfield Campus, University of Southampton, Southampton SO17 1BJ United Kingdom.

b. Primary Care and Population Sciences, Aldermoor Health Centre, University of Southampton, Southampton, SO16 5ST, United Kingdom.

Emails: Felicity Bishop: F.L.Bishop@southampton.ac.uk; Beverly Coghlan: beverly101@btinternet.com; Adam Geraghty: A.W.Geraghty@soton.ac.uk; Hazel Everitt: H.A.Everitt@soton.ac.uk; Paul Little: P.Little@soton.ac.uk; Michelle Holmes: M.M.Holmes@soton.ac.uk; Dionysis Seretis: ds2g14@soton.ac.uk; George Lewith: gl3@soton.ac.uk.

Corresponding author: Felicity L Bishop. Email: F.L.Bishop@southampton.ac.uk. Phone: +44 (0)23 8059 9020. Fax: +44 (0)23 8059 4597. Psychology, Faculty of Social and Human Sciences, Building 44 Highfield Campus, University of Southampton, Southampton SO17 1BJ United Kingdom.

For peer review only - http://bmjopen.bmj.com/site/about/guidelines.xhtml

Abstract

Objectives. Placebo effects can be large and clinically meaningful but are seldom fully exploited in clinical practice. This review aimed to facilitate translational research by producing a taxonomy of applicable techniques that could augment placebo analgesia in clinical practice.

Design. Literature review and survey.

Methods. We systematically analysed methods used to elicit placebo effects in 169 clinical and laboratory-based studies involving non-malignant pain, drawn from 7 systematic reviews. In a validation exercise we surveyed 33 leading placebo researchers (M=12 years' research experience, SD=9.8).

Results. The final taxonomy defines 30 procedures that may contribute to placebo effects in clinical and experimental research, proposes 60 possible clinical applications, and classifies procedures into 5 domains: the *Patient's Characteristics and Belief* (5 procedures and 11 clinical applications); the *Practitioner's Characteristics and Beliefs* (2 procedures and 4 clinical applications); the *Healthcare Setting* (8 procedures and 13 clinical applications); *Treatment Characteristics* (8 procedures and 14 clinical applications); and The *Patient-Practitioner Interaction* (7 procedures and 18 clinical applications).

Conclusions. The taxonomy provides a preliminary and novel tool with potential to guide translational research aiming to harness placebo effects for patient benefit in practice.

Keywords: placebos; placebo effect; nocebo effect; translational research; review; classification

Article Summary

Strengths and Limitations of this Study

For peer review only - http://bmjopen.bmj.com/site/about/guidelines.xhtml

- This is a novel attempt to use existing studies to conceptualise the factors that might contribute to placebo effects in clinical trials.
- We drew on both clinical trials and laboratory-based studies of placebo effects, in order to generate a more comprehensive list of factors that might contribute to placebo effects than would be possible by relying on just one literature.
- A systematic approach to data synthesis, based on qualitative research methods, was used to identify and classify procedures that might contribute to placebo effects in clinical trials.
- The development of the taxonomy did not incorporate very recent placebo trials or studies.
- The selection of reviews used to determine which original studies to include in the development process was somewhat arbitrary.

For peer review only

For peer review only - http://bmjopen.bmj.com/site/about/guidelines.xhtml

Introduction

There is compelling evidence that factors other than the active ingredients of treatment can have substantial effects on symptoms, particularly non-malignant pain [1 2]. Such 'placebo effects' can be defined as the physiological and/or psychological changes that result from the meaning a person experiences in a health care setting [3 4]. These effects may be as large as treatment effects [5] and occur throughout medicine, especially when doctors and patients interact with each other. They are not routinely deliberately harnessed for patient benefit in clinical practice [6], possibly because doctors often assume they must deceive patients in order to elicit placebo effects [7 8]. However, this assumption is mistaken because it is not necessary to prescribe placebos in order to elicit placebo effects. For example, the overall analgesic effect of an opioid derives not only from its specific pharmaceutical actions but also from the *meaning* that the patient experiences when consulting the doctor and taking the medicine. The same is true for other types of intervention including physical, surgical, and psychotherapies. One approach that has received initial support is for doctors to use positive suggestion to enhance patients' expectations of benefit [9]. Furthermore, preliminary evidence suggests that openly prescribing placebos might elicit clinically meaningful placebo effects in IBS and depression [10 11].

Many techniques and procedures contribute to placebo effects and could potentially be simply and ethically adapted for clinical practice, subject to further testing in practice settings [12]. In order to identify and describe such techniques, and thus provide some direction for future research, we reviewed experimental and clinical studies of placebo effects in non-malignant pain. We focused on non-malignant pain because it can be difficult to manage (particularly with current concerns about opioids [13]), the mechanisms underpinning placebo analgesia are reasonably well understood [14], laboratory-based experimental studies often focus on placebo analgesia, and patients with pain have been shown to display substantial and clinically significant placebo effects [1]. The aim of this project

For peer review only - http://bmjopen.bmj.com/site/about/guidelines.xhtml

was to facilitate translational research by producing a taxonomy of techniques that may contribute to observed placebo effects in research settings and could be studied in future as potential approaches to augmenting placebo enhancement of analgesia in clinical practice.

Methods

Literature Search

We selected seven systematic reviews of different aspects of the placebo literature, chosen to enable the extraction of information on placebo procedures from a broad range of settings - comprehensive reviews [15-17], reviews of placebo effects in clinical populations [2 18] and reviews of laboratory-based experimental placebo studies [19 20]. After removing duplicates and ineligible studies (see Figure 1), 169 studies were used to develop the taxonomy (for list of included studies see Supplementary Digital Content).

For peer review only

For peer review only - http://bmjopen.bmj.com/site/about/guidelines.xhtml

Figure 1: Flowchart Showing Identification of Studies

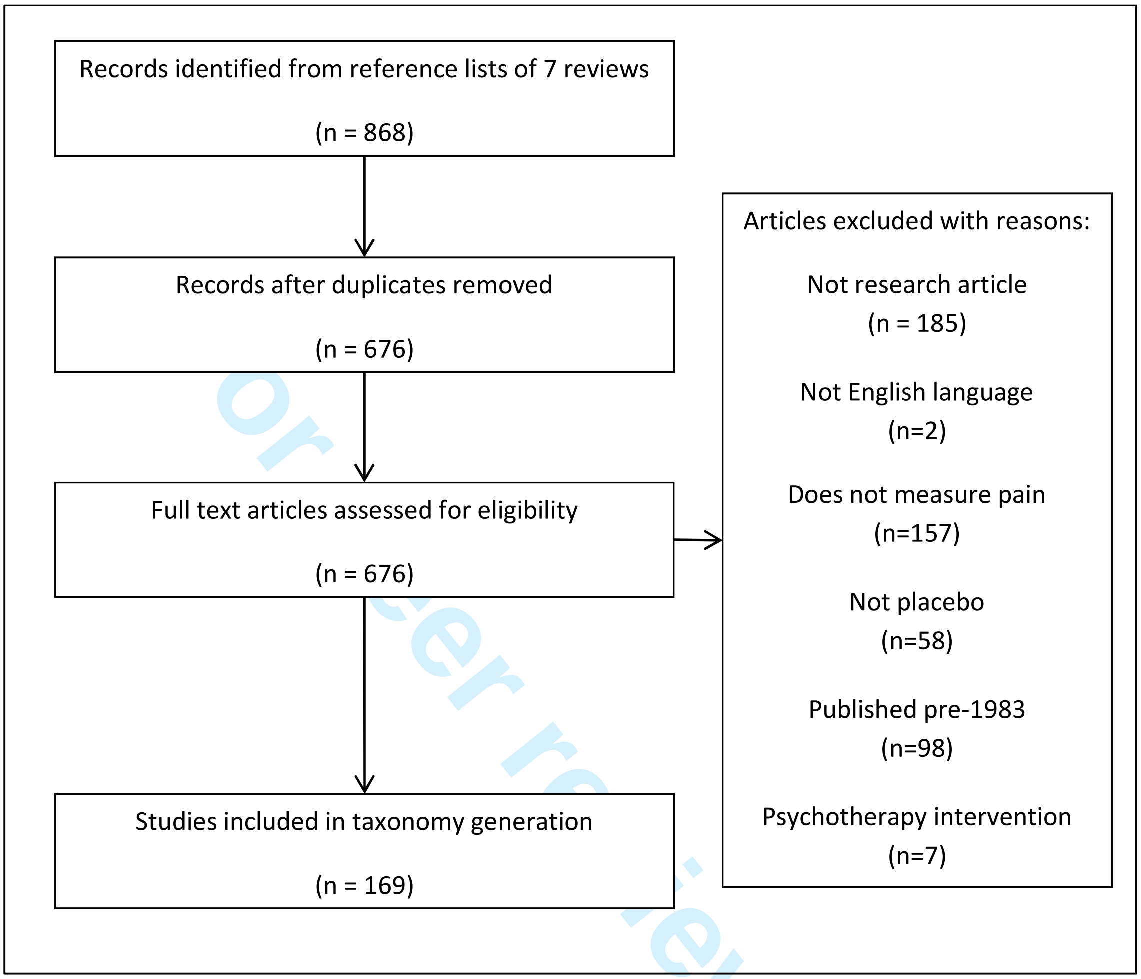

Studies were eligible for inclusion if they: reported original research in which some participants received a placebo intervention, were published since 1983, published in English language, reported a non-malignant pain outcome. Studies were excluded if they: were published before 1983 (because means of generating context-dependent placebo effects may be sensitive to social and cultural changes over time, e.g. patient preferences for particular communication styles and thus their effectiveness in modifying expectations may have changed over time) or examined psychotherapeutic interventions (because it is difficult to disentangle the active ingredients of psychotherapy from the effect of the meaning of the intervention [21]).

For peer review only - http://bmjopen.bmj.com/site/about/guidelines.xhtml

Data Extraction and Synthesis

Descriptions of all events that occurred in the placebo groups during each of the 169 studies (e.g. medical, administrative, and ethical procedures) were extracted into a piloted form by one author and checked by a second. These events were reviewed for duplication and overlap. This resulted in an initial list of 43 procedures that might contribute to placebo effects (e.g. informed consent processes, taking placebo pills, conditioning protocols). Study authors were not contacted for further information about method used.

To synthesise the data and develop our taxonomy items we used systematic and rigorous methods derived from qualitative research. We began with a deductive analysis, which aimed to categorise the procedures in a way that is intuitively appealing, accessible, and clinically relevant by sorting them into five previously-identified contextual domains of healthcare: patients' characteristics/beliefs, practitioners' characteristics/beliefs, doctor-patient relationship, superficial treatment characteristics, and the healthcare environment [22]. We then engaged in a constant comparative analysis, a technique that originates in grounded theory [23]. Procedures and examples of their use were all systematically compared to each other; similar procedures were then merged and all procedures were classified into one of the five domains, the definitions of which were clarified iteratively between all the authors over the course of the classification exercise. This resulted in a more parsimonious list of 29 procedures classified by domain. These 29 procedures were then critically examined to ensure they were theoretically plausible means of producing placebo effects. We focused on three core psychological mechanisms [16 24-27]: response expectancy [28]; conditioning and social learning [29]; and affect, including motivation and anxiety-reduction [15 30]. However, we acknowledge that these mechanisms are difficult to tease apart [31] and that alternative mechanisms have been proposed [4] and so we erred on the side of inclusivity. Neurobiological mechanisms of placebo analgesia have been described [14 32] but a detailed consideration of how

For peer review only

For peer review only - http://bmjopen.bmj.com/site/about/guidelines.xhtml

these might apply to the procedures in the taxonomy would be highly speculative and was beyond the scope of this project (for discussion of clinical applications of the neuroscience of placebo effects see [33]). Four procedures deemed very unlikely to produce placebo effects (e.g. Conveying a Neutral Therapeutic Message) were excluded, leaving 25 procedures that might plausibly contribute to placebo effects. The multidisciplinary team of authors then generated possible clinical applications of each of these 25 procedures.

Validating the Taxonomy

To ensure our taxonomy was comprehensive we surveyed leading placebo researchers (authors of major publications on placebo effects, attendees at an international symposium on placebo effects, and GPs with an interest in placebo effects). Ethical approval for the survey was obtained from the host institution (reference: 4741). Completed electronic surveys including informed consent were received from 33 researchers (52% response rate) experienced in placebo research (M=12 years' experience, SD=9.8). Respondents were shown our draft taxonomy and asked whether, for each domain, they knew of any other procedures that could elicit placebo responses. The proportion answering yes ranged from 22% (Healthcare Setting domain) to 50% (Superficial Treatment Characteristics domain). Respondents suggested 85 additions which were screened against existing items and for theoretical plausibility: 80 of the suggested additions were extra details or suggested clinical applications of existing items; five were new and distinct plausible procedures that were added to the taxonomy, giving a final total of 30 items.

Analysis

The use of each procedure in the 30-item taxonomy was assessed across all 169 studies in the review. Two authors independently rated the presence of each procedure in each study (Kappa = 0.93, discrepancies were resolved through discussion).

For peer review only - http://bmjopen.bmj.com/site/about/guidelines.xhtml

Results

The taxonomy defines 30 procedures that may contribute to placebo effects observed in clinical and experimental research, and classifies them into 5 domains. Table 1 presents the main taxonomy, listing and defining all 30 procedures within 5 domains. Table 2 suggests clinical applications of each procedure. Table 3 shows the frequency of use of each procedure in clinical and experimental studies. Below we describe the procedures within each domain in turn.

For peer review only

For peer review only - http://bmjopen.bmj.com/site/about/guidelines.xhtml

Table 1. Taxonomy of Procedures to Elicit Placebo Effects in Non-Malignant Pain

Procedure Derived from Literature	Definition
**The Patient's Beliefs and Characteristics**	
1. Select Participants Based on Treatment History.	Screen and select participants (or subgroups) against inclusion criteria related to issues such as medical/treatment history, e.g. naive to intervention being tested (not just contraindications).
2. Create Positive Expectancy.	Deliberately and explicitly suggest to participants that the intervention will be effective for them (not as part of informed consent process).
3. Reduce Negative Expectancy. [a]	The potentially negative or harmful procedures and characteristics of the treatment are deliberately minimised in information for participants.
4. Convey a Positive Therapeutic Message through Informed Consent Procedures.	Convey (verbally or in writing) a positive therapeutic message through the content of informed consent. The message might be explicit (e.g. "this intervention is usually effective in most people") or implicit (e.g. "this treatment is an antihypertensive").
5. Harness Socio-cultural Context. [a]	Tailor the intervention according to the participant's social and cultural context and history.
**The Practitioner's Beliefs and Characteristics**	
6. Practitioner Expectancy.	The person delivering the treatment expects it to be effective for the patient.
7. Practitioner's Personal Characteristics.	The practitioner's personal and/or professional characteristics (e.g. status) are modified (through selecting practitioners with different characteristics) and/or emphasised to participants.
**The Healthcare Setting**	
8. Active Recruitment.	Actively seek out and recruit participants (e.g. advertising for specific types of patients, writing personally to individual eligible patients identified through medical records).
9. Active Retention.	Make participants feel valued by attempting to keep them in a study (e.g. contact participants if they miss an appointment, incentivise attendance through monetary or non-monetary gifts).
10. Follow-up.	Assess participants after the intervention/ experiment to assess long-term maintenance or changes in effects over at least 6 months.
11. Follow a Standardised Protocol.	The intervention is delivered according to a set, scientifically-derived, protocol, lending credibility to the intervention (and is therefore not individualised for each participant).
12. Ethical Oversight.	Study practices and procedures are explicitly regulated and monitored by an institutional ethics committee, lending credibility to the intervention.
13. Participating in Research.	Participants know that they are part of research and contributing to the furthering of human knowledge and/or improvement of healthcare for future patients.
14. Symptom Monitoring.	Monitor participants' symptoms using self-report measures, clinician/experimenter assessment, or objective

For peer review only - http://bmjopen.bmj.com/site/about/guidelines.xhtml

	measures repeated over time at least twice; patients are aware of the resulting measurements.
15. Enhanced Environment. [a]	The physical and interpersonal environment where the intervention is delivered is deliberately enhanced.
**Treatment Characteristics**	
16. Sham Intervention – Medication.	An inert substance is administered which is manufactured to appear identical to an active medication (e.g. sugar pill, saline IV, topical agent).
17. Sham Interventions – Physical.	A sham physical intervention is administered which is designed to appear identical to the genuine intervention (e.g. de-activated TENS, non-penetrative acupuncture needles at non-acupuncture points).
18. Sham Interventions – Attention Only.	Participants receive study-specific attention in terms of numbers of visits and time spent with study staff but no additional intervention.
19. Ineffective substances. [a]	Products unlikely to be effective or not indicated are administered (e.g. vitamins in the absence of vitamin deficiency).
20. Use Side-Effects.	Potential side-effects are highlighted such that the participant can interpret them as evidence of a potent intervention.
21. Matched treatments.	To secure blinding, placebo/sham treatments are matched to 'real' treatments (e.g. on mode of administration, dosage, frequency of administration, visual appearance, taste, smell, individual titration procedures).
22. Maximised treatment procedures.	The procedures and characteristics of the treatment are exaggerated, e.g. through high dose, use of colour, high frequency, large pill size, lengthy duration of intervention, ritualistic administration.
23. Conditioning.	A desired response (e.g. pain relief) is paired with an intervention stimulus (e.g. placebo cream) so that the participant associates the response with the stimulus.
**The Patient-Practitioner Interaction**	
24. The Process of Informed Consent.	The participant's formal written and/or verbal informed consent is discussed and obtained.
25. Detailed History.	A detailed personal and/or medical and/or psychosocial history is obtained from the participant.
26. Diagnosis/tests.	Additional tests, examinations, or confirmatory diagnostic procedures are undertaken to establish eligibility for the study.
27. Care.	The practitioner deliberately engages the participant with warmth, compassion and empathy.
28. Patient-Centred Communication. [a]	The practitioner adopts a style of consultation that they consider to be appropriate for a particular patient.
29. Extra Attention.	The participant receives extra attention from being in the study, for example is seen more frequently or for longer than usual.
30. Continuity of Care.	Efforts are made for the same practitioner to see the same participant at each contact.

[a] Items added following survey of researchers.

For peer review only - http://bmjopen.bmj.com/site/about/guidelines.xhtml

Table 2. Suggested Potenial Clinical Applications of Procedures to Elicit Placebo Effects in Non-Malignant Pain, Subject to Further Research

Procedure		Suggested Clinical Applications
**The Patient's Beliefs and Characteristics**		
1.	Select Participants Based on Treatment History.	Stop prescribing interventions of a type that a patient has previously not responded to (e.g. tablets); instead, prescribe a different, new, type of treatment (e.g. psychological therapy).
2.	Create Positive Expectancy.	Tell the patient the intervention is likely to be effective.<br>Elicit patients' treatment and illness beliefs and expectations, and dispel any misconceptions.<br>Empower patients to self-care.
3.	Reduce Negative Expectancy.	Limit emphasis on major potential side effects, and describe how uncommon they are.<br>Hide cessation of analgesia administration (e.g. as in Benedetti [34]), after obtaining advanced consent and ensuring patients are aware they can request additional analgesia if needed.
4.	Convey a Positive Therapeutic Message through Informed Consent Procedures.	Provide written and/or verbal information that conveys a positive therapeutic message about treatment.<br>Provide clear rationale for treatment.<br>Provide patient testimonials and supporting literature / media.
5.	Harness Socio-cultural Context.	Elicit patients' culturally embedded treatment and illness beliefs, preferences and expectations, dispelling any misconceptions.<br>Involve significant others in care.
**The Practitioner's Beliefs and Characteristics**		
6.	Practitioner Expectancy.	Only prescribe a treatment to patients when the prescriber expects it will be effective; communicate that expectation to patients.
7.	Practitioner's Personal Characteristics.	Honour patient preferences for particular practitioners.<br>Use indicators of expertise/high status in offices, in correspondence, and when referring to other practitioners.<br>Ensure the patient is seen by a practitioner whose views/values are congruent with the patient's.
**The Healthcare Setting**		
8.	Active Recruitment.	Actively seek out patients and invite them to attend clinic regarding a particular intervention (as opposed to waiting for patients to present).
9.	Active Retention.	Personally contact patients if they miss an appointment.<br>Use incentives to encourage patients to keep appointments.
10.	Follow-up.	Routinely invite patients to book a follow up appointment after an intervention has finished and prior to repeat prescription.<br>Encourage the patient to take responsibility for and self-manage their condition following an intervention.
11.	Follow a Standardised Protocol.	Use patient-friendly treatment protocols and share with patients where they fit in that protocol.
12.	Ethical Oversight.	Ensure that patients understand that their treatment protocol is

For peer review only - http://bmjopen.bmj.com/site/about/guidelines.xhtml

		sanctioned by a higher authority e.g. NICE.
13.	Participating in Research.	Inform patients that all outcomes and GP performance is audited and can contribute to improved knowledge and treatment for future patients.
14.	Symptom Monitoring.	Ask patients to monitor their symptoms regularly, for example using email, phone apps, web-based systems, paper forms.<br>Assess treatment outcome.<br>Give patients feedback on symptom improvements following monitoring.
15.	Enhanced Environment.	Ensure that the environment is professional, pleasant and peaceful.<br>Employ friendly and helpful support staff.
**Treatment Characteristics**		
16.	Sham Intervention – Medication.	Openly prescribe sham medication.<br>With advanced prior consent, prescribe sham medication.
17.	Sham Interventions – Physical.	Openly prescribe sham physical treatments.<br>With advanced prior consent, prescribe sham physical treatments.
18.	Sham Interventions – Attention Only.	Increase frequency and duration of consultations.
19.	Ineffective substances.	Prescribe substances that are likely not to cause harm but not clearly indicated or substances unlikely to be effective e.g. simple linctus.
20.	Use Side-Effects.	Tell patients about side effects associated with positive clinical outcome.
21.	Matched treatments.	Design appearance of prescribed substance (e.g. colour, packaging, taste) to match known effective treatments.
22.	Maximised treatment procedures.	Within safety limits prescribe higher dose/ higher frequency/ larger pill.<br>Use different colour treatments.<br>Instigate ritualistic procedures patients can perform when taking medicines.<br>Maximise adherence to treatment through education, easy follow up appointments, easy repeat prescription arrangements etc.
23.	Conditioning.	Prescribe highest tolerated dose first, then titrate downwards.<br>With consent, begin with active intervention, pair with a seemingly identical placebo then substitute for placebo alone.(e.g. as in Sandler[35])
**The Patient-Practitioner Interaction**		
24.	The Process of Informed Consent.	Actively seek patient consent.<br>Provide treatment options and encourage the patient to choose from these options if they so desire.
25.	Detailed History.	Take a detailed medical and psychosocial history/update.<br>Ensure the patient feels listened to, e.g. through non-verbal communication and/or capturing information.<br>Ask questions about the meaning of symptoms.
26.	Diagnosis/tests.	Provide a definitive/confident diagnosis.<br>Examine the patient fully.
27.	Care.	Allow patient adequate time to tell their story and listen to them.<br>Validate the patient's concerns.<br>Use non-verbal techniques to convey empathy, compassion, warmth.<br>Use touch judiciously.
28.	Patient-Centred	Individualise consultation style according to a patient's preference e.g.

For peer review only - http://bmjopen.bmj.com/site/about/guidelines.xhtml

	Communication.	collaborative vs authoritative. Engage in collaborative decision-making with the patient. Develop shared treatment goals that you and the patient agree on.
29.	Extra Attention.	Give extra attention to or show more interest in a patient by seeing them more frequently, having longer consultations or visiting at home. Do not rush the patient.
30.	Continuity of Care.	Ensure patient is cared for by the same doctor. Read records before consultation.

Note. Suggestions for clinical applications pending research into effectiveness and ethical acceptability in clinical settings.

For peer review only

Table 3. Use of Procedures in Placebo Groups of Clinical and Experimental Studies

Procedure	% of studies that used each procedure: Experimental (n=58)	Clinical (n=111)
**The Patient's Beliefs and Characteristics**		
1. Select Participants Based on Treatment History.	55%	75%
2. Create Positive Expectancy.	76%	5%
3. Reduce Negative Expectancy.	3%	0%
4. Convey a Positive Therapeutic Message through Informed Consent Procedures	43%	1%
5. Harness Socio-cultural Context.	0%	0%
**The Practitioner's Beliefs and Characteristics**		
6. Practitioner Expectancy.	0%	1%
7. Practitioner's Personal Characteristics.	9%	0%
**The Healthcare Setting**		
8. Active Recruitment.	14%	16%
9. Active Retention.	3%	2%
10. Follow-up.	2%	16%
11. Follow a Standardised Protocol.	85%	63%
12. Ethical Oversight.	78%	69%
13. Participating in Research.	86%	84%
14. Symptom Monitoring.	95%	89%
15. Enhanced Environment.	5%	0%
**Treatment Characteristics**		
16. Sham Intervention – Medication.	71%	55%
17. Sham Interventions – Physical.	33%	41%
18. Sham Interventions – Attention Only.	2%	5%
19. Ineffective substances.	0%	1%
20. Use Side-Effects.	0%	1%
21. Matched treatments.	40%	82%
22. Maximised treatment procedures.	22%	3%
23. Conditioning.	41%	0%
**The Patient-Practitioner Interaction**		
24. The Process of Informed Consent.	88%	77%
25. Detailed History.	19%	33%
26. Diagnosis/tests.	36%	41%
27. Care.	0%	1%
28. Patient-Centred Communication.	0%	0%
29. Extra Attention.	2%	63%
30. Continuity of Care.	7%	14%

For peer review only - http://bmjopen.bmj.com/site/about/guidelines.xhtml

The Patient's Characteristics and Beliefs

The taxonomy specifies five procedures that act directly on the patient's characteristics and/or beliefs in ways that might contribute to placebo effects. Procedure 1 involves selecting patients who are most likely to benefit from an intervention based on their history of similar treatments (where similarity is construed broadly at multiple levels, including for example appearance, modality, style, and pharmacology). For example, one might select those patients who have not experienced disappointing results from a similar intervention in the past (as this group might have learned to expect the intervention to fail). This procedure was commonly used by clinical trials and (to a lesser degree) experimental studies.

Procedures 2 (create positive expectancy), 3 (reduce negative expectancy), and 4 (convey a positive therapeutic message), all involve communicating with patients to encourage them to expect beneficial effects of treatment or not to expect side-effects. The majority of experimental studies in our review explicitly encouraged participants to expect treatment benefits, while very few clinical studies explicitly targeted patients' expectations and hardly any studies at all attempted to minimise participants' expectations of side-effects. Procedure 5 involves tailoring the intervention to the patient's socio-cultural context. This approach emerged from the expert feedback and while it seems plausible and ethical to translate into clinical practice, it was not used by any of the reviewed studies.

The procedures in the patients' beliefs and characteristics domain are thought to contribute to placebo effects primarily through altering patients' response expectancy. Selecting patients based on treatment history and tailoring to socio-cultural context are also predicated on learning mechanisms, i.e., learned associations between treatment outcome and treatment properties.

For peer review only

For peer review only - http://bmjopen.bmj.com/site/about/guidelines.xhtml

There is some evidence that clinicians can give verbal suggestions to alter patients' expectations in practice and in doing so to reduce patients' pain, particularly acute procedural pain [9 36]. As part of work to implement these procedures more widely in practice it would be important to investigate how to secure ethically valid consent for treatment. For example, clinicians might want to encourage realistically positive patient expectations while providing information about possible harms without inducing the negative expectations that could trigger nocebo effects [37 38].

The Practitioner's Characteristics and Beliefs

The two procedures in this domain are about using or modifying health care practitioners' characteristics and/or beliefs. Procedure 6 requires a practitioner to expect a treatment to benefit the patient. This might contribute to observed placebo effects in patients by influencing a practitioner's communication about the treatment and hence a patient's response expectations and/or affective response to the consultation. Only 1% of clinical studies and no experimental studies reported modifying practitioners' expectations. This procedure has received little attention in the placebo literature but clinical research in musculoskeletal settings suggests practitioners' outcome expectations can predict patients' pain outcomes [39]. One way to implement this procedure in practice would be for practitioners to communicate explicitly that they believe a treatment is effective, an approach which clearly overlaps with communication interventions designed to help doctors encourage patients to have positive expectations. Implementing Procedure 6 also depends on practitioners having relevant high quality evidence readily available and accessible and understanding this evidence as it applies to the patient.

A small proportion of studies (9% of experimental studies and no clinical studies) emphasised a practitioner's status or other characteristics (Procedure 7). For some patients a high status practitioner might elicit more confidence in the treatment (and thus higher expectations) and/or a

For peer review only - http://bmjopen.bmj.com/site/about/guidelines.xhtml

more positive affective response to the consultation [40]. Some aspects of this procedure are already part of clinical practice, for example the routine display of medical certificates in doctors' offices; others are inherent in the tools of the doctor, such as the symbolic properties of the stethoscope [41]. However, there is likely to be scope for testing their effects and augmenting their use if appropriate.

The Healthcare Setting

Procedures 8 and 9 relate to the active efforts made in studies to recruit and retain participants respectively. Clinical and experimental studies both reportedly used these procedures sparingly (<20% for active recruitment and <5% for active retention). Such efforts may make participants feel valued and could be implemented in practice through the use of personalised communications from practitioners to encourage attendance at appointments.

Three of the eight procedures in this domain were used by over half of clinical and experimental studies and relate to basic structural features of research: following a protocol, ethical oversight, and participating in research (items 11 to 13). They are thought to impact patients' expectations, by emphasising the legitimacy of the intervention that is being provided and the importance of the patient's contribution to a bigger project, i.e. generating knowledge. Translating these procedures into practice could involve, for example, clinicians explicitly talking with patients about official guidance and treatment protocols that they are following.

Symptom monitoring (item 14) was commonly used in both clinical and experimental studies. This could be implemented in practice for example through repeatedly using patient reported outcome measures (see [42]) and might contribute to placebo effects through learning mechanisms (e.g. regular symptom monitoring acts as feedback to motivate health behaviours and/or modify patients' goals).

For peer review only - http://bmjopen.bmj.com/site/about/guidelines.xhtml

Alternatively, the mere act of asking a patient to monitor their symptoms could convey an expectation of treatment benefit, altering the meaning of a clinical interaction for the patient. Traditionally such effects of the act of measurement are dismissed as Hawthorne effects but they may also be encompassed in broader definitions of placebo effects as meaning effects [43] and could thus enhance effects in clinical practice despite being considered a nuisance in clinical research.

Very few placebo studies (5% of experimental and no clinical studies) reported enhancing the physical or interpersonal environment (item 15). There is a separate and distinct literature on environment modifications in health settings that might be usefully integrated with the placebo literature when developing clinical applications in this area and modelling mechanisms of action [44 45].

Treatment Characteristics

Eight procedures in the taxonomy involve modifying the characteristics of a treatment. Three involve prescribing sham interventions (sham medication – item 16, sham physical interventions – item 17, and extra attention – item 18) while a fourth involves prescribing a substance unlikely to be effective for the symptom in question (item 19). These four procedures represent variations in control conditions used in research and were frequently used by both clinical and experimental studies (with the exception of extra attention which was only used by 2-5% of studies). Such controls are thought to operate primarily via expectations, while affective pathways may also be important when extra attention from trial personnel/medical staff is involved. Of all the procedures in the taxonomy these four that represent control conditions come closest to the traditional notion of how placebos could be applied in practice. Given ethical concerns around deceptive prescribing we suggest that translational research might continue to focus on openly prescribing sham interventions including placebo pills (as in [10 11]). Other options should not be dismissed entirely though: advanced consent and even waiving consent are acceptable to some patients and so it is

For peer review only - http://bmjopen.bmj.com/site/about/guidelines.xhtml

vital for translational research to continue exploring patients', practitioners', and other stakeholders' views on the acceptability and ethics of diverse ways of prescribing placebos [46-49].

Three procedures in this domain modify the superficial (non-pharmacological or non-defining) characteristics of treatments. Procedure 20 is to highlight treatment side-effects to patients in order to encourage patients to see the treatment as potent; this item was very rare, used by only 1% of clinical studies. Procedure 21 was much more commonly used and involves matching the appearance of real and control treatments (used by 40% of experimental and 82% of clinical studies), in order to maintain patient blinding. This could be translated into clinical practice by designing the appearance of interventions to match patients' beliefs about what effective interventions look like. Procedure 22 involves maximising or exaggerating the superficial characteristics of treatment in order to generate larger placebo effects for example by using colour, large pill size, or ritualistic administration of medicines, manipulations which could alter the meaning of a treatment for a patient and/or enhance their expectations. 22% of experimental studies reported using this procedure and one way to translate it into practice would be to create (and test) ritualistic procedures for patients to engage in when taking medicines.

For peer review only

The final procedure in this domain – item 23, conditioning to generate placebo effects - was used commonly and exclusively by experimental studies (41%). Conditioning protocols generate placebo effects through learning mechanisms and perhaps could be implemented in practice to reduce pharmaceutical dosages, as was achieved in a pilot study in children with attention deficit disorder [35].

The Patient-Practitioner Interaction

For peer review only - http://bmjopen.bmj.com/site/about/guidelines.xhtml

The *Patient-Practitioner Interaction* domain incorporates seven procedures related to the interpersonal relationship or interactions between a patient and their health care practitioner. These procedures are thought to operate primarily through affective mechanisms such as reduced anxiety after telling one's story and being listened to with empathy and acknowledged. Three procedures are about specific processes that can occur during consultations – obtaining informed consent (item 24), taking a detailed history (item 25), and performing additional diagnoses or tests (item 26). Arguably these procedures indicate to the patient that the practitioner respects them, is interested in their perspective, and is thorough in their diagnosis. They occur in both clinical and experimental research settings and could be relatively directly translated into practice or optimised if already used.

Two procedures are about the way in which the practitioner engages with the patient: communicating care (item 27), and patient-centred communication (item 28). These procedures were surprisingly very rarely described in the studies included in our review, although recently the nocebo effects of *not* validating a patient's experiences have been shown to be particularly potent [50]. There is of course a distinct and large literature on doctor-patient communication and fruitful dialogue is beginning to bridge these fields [51].

The final two procedures in this domain refer to more structural aspects of consultations: extra attention (item 29, i.e. longer or more frequent appointments) and continuity of care (item 30). 63% of clinical studies used extra attention while a small proportion of clinical (14%) and experimental (7%) studies reported providing continuity of care. Directly implementing these procedures in practice might be challenging given ever increasing constraints on healthcare resources and drives to reduce cost.

For peer review only - http://bmjopen.bmj.com/site/about/guidelines.xhtml

Discussion

The taxonomy names and describes 30 procedures that may contribute to placebo effects in experimental and clinical studies and classifies them into five domains. It includes 60 theoretically plausible techniques that might be applied clinically, subject to further research on their effectiveness and ethical acceptability in practice. Some of the clinical applications derived from the placebo literature have already been investigated in their own right under other auspices, highlighting the need for the burgeoning translational science of placebo effects to be broad-ranging and interdisciplinary.

We have used rigorous systematic review and qualitative analytic methods complemented by a survey to develop the taxonomy. Investigators often combine multiple techniques in any one 'placebo' (e.g. Create Positive Expectancy + Detailed History + Symptom Monitoring) making it beyond the scope of this project to unpack the effectiveness of individual techniques. Procedures did not always fit neatly into single domains. For example, "Screen for Treatment History" was used to select patients for studies of specific treatments (and was thus placed in the Patient's Beliefs and Characteristics domain), but its clinical application involves selecting a treatment for a specific patient and so could be considered a Treatment Characteristic. Conceptually we would expect interactions between these domains; for example, some procedures categorised in other domains probably operate through causal pathways involving patients' beliefs as proximal determinants of placebo effects [63]. We feel the benefits of having a hierarchical structure (modifiable as the taxonomy is refined with use) outweigh the difficulties inherent in classification. We could have used many published reviews of placebo studies in non-malignant pain to identify original studies to review. Selecting seven such reviews means not using others, thus we might have missed original studies that would have suggested additional procedures. Surveying leading researchers and

For peer review only - http://bmjopen.bmj.com/site/about/guidelines.xhtml

incorporating their suggestions somewhat mitigates this limitation. The reviews that we selected as the source of our papers and the papers themselves are now somewhat old examples of the literature. Future work should review very recent papers and iteratively improve the taxonomy accordingly.

This review extends previous work by Di Blasi et al. [22], building on their five domains to systematically develop a detailed taxonomy. We provide a new overarching framework that avoids the controversial and limited distinction between pure and impure placebos [52 53] and integrates ideas from the rich clinical and experimental literatures on placebo effects in non-malignant pain. Many of the components we have identified are likely to be important in other placebo-responsive conditions including depression [54], irritable bowel syndrome [55 56], and insomnia [57]. This taxonomy can guide two important and related applied research agendas: 1) to understand the components of placebo effects in clinical settings [40 58-61] and 2) to ethically harness evidence-based placebo effects to benefit patients [10 11 62]. We hope future studies might draw on the taxonomy to fully describe their methods and develop new applications, thus facilitating future systematic reviews and the development of a systematic and theory driven cumulative evidence-base in this complex field.

The taxonomy identifies and classifies procedures that may contribute to placebo effects in clinical trials and experiments, providing an overarching framework for individual components. However, we do not suggest that every technique in this taxonomy will produce a placebo effect in every patient and we do not know from this project which techniques are more effective or how they might be combined to form ethically acceptable and effective complex interventions. This taxonomy provides the first attempt at a necessary conceptual tool to facilitate future research on these questions. For example, systematic reviews could use the taxonomy to code procedures in original

For peer review only - http://bmjopen.bmj.com/site/about/guidelines.xhtml

studies, using this information in meta-regression analysis to examine the contribution of different procedures to placebo effects [64]. New clinical trials and experiments could extend existing work by systematically examining and comparing the effects and ethical acceptability of different procedures in the taxonomy, building a cumulative evidence-base that has real pragmatic applicability to clinical practice. Some of the suggested clinical applications have been investigated more extensively in other literatures, in particular doctor-patient communication and the healthcare environment. This emphasises the need for a multi-disciplinary approach to the translation of placebo research into practice. One fruitful way forward would be to draw on placebo theories to develop and test more mechanistic models of complex interventions intended to alter the context of healthcare encounters.

Placebo recipients in clinical trials and experiments are exposed to a large number and variety of procedures, many of which might contribute to measured placebo effects. We have systematically identified and classified these procedures into five domains, defined them and suggested possible clinical applications. The resulting taxonomy is presented as a detailed and systematic guide for future research, which can in turn further refine the taxonomy. Researchers and clinicians can use the taxonomy to help conceptualise investigations of clinical applications of placebo effects for patient benefit.

For peer review only - http://bmjopen.bmj.com/site/about/guidelines.xhtml

Funding

The project “Creating a Taxonomy to Harness the Placebo effect in UK primary care” was funded by the National Institute of Health Research (NIHR) School for Primary Care Research (SPCR) (project number 161). This paper presents independent research funded by the National Institute of Health Research (NIHR). The views expressed are those of the author(s) and not necessarily those of the NHS, the NIHR or the Department of Health. Additional funding for BC was provided by Solent NHS Trust. The funders had no role in design and conduct of the study; collection, management, analysis, and interpretation of the data; preparation, review, or approval of the manuscript; and decision to submit the manuscript for publication.

Acknowledgements

We would like to acknowledge the contribution of all of the researchers who shared their views in the survey, including Przemyslaw Babel PhD, Luana Colloca MD PhD, Professor Michael Doherty, Vanda Faria PhD, Magne Arve Flaten, Dr Sarah Goldingay, Dr John Hughes, Prof. Dr. Robert Juette, Irving Kirsch, Karin Meissner, PD Dr. med., Daniel E Moerman PhD, Meike Shedden Mora PhD, Donald D. Price PhD, Professor Harald Walach. We thank Professor Ted Kaptchuk for comments on an earlier draft of this manuscript.

Competing Interests

All authors have completed the ICMJE uniform disclosure form at http://www.icmje.org/coi_disclosure.pdf and declare: no support from any organisation for the submitted work; no financial relationships with any organisations that might have an interest in the

For peer review only

submitted work in the previous three years, no other relationships or activities that could appear to have influenced the submitted work.

Exclusive Licence

The Corresponding Author has the right to grant on behalf of all authors and does grant on behalf of all authors, a worldwide licence (http://www.bmj.com/sites/default/files/BMJ%20Author%20Licence%20March%202013.doc) to the Publishers and its licensees in perpetuity, in all forms, formats and media (whether known now or created in the future), to i) publish, reproduce, distribute, display and store the Contribution, ii) translate the Contribution into other languages, create adaptations, reprints, include within collections and create summaries, extracts and/or, abstracts of the Contribution and convert or allow conversion into any format including without limitation audio, iii) create any other derivative work(s) based in whole or part on the on the Contribution, iv) to exploit all subsidiary rights to exploit all subsidiary rights that currently exist or as may exist in the future in the Contribution, v) the inclusion of electronic links from the Contribution to third party material where-ever it may be located; and, vi) licence any third party to do any or all of the above.

Contributorship Statement

FB designed and led the study, drafted the manuscript, and is guarantor. FB, GL, AWAG, HE, and PL secured funding for the project. FB designed the study with input and revisions from GL, BC, AWAG, HE, and PL. BC led data collection and analysis with additional data collection and analysis by MH and DS. All authors contributed to data interpretation. FB drafted the manuscript and all authors revised it for important intellectual content. All authors had full access to all of the data in the study and can take responsibility for the integrity of the data and the accuracy of the data analysis.

Data Sharing Statement

No unpublished data available.

For peer review only

References

1. Zhang W, Robertson J, Jones AC, Dieppe PA, Doherty M. The placebo effect and its determinants in osteoarthritis: meta-analysis of randomised controlled trials. *Ann. Rheum. Dis.* 2008;67(12):1716-23.
2. Hrobjartsson A, Gotzsche PC. Placebo interventions for all clinical conditions. *Cochrane Database of Systematic Reviews* 2010;1:Art. No.: CD003974-DOI: 10.1002/14651858.CD003974.pub3.
3. Brody H. The placebo response. *J. Fam. Pract.* 2000;49:649-54.
4. Moerman DE, Jonas WB. Deconstructing the placebo effect and finding the meaning response. *Ann. Intern. Med.* 2002;136(6):471-76.
5. Howick J, Friedemann C, Tsakok M, Watson R, Tsakok T, Thomas J, et al. Are Treatments More Effective than Placebos? A Systematic Review and Meta-Analysis. *PLoS One* 2013;8(5):e62599.
6. Doherty M, Dieppe P. The "placebo" response in osteoarthritis and its implications for clinical practice. *Osteoarthritis Cartilage* 2009;17(10):1255-62.
7. Lichtenberg P, Heresco-Levy U, Nitzan U. The ethics of the placebo in clinical practice. *J. Med. Ethics* 2004;30:551-54.
8. Colloca L, Miller FG. Harnessing the placebo effect: the need for translational research. *Philosophical Transactions of the Royal Society B: Biological Sciences* 2011;366(1572):1922-30.
9. Peerdeman KJ, van Laarhoven AIM, Keij SM, Vase L, Rovers MM, Peters ML, et al. Relieving patients' pain with expectation interventions: a meta-analysis. *Pain* 2016;157(6):1179-91.
10. Kaptchuk TJ, Friedlander E, Kelley JM, Sanchez MN, Kokkotou E, Singer JP, et al. Placebos without deception: A randomized controlled trial in Irritable Bowel Syndrome. *PLoS One* 2010;5(12):e15591.
11. Kelley JM, Kaptchuk TJ, Cusin C, Lipkin S, Fava M. Open-label placebo for major depressive disorder: a pilot randomized-controlled trial. *Psychother. Psychosom.* 2012;81:312-14.
12. Kaptchuk TJ, Miller FG. Placebo Effects in Medicine. *N. Engl. J. Med.* 2015;373(1):8-9.
13. Dowell D, Haegerich TM, Chou R. CDc guideline for prescribing opioids for chronic pain—united states, 2016. *JAMA* 2016;315(15):1624-45.
14. Colloca L, Klinger R, Flor H, Bingel U. Placebo analgesia: psychological and neurobiological mechanisms. *Pain* 2013;154(4):511-4.
15. Price DD, Finniss DG, Benedetti F. A comprehensive review of the placebo effect: Recent advances and current thought. *Annu. Rev. Psychol.* 2008;59:565-90.
16. Finniss DG, Kaptchuk TJ, Miller FG, Benedetti F. Biological, clinical, and ethical advances of placebo effects. *Lancet* 2010;375(9715):686-95.
17. Manchikanti L, Giordano J, Fellows B, Hirsch JA. Placebo and nocebo in interventional pain management: A friend or a foe - or simply foes? *Pain Physician* 2011;14:E157-E75.
18. Puhl AA, Reinhart CJ, Rok ER, Injeyan HS. An examination of the observed placebo effect associated with the treatment of low back pain - a systematic review. *Pain Research & Management* 2011;16(1):45-52.
19. Faria V, Fredrikson M, Furmark T. Imaging the placebo response: A neurofunctional review. *Eur. Neuropsychopharmacol.* 2008;18(7):473-85.
20. Vase L, Petersen GL, Riley Iii JL, Price DD. Factors contributing to large analgesic effects in placebo mechanism studies conducted between 2002 and 2007. *Pain* 2009;145(1-2):36-44.
21. Kirsch I. Placebo psychotherapy: Synonym or oxymoron? *J. Clin. Psychol.* 2005;61(7):791-803.
22. Di Blasi Z, Harkness E, Ernst E, Georgiou A, Kleijnen J. Influence of context effects on health outcomes: a systematic review. *Lancet* 2001;357:757-62.
23. Strauss A, Corbin J. *Basics of Qualitative Research Techniques and Procedures for Developing Grounded Theory*. Thousand Oaks: Sage, 1998.

For peer review only

For peer review only - http://bmjopen.bmj.com/site/about/guidelines.xhtml

24. Meissner K, Kohls N, Colloca L. Introduction to placebo effects in medicine: mechanisms and clinical implications. *Philosophical Transactions of the Royal Society B: Biological Sciences* 2011;366(1572):1783-89.
25. Price DD, Chung SK, Robinson ME. Conditioning, expectation, and desire for relief in placebo analgesia. *Seminars in Pain Medicine* 2005;3(1):15-21.
26. Benedetti F, Amanzio M. The placebo response: How words and rituals change the patient's brain. *Patient Educ. Couns.* 2011;84(3):413-19.
27. Goffaux P, L,onard G, Marchand S, Rainville P. Placebo analgesia. In: Beaulieu P, Lussier D, Porreca F, Dickenson A, editors. *Pharmacology of Pain*. Seattle, WA: IASP Press, 2010:451-73.
28. Kirsch I. Response expectancy theory and application: A decennial review. *Appl Prev Psychol* 1997;6(2):69-79.
29. Colloca L, Miller FG. How placebo responses are formed: a learning perspective. *Philosophical Transactions of the Royal Society B: Biological Sciences* 2011;366(1572):1859-69.
30. Hyland ME, Whalley B. Motivational concordance: An important mechanism in self-help therapeutic rituals involving inert (placebo) substances. *J. Psychosom. Res.* 2008;65(5):405-13.
31. Stewart-Williams S, Podd J. The placebo-effect: Dissolving the expectancy versus conditioning debate. *Psychol. Bull.* 2004;130 (2):324-40.
32. Benedetti F. Placebo and the New Physiology of the Doctor-Patient Relationship. *Physiol. Rev.* 2013;93(3):1207-46.
33. Jubb J, Bensing JM. The sweetest pill to swallow: How patient neurobiology can be harnessed to maximise placebo effects. *Neurosci. Biobehav. Rev.* 2013;37(10, Part 2):2709-20.
34. Benedetti F, Maggi G, Lopiano L, Lanotte M, Rainero I, Vighetti S, et al. Open versus hidden medical treatments: The patient's knowledge about a therapy affects the therapy outcome. *Prevention & Treatment* 2003;6(1):No.
35. Sandler AD, Bodfish JW. Open-label use of placebos in the treatment of ADHD: a pilot study. *Child. Care. Health Dev.* 2008;34(1):104-10.
36. Mistiaen P, van Osch M, van Vliet L, Howick J, Bishop FL, Di Blasi Z, et al. The effect of patient-practitioner communication on pain: a systematic review. *Eur J Pain* 2015.
37. Planès S, Villier C, Mallaret M. The nocebo effect of drugs. *Pharmacology Research & Perspectives* 2016;4(2):n/a-n/a.
38. Colloca L, Finniss D. Nocebo effects, patient-clinician communication, and therapeutic outcomes. *JAMA* 2012;307(6):567-8.
39. Witt CM, Martins F, Willich SN, Sch tzler L. Can I help you? Physicians' expectations as predictor for treatment outcome. *European Journal of Pain* 2012;16(10):1455-66.
40. White P, Bishop FL, Prescott P, Scott C, Little P, Lewith G. Practice, practitioner or placebo? A multifactorial, mixed methods randomized controlled trial of acupuncture. *Pain* 2012;153:455-62.
41. Rice T. 'The hallmark of a doctor': the stethoscope and the making of medical identity. *Journal of Material Culture* 2010;15(3):287-301.
42. Snyder CF, Aaronson NK, Choucair AK, Elliott TE, Greenhalgh J, Halyard MY, et al. Implementing patient-reported outcomes assessment in clinical practice: a review of the options and considerations. *Qual. Life Res.* 2012;21(8):1305-14.
43. Benedetti F, Carlino E, Piedimonte A. Increasing uncertainty in CNS clinical trials: the role of placebo, nocebo, and Hawthorne effects. *The Lancet Neurology*;15(7):736-47.
44. Drahota A, Ward D, Mackenzie H, Stores R, Higgins B, Gal D, et al. Sensory environment on health-related outcomes of hospital patients (Review). *Cochrane Database of Systematic Reviews* 2012;3:Art. No.: CD005315. DOI: 10.1002/14651858.CD005315.pub2.
45. Dijkstra K, Pieterse M, Pruyn A. Physical environmental stimuli that turn healthcare facilities into healing environments through psychologically mediated effects: systematic review. *J. Adv. Nurs.* 2006;56(2):166-81.

For peer review only - http://bmjopen.bmj.com/site/about/guidelines.xhtml

For peer review only

46. Feffer K, Lichtenberg P, Becker G, Bloch Y, Netzer R, Nitzan U. A comparative study with depressed patients on the acceptability of placebo use. *Gen. Hosp. Psychiatry* 2016;41:53-6.
47. Ortiz R, Chandros Hull S, Colloca L. Patient attitudes about the clinical use of placebo: qualitative perspectives from a telephone survey. *BMJ Open* 2016;6(4).
48. Bishop FL, Howick J, Heneghan C, Wolstenholme J, Stevens S, Hobbs FDR, et al. Placebo use in the United Kingdom: a qualitative study exploring GPs' views on placebo effects in clinical practice. *Fam. Pract.* 2014;Advance Access:1-7. doi:10.1093/fampra/cmu016.
49. Bishop FL, Aizlewood L, Adams AEM. When and Why Placebo-Prescribing Is Acceptable and Unacceptable: A Focus Group Study of Patients' Views. *PLoS One* 2014;9(7):e101822.
50. Greville-Harris M, Dieppe P. Bad Is More Powerful than Good: The Nocebo Response in Medical Consultations. *The American Journal of Medicine* 2015;128(2):126-29.
51. Bensing JM, Verheul W. The silent healer: The role of communication in placebo effects. *Patient Educ. Couns.* 2010;80(3):293-99.
52. Fassler M, Gnadinger M, Rosemann T, Biller-Andorno N. Use of placebo interventions among Swiss primary care providers. *BMC Health Serv Res* 2009;9(1):144.
53. Howick J, Bishop FL, Heneghan C, Wolstenholme J, Stevens S, Hobbs FDR, et al. Placebo use in the United Kingdom: results from a national survey of primary care practitioners. *PLoS One* 2013;8(3):e58247. doi:10.1371/journal.pone.0058247.
54. Kirsch I, Sapirstein G. Listening to prozac but hearing placebo: A meta-analysis of antidepressant medication. *Prevention & Treatment* 1998;1 Article 0002a, posted June 26, 1998.
55. Patel SM, Stason WB, Legedza A, Ock SM, Kaptchuk TJ, Conboy L, et al. The placebo effect in irritable bowel syndrome trials: a meta-analysis. *Neurogastroenterol. Motil.* 2005;17(3):332-40.
56. Ford AC, Moayyedi P. Meta-analysis: factors affecting placebo response rate in the irritable bowel syndrome. *Aliment. Pharmacol. Ther.* 2010;32(2):144-58.
57. Bélanger L, Vallières A, Ivers H, Moreau V, Lavigne G, Morin CM. Meta-analysis of sleep changes in control groups of insomnia treatment trials. *J. Sleep Res.* 2007;16(1):77-84.
58. Kaptchuk TJ, Kelley JM, Conboy LA, Davis RB, Kerr CE, Jacobson EE, et al. Components of placebo effect: randomised controlled trial in patients with irritable bowel syndrome. *Br. Med. J.* 2008;336:999-1003.
59. Suarez-Almazor ME, Looney C, Liu Y, Cox V, Pietz K, Marcus DM, et al. A randomized controlled trial of acupuncture for osteoarthritis of the knee: Effects of patient-provider communication. *Arthritis Care Res.* 2010;62(9):1229-36.
60. Kaptchuk TJ, Stason WB, Davis RB, Legedza ATR, Schnyer RN, Kerr CE, et al. Sham device v inert pill: randomised controlled trial of two placebo treatments. *Br. Med. J.* 2006;332(7538):391-94.
61. Vase L, Baram S, Takakura N, Yajima H, Takayama M, Kaptchuk TJ, et al. Specifying the nonspecific components of acupuncture analgesia. *Pain* 2013;154(9):1659-67.
62. Sandler A, Glesne C, Geller G. Children's and parents' perspectives on open-label use of placebos in the treatment of ADHD. *Child. Care. Health Dev.* 2008;34(1):111-20.
63. Kirsch I. Conditioning, expectancy, and the placebo effect: Comment on Stewart-Williams and Podd (2004). *Psychol. Bull.* 2004;130(2):341-43.
64. Michie S, Abraham C, Whittington C, McAteer J, Gupta S. Effective techniques in healthy eating and physical activity interventions: a meta-regression. *Health Psychol.* 2009;28(6):690-701.

For peer review only

For peer review only - http://bmjopen.bmj.com/site/about/guidelines.xhtml

Supplementary Digital Content

List of Included Studies

[1-169]

[1] Alfano AP, Taylor AG, Foresman PA, Dunk PR, McConnell GG, Gillies GT. Static magnetic fields for treatment of fibromyalgia: A randomized controlled trial. . J Altern Complement Med 2001;7(1):53.
[2] Alford JW, Fadale PD. Evaluation of postoperative bupivacaine infusion for pain management after anterior cruciate ligament reconstruction. Arthroscopy-the Journal of Arthroscopic and Related Surgery 2003;19(8):855.
[3] Amanzio M, Benedetti F. Neuropharmacological Dissection of Placebo Analgesia: Expectation-Activated Opioid Systems versus Conditioning-Activated Specific Subsystems. The Journal of Neuroscience 1999;19(1):484-494.
[4] Amanzio M, Pollo A, Maggi G, Benedetti F. Response variability to analgesics: a role for non-specific activation of endogenous opioids. Pain 2001;90(3):205-215.
[5] Amlie E, Weber H, Holme I. Treatment of Acute Low-back Pain with Piroxicam: Results of a Double-blind Placebo-controlled Trial. Spine 1987;12(5):473-476.
[6] Andersen AN, Damm P, Tabor A, Pedersen IM, Harring M. Prevention of Breast Pain and Milk Secretion with Bromocriptine After Second-Trimester Abortion. Acta Obstet Gynecol Scand 1990;69(3):235-238.
[7] Aslaksen PM, Flaten MA. The Roles of Physiological and Subjective Stress in the Effectiveness of a Placebo on Experimentally Induced Pain. Psychosom Med 2008;70(7):811-818.
[8] Atkinson JH, Slater MA, Williams RA, Zisook S, Patterson TL, Grant I, Wahlgren DR, Abramson I, Garfin SR. A placebo-controlled randomized clinical trial of nortriptyline for chronic low back pain. Pain 1998;76(3):287-296.
[9] Bannwarth B, Allaert FA, Avouac B, Rossignol M, Rozenberg S, Valat JP. A randomized, double-blind, placebo controlled triphosphate in study of oral adenosine subacute low back pain. J Rheumatol 2005;32(6):1114-1117.
[10] Basford JR, Sheffield CG, Harmsen WS. Laser therapy: a randomized, controlled trial of the effects of low-intensity Nd:YAG laser irradiation on musculoskeletal back pain. Arch Phys Med Rehabil 1999;80(6):647-652.
[11] Basmajian JV. Acute back pain and spasm. A controlled multicenter trial of combined analgesic and antispasm agents. Spine (Phila Pa 1976) 1989;14(4):438-439.
[12] Benedetti F. The opposite effects of the opiate antagonist naloxone and the cholecystokinin antagonist proglumide on placebo analgesia. Pain 1996;64(3):535-543.
[13] Benedetti F, Amanzio M, Baldi S, Casadio C, Cavallo A, Mancuso M, Ruffini E, Oliaro A, Maggi G. The specific effects of prior opioid exposure on placebo analgesia and placebo respiratory depression. Pain 1998;75(2-3):313-319.
[14] Benedetti F, Amanzio M, Casadio C, Oliaro A, Maggi G. Blockade of nocebo hyperalgesia by the cholecystokinin antagonist proglumide. Pain 1997;71(2):135-140.
[15] Benedetti F, Amanzio M, Maggi G. Potentiation of placebo analgesia by proglumide. The Lancet 1995;346(8984):1231.
[16] Benedetti F, Amanzio M, Vighetti S, Asteggiano G. The Biochemical and Neuroendocrine Bases of the Hyperalgesic Nocebo Effect. The Journal of Neuroscience 2006;26(46):12014-12022.
[17] Benedetti F, Arduino C, Amanzio M. Somatotopic activation of opioid systems by target-directed expectations of analgesia. J Neurosci 1999;19(9):3639-3648.
[18] Benedetti F, Arduino C, Costa S, Vighetti S, Tarenzi L, Rainero I, Asteggiano G. Loss of expectation-related mechanisms in Alzheimer's disease makes analgesic therapies less effective. Pain 2006;121(1):133-144.

For peer review only

[19] Benedetti F, Pollo A, Lopiano L, Lanotte M, Vighetti S, Rainero I. Conscious expectation and unconscious conditioning in analgesic, motor, and hormonal placebo/nocebo responses. The Journal of Neuroscience 2003;23(10):4315-4323.
[20] Benedetti FMD, Amanzio MMS, Casadio CMD, Cavallo AMD, Cianci RMD, Giobbe RMD, Mancuso MMD, Ruffini EMD, Maggi GMD. Control of Postoperative Pain by Transcutaneous Electrical Nerve Stimulation After Thoracic Operations. The Annals of Thoracic Surgery 1997;63(3):773-776.
[21] Berry H, Hutchinson DR. A multicentre placebo-controlled study in general practice to evaluate the efficacy and safety of tizanidine in acute low-back pain. J Int Med Res 1988;16(2):75-82.
[22] Bingel U, Lorenz J, Schoell E, Weiller C, Büchel C. Mechanisms of placebo analgesia: rACC recruitment of a subcortical antinociceptive network. Pain 2006;120(1):8-15.
[23] Birbara CA, Puopolo AD, Munoz DR, Sheldon EA, Mangione A, Bohidar NR, Geba GP. Treatment of chronic low back pain with etoricoxib, a new cyclo-oxygenase-2 selective inhibitor: improvement in pain and disability--a randomized, placebo-controlled, 3-month trial. The journal of pain : official journal of the American Pain Society 2003;4(6):307-315.
[24] Biro P, Meier T, Cummins AS. Comparison of topical anaesthesia methods for venous cannulation in adults. Eur J Pain 1997;1(1):37-42.
[25] Blanchard EB, Appelbaum KA, Radnitz CL, Michultka D, Morrill B, Kirsch C, Hillhouse J, Evans DD, Guarnieri P, Attanasio V, et al. Placebo-controlled evaluation of abbreviated progressive muscle relaxation and of relaxation combined with cognitive therapy in the treatment of tension headache. J Consult Clin Psychol 1990;58(2):210-215.
[26] Blanchard EB, Appelbaum KA, Radnitz CL, Morrill B, Michultka D, Kirsch C, Guarnieri P, Hillhouse J, Evans DD, Jaccard J, et al. A controlled evaluation of thermal biofeedback and thermal biofeedback combined with cognitive therapy in the treatment of vascular headache. J Consult Clin Psychol 1990;58(2):216-224.
[27] Boissel JP, Philippon AM, Gauthier E, Schbath J, Destors JM. Time course of long-term placebo therapy effects in angina pectoris. Eur Heart J 1986;7(12):1030-1036.
[28] Bova JG, Bhattacharjee N, Jurdi R, Bennett WF. Comparison of no medication, placebo, and hyoscyamine for reducing pain during a barium enema. Am J Roentgenol 1999;172(5):1285-1287.
[29] Brinkhaus B, Witt CM, Jena S, Linde K, Streng A, Wagenpfeil S, Irnich D, Walther HU, Melchart D, Willich SN. Acupuncture in patients with chronic low back pain - A randomized controlled trial. Arch Intern Med 2006;166(4):450-457.
[30] Camilleri M, Northcutt AR, Kong S, Dukes GE, McSorley D, Mangel AW. Efficacy and safety of alosetron in women with irritable bowel syndrome: a randomised, placebo-controlled trial. The Lancet 2000;355(9209):1035-1040.
[31] Carbajal R, Chauvet X, Couderc S, Olivier-Martin M. Randomised trial of analgesic effects of sucrose, glucose, and pacifiers in term neonates. BMJ 1999;319(7222):1393-1397.
[32] Carette S, Leclaire R, Marcoux S, Morin F, Blaise GA, St-Pierre A, Truchon R, Parent F, Levesque J, Bergeron V, Montminy P, Blanchette C. Epidural corticosteroid injections for sciatica due to herniated nucleus pulposus. N Engl J Med 1997;336(23):1634-1640.
[33] Charron J, Rainville P, Marchand S. Direct comparison of placebo effects on clinical and experimental pain. Clin J Pain 2006;22(2):204-211.
[34] Cherkin DC, Sherman KJ, Avins AL, et al. A randomized trial comparing acupuncture, simulated acupuncture, and usual care for chronic low back pain. Arch Intern Med 2009;169(9):858-866.
[35] Chrubasik S, Eisenberg E, Balan E, Weinberger T, Luzzati R, Conradt C. Treatment of low back pain exacerbations with willow bark extract: a randomized double-blind study. Am J Med 2000;109(1):9-14.

For peer review only

[36] Chrubasik S, Junck H, Breitschwerdt H, Conradt C, Zappe H. Effectiveness of Harpagophytum extract WS 1531 in the treatment of exacerbation of low back pain: a randomized, placebo-controlled, double-blind study. Eur J Anaesthesiol 1999;16(2):118-129.
[37] Chung SK, Price DD, Verne GN, Robinson ME. Revelation of a personal placebo response: Its effects on mood, attitudes and future placebo responding. Pain 2007;132(3):281-288.
[38] Classen W, Feingold E, Netter P. Influence of sensory suggestibility on treatment outcome in headache patients. Neuropsychobiology 1983;10(1):44-47.
[39] Coats TL, Borenstein DG, Nangia NK, Brown MT. Effects of valdecoxib in the treatment of chronic low back pain: results of a randomized, placebo-controlled trial. Clin Ther 2004;26(8):1249-1260.
[40] Colloca L, Benedetti F. How prior experience shapes placebo analgesia. Pain 2006;124(1-2):126-133.
[41] Colloca L, Benedetti F. Placebo analgesia induced by social observational learning. Pain 2009;144(1-2):28-34.
[42] Colloca L, Petrovic P, Wager TD, Ingvar M, Benedetti F. How the number of learning trials affects placebo and nocebo responses. Pain 2010;151(2):430-439.
[43] Colloca L, Sigaudo M, Benedetti F. The role of learning in nocebo and placebo effects. Pain 2008;136(1-2):211-218.
[44] Conn IG, Marshall AH, Yadav SN, Daly JC, Jaffer M. Transcutaneous electrical nerve stimulation following appendicectomy: the placebo effect. Ann R Coll Surg Engl 1986;68(4):191-192.
[45] Corson SL, Batzer FR, Gocial B, Kelly M, Gutmann JN, Go KJ, English ME. Is paracervical block anesthesia for oocyte retrieval effective? Fertil Steril 1994;62(1):133-136.
[46] Costello M, Ramundo M, Christopher NC, Powell KR. Ethyl Vinyl Chloride Vapocoolant Spray Fails to Decrease Pain Associated with Intravenous Cannulation in Children. Clin Pediatr (Phila) 2006;45(7):628-632.
[47] Coyne PJ, MacMurren M, Izzo T, Kramer T. Transcutaneous electrical nerve stimulator for procedural pain associated with intravenous needlesticks. J Intraven Nurs 1995;18(5):263-267.
[48] Dapas F, Hartman SF, Martinez L, Northrup BE, Nussdorf RT, Silberman HM, Gross H. Baclofen for the treatment of acute low-back syndrome. A double-blind comparison with placebo. Spine (Phila Pa 1976) 1985;10(4):345-349.
[49] De Pascalis V, Chiaradia C, Carotenuto E. The contribution of suggestibility and expectation to placebo analgesia phenomenon in an experimental setting. Pain 2002;96(3):393-402.
[50] deCharms RC, Maeda F, Glover GH, Ludlow D, Pauly JM, Soneji D, Gabrieli JDE, Mackey SC. Control over brain activation and pain learned by using real-time functional MRI. Proc Natl Acad Sci U S A 2005;102(51):18626-18631.
[51] Defrin R, Ariel E, Peretz C. Segmental noxious versus innocuous electrical stimulation for chronic pain relief and the effect of fading sensation during treatment. Pain 2005;115(1):152-160.
[52] Dickens C, Jayson M, Sutton C, Creed F. The relationship between pain and depression in a trial using paroxetine in sufferers of chronic low back pain. Psychosomatics 2000;41(6):490-499.
[53] Ditto B, France CR. The effects of applied tension on symptoms in French-speaking blood donors: a randomized trial. Health Psychol 2006;25(3):433-437.
[54] Ditto B, France CR, Lavoie P, Roussos M, Adler PS. Reducing reactions to blood donation with applied muscle tension: a randomized controlled trial. Transfusion (Paris) 2003;43(9):1269-1275.
[55] Dreiser RL, Marty M, Ionescu E, Gold M, Liu JH. Relief of acute low back pain with diclofenac-K 12.5 mg tablets: a flexible dose, ibuprofen 200 mg and placebo-controlled clinical trial. Int J Clin Pharmacol Ther 2003;41(9):375-385.
[56] Erdogmus CB, Resch KL, Sabitzer R, Muller H, Nuhr M, Schoggl A, Posch M, Osterode W, Ungersbock K, Ebenbichler GR. Physiotherapy-based rehabilitation following disc herniation

For peer review only

operation: results of a randomized clinical trial. Spine (Phila Pa 1976) 2007;32(19):2041-2049.
[57] Faas A, Chavannes AW, van Eijk JT, Gubbels JW. A randomized, placebo-controlled trial of exercise therapy in patients with acute low back pain. Spine (Phila Pa 1976) 1993;18(11):1388-1395.
[58] Fanti L, Gemma M, Passaretti S, Guslandi M, Testoni PA, Casati A, Torri G. Electroacupuncture Analgesia for Colonoscopy: A Prospective, Randomized, Placebo-Controlled Study. Am J Gastroenterol 2003;98(2):312-316.
[59] Forster EL, Kramer JF, Lucy SD, Scudds RA, Novick RJ. EFfect of tens on pain, medications, and pulmonary function following coronary artery bypass graft surgery. CHEST Journal 1994;106(5):1343-1348.
[60] Foster KA, Liskin J, Cen S, Abbott A, Armisen V, Globe D, Knox L, Mitchell M, Shtir C, Azen S. The Trager approach in the treatment of chronic headache: a pilot study. Altern Ther Health Med 2004;10(5):40-46.
[61] Foster NE, Thomas E, Barlas P, Hill JC, Young J, Mason E, Hay EM. Acupuncture as an adjunct to exercise based physiotherapy for osteoarthritis of the knee: randomised controlled trial. BMJ 2007;335(7617):436.
[62] Frega A, Stentella P, Di Renzi F, Gallo G, Palazzetti PL, Del Vescovo M, Ciccarone M, Pachi A. Pain evaluation during carbon dioxide laser vaporization for cervical intraepithelial neoplasia: a randomized trial. Clin Exp Obstet Gynecol 1994;21(3):188-191.
[63] Gale GD, Rothbart PJ, Li Y. Infrared therapy for chronic low back pain: a randomized, controlled trial. Pain Res Manag 2006;11(3):193-196.
[64] Geers A, Helfer S, Weiland P, Kosbab K. Expectations and Placebo Response: A Laboratory Investigation into the Role of Somatic Focus. J Behav Med 2006;29(2):171-178.
[65] Geers AL, Wellman JA, Fowler SL, Helfer SG, France CR. Dispositional optimism predicts placebo analgesia. J Pain 2010;11(11):1165-1171.
[66] Goffaux P, Redmond WJ, Rainville P, Marchand S. Descending analgesia--when the spine echoes what the brain expects. Pain 2007;130(1-2):137-143.
[67] Goodenough B, Kampel L, Champion GD, Laubreaux L, Nicholas MK, Ziegler JB, McInerney M. An investigation of the placebo effect and age-related factors in the report of needle pain from venipuncture in children. Pain 1997;72(3):383-391.
[68] Goodkin K, Gullion CM, Agras WS. A randomized, double-blind, placebo-controlled trial of trazodone hydrochloride in chronic low back pain syndrome. J Clin Psychopharmacol 1990;10(4):269-278.
[69] Grevert P, Albert LH, Goldstein A. Partial antagonism of placebo analgesia by naloxone. Pain 1983;16(2):129-143.
[70] Haake M, Muller HH, Schade-Brittinger C, Basler HD, Schafer H, Maier C, Endres HG, Trampisch HJ, Molsberger A. German acupuncture trials (GERAC) for chronic low back pain. Randomized, multicenter, blinded, parallel-group trial with 3 groups Arch Intern Med 2007;167(17):1892-1898.
[71] Hale ME, Ahdieh H, Ma T, Rauck R. Efficacy and safety of OPANA ER (oxymorphone extended release) for relief of moderate to severe chronic low back pain in opioid-experienced patients: a 12-week, randomized, double-blind, placebo-controlled study. The journal of pain : official journal of the American Pain Society 2007;8(2):175-184.
[72] Hargreaves A, Lander J. Use of Transcutaneous Electrical Nerve Stimulation For Postoperative Pain. Nurs Res 1989;38(3):159-160.
[73] Hargreaves KM, Dionne RA, Mueller GP. Plasma Beta-Endorphin-like Immunoreactivity, Pain and Anxiety Following Administration of Placebo in Oral Surgery Patients. J Dent Res 1983;62(11):1170-1173.
[74] Hashish I, Hai HK, Harvey W, Feinmann C, Harris M. Reduction of postoperative pain and swelling by ultrasound treatment: a placebo effect. Pain 1988;33(3):303-311.

For peer review only

[75] Hashish I, Harvey W, Harris M. Anti-inflammatory effects of ultrasound therapy: evidence for a major placebo effect. Rheumatology (Oxford) 1986;25(1):77-81.

[76] Helms JM. Acupuncture for the management of primary dysmenorrhea. Obstet Gynecol 1987;69(1):51-56.

[77] Hoirus KT, Pfleger B, McDuffie FC, Cotsonis G, Elsangak O, Hinson R, Verzosa GT. A randomized clinical trial comparing chiropractic adjustments to muscle relaxants for subacute low back pain. J Manipulative Physiol Ther 2004;27(6):388-398.

[78] Hong C-Z, Chen Y-C, Pon CH, Yu J. Immediate Effects of Various Physical Medicine Modalities on Pain Threshold of an Active Myofascial Trigger Point. Journal of Musculoskeletal Pain 1993;1(2):37-53.

[79] Hruby G, Ames C, Chen C, Yan Y, Sagar J, Baron P, Landman J. Assessment of efficacy of transcutaneous electrical nerve stimulation for pain management during office-based flexible cystoscopy. Urology 2006;67(5):914-917.

[80] Hyland MR, Webber-Gaffney A, Cohen L, Lichtman SW. Randomized Controlled Trial of Calcaneal Taping, Sham Taping, and Plantar Fascia Stretching for the Short-Term Management of Plantar Heel Pain. J Orthop Sports Phys Ther 2006;36(6):364-371.

[81] Johansen O, Brox J, Flaten MA. Placebo and Nocebo Responses, Cortisol, and Circulating Beta-Endorphin. Psychosom Med 2003;65(5):786-790.

[82] Kaptchuk TJ, Stason WB, Davis RB, Legedza ATR, Schnyer RN, Kerr CE, Stone DA, Nam BH, Kirsch I, Goldman RH. Sham device v inert pill: randomised controlled trial of two placebo treatments. Br Med J 2006;332(7538):391-394.

[83] Katz J, Pennella-Vaughan J, Hetzel RD, Kanazi GE, Dworkin RH. A Randomized, Placebo-Controlled Trial of Bupropion Sustained Release in Chronic Low Back Pain. J Pain 2005;6(10):656-661.

[84] Katz N, Ju WD, Krupa DA, Sperling RS, Bozalis Rodgers D, Gertz BJ, Gimbel J, Coleman S, Fisher C, Nabizadeh S, Borenstein D. Efficacy and safety of rofecoxib in patients with chronic low back pain: results from two 4-week, randomized, placebo-controlled, parallel-group, double-blind trials. Spine (Phila Pa 1976) 2003;28(9):851-858; discussion 859.

[85] Katz N, Rauck R, Ahdieh H, Ma T, Gerritsen van der Hoop R, Kerwin R, Podolsky G. A 12-week, randomized, placebo-controlled trial assessing the safety and efficacy of oxymorphone extended release for opioid-naive patients with chronic low back pain. Curr Med Res Opin 2007;23(1):117-128.

[86] Keltner JR, Furst A, Fan C, Redfern R, Inglis B, Fields HL. Isolating the Modulatory Effect of Expectation on Pain Transmission: A Functional Magnetic Resonance Imaging Study. The Journal of Neuroscience 2006;26(16):4437-4443.

[87] Kerr AR, Drexel CA, Spielman AI. The efficacy and safety of 50 mg penicillin G potassium troches for recurrent aphthous ulcers. Oral Surg Oral Med Oral Pathol Oral Radiol Endod 2003;96(6):685-694.

[88] Ketenci A, Ozcan E, Karamursel S. Assessment of efficacy and psychomotor performances of thiocolchicoside and tizanidine in patients with acute low back pain. Int J Clin Pract 2005;59(7):764-770.

[89] Klaber Moffett JA, Richardson PH, Frost H, Osborn A. A placebo controlled double blind trial to evaluate the effectiveness of pulsed short wave therapy for osteoarthritic hip and knee pain. Pain 1996;67(1):121-127.

[90] Klein RG, Eek BC. Low-energy laser treatment and exercise for chronic low back pain: double-blind controlled trial. Arch Phys Med Rehabil 1990;71(1):34-37.

[91] Kober A, Scheck T, Greher M, Lieba F, Fleischhackl R, Fleischhackl S, Randunsky F, Hoerauf K. Prehospital Analgesia with Acupressure in Victims of Minor Trauma: A Prospective, Randomized, Double-Blinded Trial. Anesth Analg 2002;95(3):723-727 710.1213/00000539-200209000-200200035.

For peer review only

[92] Kong J, Gollub RL, Polich G, Kirsch I, LaViolette P, Vangel M, Rosen B, Kaptchuk TJ. A Functional Magnetic Resonance Imaging Study on the Neural Mechanisms of Hyperalgesic Nocebo Effect. The Journal of Neuroscience 2008;28(49):13354-13362.

[93] Kong J, Gollub RL, Rosman IS, Webb JM, Vangel MG, Kirsch I, Kaptchuk TJ. Brain activity associated with expectancy-enhanced placebo analgesia as measured by functional magnetic resonance imaging. J Neurosci 2006;26(2):381-388.

[94] Kotani N, Kushikata T, Suzuki A, Hashimoto H, Muraoka M, Matsuki A. Insertion of intradermal needles into painful points provides analgesia for intractable abdominal scar pain. Reg Anesth Pain Med 2001;26(6):532-538.

[95] Kupers R, Maeyaert J, Boly M, Faymonville ME, Laureys S. Naloxone-insensitive epidural placebo analgesia in a chronic pain patient. Anesthesiology 2007;106(6):1239-1242.

[96] Lander J, Fowler-Kerry S. TENS for children's procedural pain. Pain 1993;52(2):209-216.

[97] Leibing E, Leonhardt U, Koster G, Goerlitz A, Rosenfeldt J, Hilgers R, Ramadori G. Acupuncture treatment of chronic low-back pain - a randomized, blinded, placebo-controlled trial with 9-month follow-up. Pain 2002;96 189-196.

[98] Levine JD, Gordon NC. Influence of the method of drug administration on analgesic response. Nature 1984;312(5996):755-756.

[99] Licciardone JC, Stoll ST, Fulda KG, Russo DP, Siu J, Winn W, Swift JJ. Osteopathic Manipulative Treatment for Chronic Low Back Pain: A Randomized Controlled Trial. Spine 2003;28(13):1355-1362.

[100] Lieberman MD, Jarcho JM, Berman S, Naliboff BD, Suyenobu BY, Mandelkern M, Mayer EA. The neural correlates of placebo effects: a disruption account. Neuroimage 2004;22(1):447-455.

[101] Limoges MF, Rickabaugh B. Evaluation of TENS During Screening Flexible Sigmoidoscopy. Gastroenterol Nurs 2004;27(2):61-68.

[102] Lin J-G, Lo M-W, Wen Y-R, Hsieh C-L, Tsai S-K, Sun W-Z. The effect of high and low frequency electroacupuncture in pain after lower abdominal surgery. Pain 2002;99(3):509-514.

[103] Linde K, Streng A, Jurgens S, Hoppe A, Brinkhaus B, Witt C, Wagenpfeil S, Pfaffenrath V, Hammes MG, Weidenhammer W, Willich SN, Melchart D. Acupuncture for patients with migraine. A randomized controlled trial. Journal of the American Medical Association 2005;293 (2118):2125.

[104] Liossi C, Hatira P. Clinical Hypnosis in the Alleviation of Procedure-Related Pain in Pediatric Oncology Patients. Int J Clin Exp Hypn 2003;51(1):4-28.

[105] Lipman JJ, Miller BE, Mays KS, Miller MN, North WC, Byrne WL. Peak B endorphin concentration in cerebrospinal fluid: reduced in chronic pain patients and increased during the placebo response. Psychopharmacology (Berl) 1990;102(1):112-116.

[106] Manchikanti L, Pampati V, Damron K. The role of placebo and nocebo effects of perioperative administration of sedatives and opioids in interventional pain management. Pain Physician 2005;8(4):349-355.

[107] Martikainen IK, Hagelberg N, Mansikka H, Hietala J, Någren K, Scheinin H, Pertovaara A. Association of striatal dopamine D2/D3 receptor binding potential with pain but not tactile sensitivity or placebo analgesia. Neurosci Lett 2005;376(3):149-153.

[108] Matre D, Casey KL, Knardahl S. Placebo-Induced Changes in Spinal Cord Pain Processing. The Journal of Neuroscience 2006;26(2):559-563.

[109] Mayberg HS, Silva JA, Brannan SK, Tekell JL, Mahurin RK, McGinnis S, Jerabek PA. The functional neuroanatomy of the placebo effect. A J Psychiatry 2002;159(5):728-737.

[110] Melchart D, Streng A, Hoppe A, Brinkhaus B, Witt C, Wagenpfeil S, Pfaffenrath V, Hammes M, Hummelsberger J, Irnich D, Weidenhammer W, Willich SN, Linde K. Acupuncture in patients with tension-type headache: randomised controlled trial. Br Med J 2005;331 376-382.

[111] Molsberger AF, Mau J, Pawelec DB, Winkler J. Does acupuncture improve the orthopedic management of chronic low back pain – a randomized, blinded, controlled trial with 3 months follow up. Pain 2002;99(3):579-587.

For peer review only

[112] Montgomery G, Kirsch I. Mechanisms of Placebo Pain Reduction: An Empirical Investigation. Psychological Science 1996;7(3):174-176.

[113] Montgomery GH, Kirsch I. Classical conditioning and the placebo effect. Pain 1997;72(1-2):107-113.

[114] Morton DL, Watson A, El-Deredy W, Jones AKP. Reproducibility of placebo analgesia: Effect of dispositional optimism. Pain 2009;146(1â€"2):194-198.

[115] Muehlbacher M, Nickel MK, Kettler C, Tritt K, Lahmann C, Leiberich PK, Nickel C, Krawczyk J, Mitterlehner FO, Rother WK, Loew TH, Kaplan P. Topiramate in treatment of patients with chronic low back pain: a randomized, double-blind, placebo-controlled study. Clin J Pain 2006;22(6):526-531.

[116] Nemoto H, Nemoto Y, Toda H, Mikuni M, Fukuyama H. Placebo analgesia: a PET study. Exp Brain Res 2007;179(4):655-664.

[117] Ockene JK, Barad DH, Cochrane BB, Larson JC, Gass M, Wassertheil-Smoller S, Manson JE, Barnabei VM, Lane DS, Brzyski RG, Rosal MC, Wylie-Rosett J, Hays J. Symptom experience after discontinuing use of estrogen plus progestin. JAMA 2005;294(2):183-193.

[118] Pallay RM, Seger W, Adler JL, Ettlinger RE, Quaidoo EA, Lipetz R, O'Brien K, Mucciola L, Skalky CS, Petruschke RA, Bohidar NR, Geba GP. Etoricoxib reduced pain and disability and improved quality of life in patients with chronic low back pain: a 3 month, randomized, controlled trial. Scand J Rheumatol 2004;33(4):257-266.

[119] Pariente J, White P, Frackowiak RSJ, Lewith G. Expectancy and belief modulate the neuronal substrates of pain treated by acupuncture. Neuroimage 2005;25(4):1161-1167.

[120] Peloso PM, Fortin L, Beaulieu A, Kamin M, Rosenthal N. Analgesic efficacy and safety of tramadol/ acetaminophen combination tablets (Ultracet) in treatment of chronic low back pain: a multicenter, outpatient, randomized, double blind, placebo controlled trial. J Rheumatol 2004;31(12):2454-2463.

[121] Petrovic P, Kalso E, Petersson KM, Ingvar M. Placebo and opioid analgesia-- imaging a shared neuronal network. Science 2002;295(5560):1737-1740.

[122] Pollo A, Amanzio M, Arslanian A, Casadio C, Maggi G, Benedetti F. Response expectancies in placebo analgesia and their clinical relevance. Pain 2001;93(1):77-84.

[123] Pollo A, Vighetti S, Rainero I, Benedetti F. Placebo analgesia and the heart. Pain 2003;102(1-2):125-133.

[124] Preyde M. Effectiveness of massage therapy for subacute low-back pain: a randomized controlled trial. Can Med Assoc J 2000;162(13):1815-1820.

[125] Price DD, Craggs J, Nicholas Verne G, Perlstein WM, Robinson ME. Placebo analgesia is accompanied by large reductions in pain-related brain activity in irritable bowel syndrome patients. Pain 2007;127(1):63-72.

[126] Price DD, Long S, Wilsey B, Rafii A. Analysis of peak magnitude and duration of analgesia produced by local anesthetics injected into sympathetic ganglia of complex regional pain syndrome patients. Clin J Pain 1998;14(3):216-226.

[127] Price DD, Milling LS, Kirsch I, Duff A, Montgomery GH, Nicholls SS. An analysis of factors that contribute to the magnitude of placebo analgesia in an experimental paradigm. Pain 1999;83(2):147-156.

[128] Rainville P, Duncan GH, Price DD, Carrier B, Bushnell MC. Pain affect encoded in human anterior cingulate but not somatosensory cortex. Science 1997;277(5328):968-971.

[129] Rawling MJ, Wiebe ER. A randomized controlled trial of fentanyl for abortion pain. Am J Obstet Gynecol 2001;185(1):103-107.

[130] Ristikankare M, Hartikainen J, Heikkinen M, Janatuinen E, Julkunen R. Is routinely given conscious sedation of benefit during colonoscopy? Gastrointest Endosc 1999;49(5):566-572.

[131] Robinson R, Darlow S, Wright SJ, Watters C, Carr I, Gadsby G, Mayberry J. Is transcutaneous electrical nerve stimulation an effective analgesia during colonoscopy? Postgrad Med J 2001;77(909):445-446.

For peer review only

[132] Rowbotham MC, Davies PS, Verkempinck C, Galer BS. Lidocaine patch: double-blind controlled study of a new treatment method for post-herpetic neuralgia. Pain 1996;65(1):39-44.
[133] Ruoff GE, Rosenthal N, Jordan D, Karim R, Kamin M. Tramadol/Acetaminophen combination tablets for the treatment of chronic lower back pain: A multicenter, randomized, double-blind, placebo-controlled outpatient study. Clin Ther 2003;25(4):1123-1141.
[134] Sanders GE, Reinert O, Tepe R, Maloney P. Chiropractic adjustive manipulation on subjects with acute low back pain: visual analog pain scores and plasma beta-endorphin levels. J Manipulative Physiol Ther 1990;13(7):391-395.
[135] Scharf HP, Mansmann U, Streitberger K, Witte S, Kramer J, Maier C, Trampisch HJ, Victor N. Acupuncture and knee osteoarthritis: A three-armed randomized trial. Ann Intern Med 2006;145(1):12-20.
[136] Scharff L, Marcus DA, Masek BJ. A Controlled Study of Minimal-Contact Thermal Biofeedback Treatment in Children With Migraine. J Pediatr Psychol 2002;27(2):109-119.
[137] Schnebel BE, Simmons JW. The use of oral colchicine for low-back pain. A double-blind study. Spine (Phila Pa 1976) 1988;13(3):354-357.
[138] Schnitzer TJ, Gray WL, Paster RZ, Kamin M. Efficacy of tramadol in treatment of chronic low back pain. J Rheumatol 2000;27(3):772-778.
[139] Scott DJ, Stohler CS, Egnatuk CM, Wang H, Koeppe RA, Zubieta J-K. Individual Differences in Reward Responding Explain Placebo-Induced Expectations and Effects. Neuron 2007;55(2):325-336.
[140] Scott DJ, Stohler CS, Egnatuk CM, Wang H, Koeppe RA, Zubieta J. PLacebo and nocebo effects are defined by opposite opioid and dopaminergic responses. Arch Gen Psychiatry 2008;65(2):220-231.
[141] Snyder-Mackler L, Barry AJ, Perkins AI, Soucek MD. Effects of helium-neon laser irradiation on skin resistance and pain in patients with trigger points in the neck or back. Phys Ther 1989;69(5):336-341.
[142] Soriano F, Rios R. Gallium arsenide laser treatment of chronic low back pain: A prospective, randomized and double blind study. Laser Therapy 1998;10(4):175-180.
[143] Stransky M, Rubin A, Lava NS, Lazaro RP. Treatment of carpal tunnel syndrome with vitamin B6: a double-blind study. South Med J 1989;82(7):841-842.
[144] Szpalski M, Hayez JP. Objective functional assessment of the efficacy of tenoxicam in the treatment of acute low back pain. A double-blind placebo-controlled study. Br J Rheumatol 1994;33(1):74-78.
[145] Tashjian RZ, Banerjee R, Bradley MP, Alford W, Fadale PD. Zolpidem reduces postoperative pain, fatigue, and narcotic consumption following knee arthroscopy: a prospective randomized placebo-controlled double-blinded study. J Knee Surg 2006;19(2):105-111.
[146] Theroux MC, West DW, Corddry DH, Hyde PM, Bachrach SJ, Cronan KM, Kettrick RG. Efficacy of intranasal midazolam in facilitating suturing of lacerations in preschool children in the emergency department. Pediatrics 1993;91(3):624-627.
[147] Thomas KS, Muir KR, Doherty M, Jones AC, O'Reilly SC, Bassey EJ. Home based exercise programme for knee pain and knee osteoarthritis: randomised controlled trial. BMJ 2002;325(7367):752.
[148] Toya S, Motegi M, Inomata K, Ohshiro T, Macda T. Report on a computer-randomized double blind clinical trial to determine the effectiveness of the GaAlAs (830 nm) diode laser for pain attenuation in selected pain groups. Laser Therapy 1994;6:143-148.
[149] Tritrakarn T, Lertakyamanee J, Koompong P, Soontrapa S, Somprakit P, Tantiwong A, Jittapapai S. Both EMLA and Placebo Cream Reduced Pain during Extracorporeal Piezoelectric Shock Wave Lithotripsy with the Piezolith 2300. Anesthesiology 2000;92(4):1049-1054.
[150] Vase L, Robinson ME, Verne GN, Price DD. The contributions of suggestion, desire, and expectation to placebo effects in irritable bowel syndrome patients: An empirical investigation. Pain 2003;105(1-2):17-25.

For peer review only

[151] Vase L, Robinson ME, Verne GN, Price DD. Increased placebo analgesia over time in irritable bowel syndrome (IBS) patients is associated with desire and expectation but not endogenous opioid mechanisms. Pain 2005;115(3):338-347.
[152] Verne GN, Robinson ME, Vase L, Price DD. Reversal of visceral and cutaneous hyperalgesia by local rectal anesthesia in irritable bowel syndrome (IBS) patients. Pain 2003;105(1):223-230.
[153] Vondrackova D, Leyendecker P, Meissner W, Hopp M, Szombati I, Hermanns K, Ruckes C, Weber S, Grothe B, Fleischer W, Reimer K. Analgesic efficacy and safety of oxycodone in combination with naloxone as prolonged release tablets in patients with moderate to severe chronic pain. The journal of pain : official journal of the American Pain Society 2008;9(12):1144-1154.
[154] Vorsanger GJ, Xiang J, Gana TJ, Pascual ML, Fleming RR. Extended-release tramadol (tramadol ER) in the treatment of chronic low back pain. J Opioid Manag 2008;4(2):87-97.
[155] Voudouris NJ, Peck CL, Coleman G. Conditioned placebo responses. J Pers Soc Psychol 1985;48(1):47-53.
[156] Voudouris NJ, Peck CL, Coleman G. Conditioned response models of placebo phenomena: further support. Pain 1989;38(1):109-116.
[157] Voudouris NJ, Peck CL, Coleman G. The role of conditioning and verbal expectancy in the placebo response. Pain 1990;43(1):121-128.
[158] Wager TD, Matre D, Casey KL. Placebo effects in laser-evoked pain potentials. Brain Behav Immun 2006;20(3):219-230.
[159] Wager TD, Rilling JK, Smith EE, Sokolik A, Casey KL, Davidson RJ, Kosslyn SM, Rose RM, Cohen JD. Placebo-Induced Changes in fMRI in the Anticipation and Experience of Pain. Science 2004;303(5661):1162-1167.
[160] Wager TD, Scott DJ, Zubieta J-K. Placebo effects on human μ-opioid activity during pain. Proceedings of the National Academy of Sciences 2007;104(26):11056-11061.
[161] Walton RE, Chiappinelli J. Prophylactic penicillin: effect on posttreatment symptoms following root canal treatment of asymptomatic periapical pathosis. J Endod 1993;19(9):466-470.
[162] Wang B, Tang J, White PF, Naruse R, Sloninsky A, Kariger R, Gold J, Wender RH. Effect of the Intensity of Transcutaneous Acupoint Electrical Stimulation on the Postoperative Analgesic Requirement. Anesth Analg 1997;85(2):406-413.
[163] Watson A, El-Deredy W, Bentley DE, Vogt BA, Jones AKP. Categories of placebo response in the absence of site-specific expectation of analgesia. Pain 2006;126(1):115-122.
[164] Watson A, El-Deredy W, Vogt BA, Jones AKP. Placebo analgesia is not due to compliance or habituation: EEG and behavioural evidence. Neuroreport 2007;18(8):771-775 710.1097/WNR.1090b1013e3280c1091e1092a1098.
[165] Webster LR, Butera PG, Moran LV, Wu N, Burns LH, Friedmann N. Oxytrex minimizes physical dependence while providing effective analgesia: a randomized controlled trial in low back pain. The journal of pain : official journal of the American Pain Society 2006;7(12):937-946.
[166] Witt C, Brinkhaus B, Jena S, Linde K, Streng A, Wagenpfeil S, Hummelsberger J, Walther HU, Melchart D, Willich SN. Acupuncture in patients with osteoarthritis of the knee: a randomised trial. Lancet 2005;366 136-143.
[167] Wu M-T, Sheen J-M, Chuang K-H, Yang P, Chin S-L, Tsai C-Y, Chen C-J, Liao J-R, Lai P-H, Chu K-A, Pan H-B, Yang C-F. Neuronal Specificity of Acupuncture Response: A fMRI Study with Electroacupuncture. Neuroimage 2002;16(4):1028-1037.
[168] Zubieta JK, Bueller JA, Jackson LR, Scott DJ, Xu Y, Koeppe RA, Nichols TE, Stohler CS. Placebo Effects Mediated by Endogenous Opioid Activity on æ-Opioid Receptors. The Journal of Neuroscience 2005;25(34):7754-7762.
[169] Zubieta JK, Yau WY, Scott DJ, Stohler CS. Belief or Need? Accounting for individual variations in the neurochemistry of the placebo effect. Brain Behav Immun 2006;20(1):15-26.

For peer review only

PRISMA 2009 Checklist

Section/topic	#	Checklist item	Reported on page #
**TITLE**			
Title	1	Identify the report as a systematic review, meta-analysis, or both.	n/a
**ABSTRACT**			
Structured summary	2	Provide a structured summary including, as applicable: background; objectives; data sources; study eligibility criteria, participants, and interventions; study appraisal and synthesis methods; results; limitations; conclusions and implications of key findings; systematic review registration number.	n/a
**INTRODUCTION**			
Rationale	3	Describe the rationale for the review in the context of what is already known.	3
Objectives	4	Provide an explicit statement of questions being addressed with reference to participants, interventions, comparisons, outcomes, and study design (PICOS).	n/a
**METHODS**			
Protocol and registration	5	Indicate if a review protocol exists, if and where it can be accessed (e.g., Web address), and, if available, provide registration information including registration number.	n/a
Eligibility criteria	6	Specify study characteristics (e.g., PICOS, length of follow-up) and report characteristics (e.g., years considered, language, publication status) used as criteria for eligibility, giving rationale.	5
Information sources	7	Describe all information sources (e.g., databases with dates of coverage, contact with study authors to identify additional studies) in the search and date last searched.	4
Search	8	Present full electronic search strategy for at least one database, including any limits used, such that it could be repeated.	n/a
Study selection	9	State the process for selecting studies (i.e., screening, eligibility, included in systematic review, and, if applicable, included in the meta-analysis).	4, Fig1
Data collection process	10	Describe method of data extraction from reports (e.g., piloted forms, independently, in duplicate) and any processes for obtaining and confirming data from investigators.	6
Data items	11	List and define all variables for which data were sought (e.g., PICOS, funding sources) and any assumptions and simplifications made.	6
Risk of bias in individual studies	12	Describe methods used for assessing risk of bias of individual studies (including specification of whether this was done at the study or outcome level), and how this information is to be used in any data synthesis.	n/a
Summary measures	13	State the principal summary measures (e.g., risk ratio, difference in means).	n/a
Synthesis of results	14	Describe the methods of handling data and combining results of studies, if done, including measures of consistency (e.g., I^2) for each meta-analysis.	6

For peer review only - http://bmjopen.bmj.com/site/about/guidelines.xhtml

PRISMA 2009 Checklist

Section/topic	#	Checklist item	Reported on page #
Risk of bias across studies	15	Specify any assessment of risk of bias that may affect the cumulative evidence (e.g., publication bias, selective reporting within studies).	n/a
Additional analyses	16	Describe methods of additional analyses (e.g., sensitivity or subgroup analyses, meta-regression), if done, indicating which were pre-specified.	n/a
**RESULTS**			
Study selection	17	Give numbers of studies screened, assessed for eligibility, and included in the review, with reasons for exclusions at each stage, ideally with a flow diagram.	Fig1
Study characteristics	18	For each study, present characteristics for which data were extracted (e.g., study size, PICOS, follow-up period) and provide the citations.	n/a
Risk of bias within studies	19	Present data on risk of bias of each study and, if available, any outcome level assessment (see item 12).	n/a
Results of individual studies	20	For all outcomes considered (benefits or harms), present, for each study: (a) simple summary data for each intervention group (b) effect estimates and confidence intervals, ideally with a forest plot.	n/a
Synthesis of results	21	Present results of each meta-analysis done, including confidence intervals and measures of consistency.	n/a
Risk of bias across studies	22	Present results of any assessment of risk of bias across studies (see Item 15).	n/a
Additional analysis	23	Give results of additional analyses, if done (e.g., sensitivity or subgroup analyses, meta-regression [see Item 16]).	n/a
**DISCUSSION**			
Summary of evidence	24	Summarize the main findings including the strength of evidence for each main outcome; consider their relevance to key groups (e.g., healthcare providers, users, and policy makers).	21
Limitations	25	Discuss limitations at study and outcome level (e.g., risk of bias), and at review-level (e.g., incomplete retrieval of identified research, reporting bias).	22-3
Conclusions	26	Provide a general interpretation of the results in the context of other evidence, and implications for future research.	22-3
**FUNDING**			
Funding	27	Describe sources of funding for the systematic review and other support (e.g., supply of data); role of funders for the systematic review.	1

From: Moher D, Liberati A, Tetzlaff J, Altman DG, The PRISMA Group (2009). Preferred Reporting Items for Systematic Reviews and Meta-Analyses: The PRISMA Statement. PLoS Med 6(7): e1000097. doi:10.1371/journal.pmed1000097

For more information, visit: www.prisma-statement.org.

For peer review only - http://bmjopen.bmj.com/site/about/guidelines.xhtml

BMJ Open

What Techniques Might be Used to Harness Placebo Effects in Non-Malignant Pain? A Literature Review and Survey to Develop a Taxonomy.

Journal:	*BMJ Open*
Manuscript ID	bmjopen-2016-015516.R1
Article Type:	Research
Date Submitted by the Author:	15-Mar-2017

Complete List of Authors:	Bishop, Felicity; University of Southampton, Psychology<br>Coghlan, Beverly; University of Southampton<br>Geraghty, Adam; University of Southampton, Primary Care and Population Sciences<br>Everitt, Hazel; University of Southampton, Primary Care and Population Sciences, Faculty of Medicine<br>Little, Paul; University of Southampton, Primary Care and Population Science;<br>Holmes, Michelle; University of Southampton, Psychology<br>Seretis , Dionysis ; University of Southampton, Psychology<br>Lewith, George; University of Southampton

**Primary Subject Heading**:	Complementary medicine
Secondary Subject Heading:	General practice / Family practice, Research methods, Rheumatology, Rehabilitation medicine
Keywords:	placebos, placebo effect, nocebo effect, translational research, review, classification

SCHOLARONE™ Manuscripts

What Techniques Might be Used to Harness Placebo Effects in Non-Malignant Pain? A Literature Review and Survey to Develop a Taxonomy.

Running head: Taxonomy of techniques to harness placebo effects

Authors: Felicity L Bishop PhD [a,b], Beverly Coghlan MSc [b], Adam WA Geraghty PhD [b], Hazel Everitt PhD [b], Paul Little FMedSci [b], Michelle M Holmes MRes [a], Dionysis Seretis MRes [a], George Lewith MRCGP [b]

a. Department of Psychology, Faculty of Social Human and Mathematical Sciences, Building 44 Highfield Campus, University of Southampton, Southampton SO17 1BJ United Kingdom.

b. Primary Care and Population Sciences, Aldermoor Health Centre, University of Southampton, Southampton, SO16 5ST, United Kingdom.

Emails: Felicity Bishop: F.L.Bishop@southampton.ac.uk; Beverly Coghlan: beverly101@btinternet.com; Adam Geraghty: A.W.Geraghty@soton.ac.uk; Hazel Everitt: H.A.Everitt@soton.ac.uk; Paul Little: P.Little@soton.ac.uk; Michelle Holmes: M.M.Holmes@soton.ac.uk; Dionysis Seretis: ds2g14@soton.ac.uk; George Lewith: gl3@soton.ac.uk.

Corresponding author: Felicity L Bishop. Email: F.L.Bishop@southampton.ac.uk. Phone: +44 (0)23 8059 9020. Fax: +44 (0)23 8059 4597. Psychology, Faculty of Social and Human Sciences, Building 44 Highfield Campus, University of Southampton, Southampton SO17 1BJ United Kingdom.

For peer review only

For peer review only - http://bmjopen.bmj.com/site/about/guidelines.xhtml

Abstract

Objectives. Placebo effects can be clinically meaningful but are seldom fully exploited in clinical practice. This review aimed to facilitate translational research by producing a taxonomy of applicable techniques that could augment placebo analgesia in clinical practice.

Design. Literature review and survey.

Methods. We systematically analysed methods which could plausibly be used to elicit placebo effects in 169 clinical and laboratory-based studies involving non-malignant pain, drawn from 7 systematic reviews. In a validation exercise we surveyed 33 leading placebo researchers (M=12 years' research experience, SD=9.8), who were asked to comment on and add to the draft taxonomy derived from the literature.

Results. The final taxonomy defines 30 procedures that may contribute to placebo effects in clinical and experimental research, proposes 60 possible clinical applications, and classifies procedures into 5 domains: the *Patient's Characteristics and Belief* (5 procedures and 11 clinical applications); the *Practitioner's Characteristics and Beliefs* (2 procedures and 4 clinical applications); the *Healthcare Setting* (8 procedures and 13 clinical applications); *Treatment Characteristics* (8 procedures and 14 clinical applications); and the *Patient-Practitioner Interaction* (7 procedures and 18 clinical applications).

Conclusions. The taxonomy provides a preliminary and novel tool with potential to guide translational research aiming to harness placebo effects for patient benefit in practice.

Keywords: placebos; placebo effect; nocebo effect; translational research; review; classification

For peer review only - http://bmjopen.bmj.com/site/about/guidelines.xhtml

Article Summary

Strengths and Limitations of this Study

- This is a novel attempt to use existing studies to conceptualise the factors that might contribute to placebo effects and the associated procedures that could be simply and ethically adapted for clinical practice, subject to further testing .
- We drew on both clinical trials and laboratory-based studies of placebo effects, in order to generate a more comprehensive list of factors that might contribute to placebo effects than would be possible by relying on just one literature.
- A systematic approach to data synthesis, based on qualitative research methods, was used to identify and classify procedures that might contribute to placebo effects in clinical trials.
- The development of the taxonomy did not incorporate very recent placebo trials or studies andthe selection of reviews used to determine which original studies to include in the development process was somewhat arbitrary.
- Our taxonomy is presented not as an exhaustive compilation of current methods used in placebo research but as a detailed and systematic guide for future research, which can in turn further refine the taxonomy.

For peer review only

For peer review only - http://bmjopen.bmj.com/site/about/guidelines.xhtml

Introduction

There is compelling evidence that factors other than the so-called active components of treatment can have clinically meaningful effects on symptoms, particularly non-malignant pain [1-4]. Such 'placebo effects' can be defined as the physiological and/or psychological changes that result from the meaning derived by a person in a health care setting [5 6]. From a learning perspective, expectations play a key role in placebo effects.[7] These effects may be as large as treatment effects [8] and occur throughout medicine, especially when doctors and patients interact with each other. They are not routinely deliberately harnessed for patient benefit in clinical practice [9], possibly because doctors often assume they must deceive patients in order to elicit placebo effects [10 11]. However, this assumption is mistaken because it is not necessary to prescribe placebos in order to elicit placebo effects. For example, the overall analgesic effect of an opioid derives not only from its specific pharmaceutical actions but also from its psychological components, i.e. the *expectations* and *meaning* that the patient derives when consulting the doctor and taking the medicine[12 13]. The same is true for other types of intervention including physical, surgical, and psychotherapies. One approach that has received initial support is for doctors to use positive suggestion to enhance patients' expectations of benefit [4]. Furthermore, preliminary evidence suggests that openly prescribing placebos might elicit clinically meaningful placebo effects in IBS and depression [14 15] although this approach entails its own set of ethical challenges[16 17].

Placebo researchers have both begun and called for more translational research in this field [11 18-20]. Such work has thus far typically focused on ethical considerations and narrative approaches to drawing out implications for clinical practice from the placebo literature. We suggest a systematic approach to translational research might be helpful. Many techniques and procedures contribute to placebo effects and could potentially be simply and ethically adapted for clinical practice, subject to further testing in practice settings [21]. In order to identify and describe such techniques, and thus

For peer review only - http://bmjopen.bmj.com/site/about/guidelines.xhtml

provide some direction for future research, we reviewed experimental and clinical studies of placebo effects in non-malignant pain. We focused on non-malignant pain because it can be difficult to manage (particularly with current concerns about opioids [22]), the mechanisms underpinning placebo analgesia are reasonably well understood [23], laboratory-based experimental studies often focus on placebo analgesia, and patients with pain have been shown to display substantial and clinically significant placebo effects [1]. The aim of this project was to facilitate translational research by producing a taxonomy of techniques that may contribute to observed placebo effects in research settings and could be studied in future as potential approaches to augmenting placebo enhancement of analgesia in clinical practice.

Methods

Literature Search

We selected seven systematic reviews of different aspects of the placebo literature, chosen from recent reviews available at the time (2012) and based on expert opinion (within the research team) to enable the extraction of information on placebo procedures from a broad range of settings - comprehensive reviews [24-26], reviews of placebo effects in clinical populations [2 27] and reviews of laboratory-based experimental placebo studies [28 29]. The key consideration was that together this collection of reviews should cite a diverse set of studies likely to be using diverse methods to directly (e.g. placebo mechanisms studies) or indirectly (e.g. clinical trials with placebo controls) study placebo effects. After removing duplicates and ineligible studies (see Figure 1), 169 studies were used to develop the taxonomy (for list of included studies see Supplementary Digital Content).

For peer review only

Studies were eligible for inclusion if they: reported original research in which some participants received a placebo intervention, were published since 1983, published in English language, reported a non-malignant pain outcome. Studies were excluded if they: were published before 1983 (because (a) means of generating context-dependent placebo effects may be sensitive to social and cultural changes over time, e.g. patient preferences for particular communication styles and thus their effectiveness in modifying expectations may have changed over time and (b) this yielded a manageable number of papers to analyse which had been published during the 30 years preceding this analysis); or examined any type of psychotherapeutic interventions (because it is difficult to disentangle the active components of psychotherapy from the effect of the meaning of the intervention [30]).

Data Extraction and Synthesis

Descriptions of all events that occurred in the placebo groups during each of the 169 studies (e.g. medical, administrative, and ethical procedures) were extracted into a piloted form by one author and checked by a second. These events were reviewed for duplication and overlap. This resulted in an initial list of 43 procedures that might contribute to placebo effects (e.g. informed consent processes, taking placebo pills, conditioning protocols). Study authors were not contacted for further information about method used.

To synthesise the data and develop our taxonomy we used systematic and rigorous methods derived from qualitative research. We began with a deductive analysis, which aimed to categorise the

For peer review only - http://bmjopen.bmj.com/site/about/guidelines.xhtml

For peer review only

procedures in a way that is intuitively appealing, accessible, and clinically relevant by sorting them into five previously-identified contextual domains of healthcare: patients' characteristics/beliefs, practitioners' characteristics/beliefs, practitioner-patient relationship, superficial treatment characteristics, and the healthcare environment [31]. Two authors (BC, FB) performed the initial categorisation which was then reviewed in detail by three other authors (GL, HE, AG). We then engaged in a constant comparative analysis, a technique that originates in grounded theory [32]. The aim of this part of the analysis was to consolidate the list of procedures and ensure that we only retained those that were distinct from each other. Procedures and examples of their use were all systematically compared to each other; similar procedures were then merged and all procedures were classified into one of the five domains. Two authors (BC, FB) led this work and presented initial findings to the rest of the team for discussion. All authors discussed and agreed on which procedures to merge, which to retain, and how to classify them. During this process, the definitions of the five domains were iteratively modified in order to reduce ambiguity over which procedures should be classified into which domain. . This resulted in a more parsimonious list of 29 procedures classified by domain. All authors discussed and agreed on the final classification of these procedures. These 29 procedures were then critically examined to ensure they were theoretically plausible means of producing placebo effects. We focused on three core psychological mechanisms [25 33-36]: response expectancy [37]; conditioning and social learning [7]; and affect, including motivation and anxiety-reduction [24 38]. However, we acknowledge that these mechanisms are difficult to tease apart [39] and that alternative mechanisms have been proposed [6] and so we erred on the side of inclusivity. Neurobiological mechanisms of placebo analgesia have been described [23 40] but a detailed consideration of how these might apply to the procedures in the taxonomy would be highly speculative and was beyond the scope of this project (for discussion of clinical applications of the neuroscience of placebo effects see [41]). Four authors (FB, BC, AG, GL) reviewed all procedures and considered the extent to which each procedure could plausibly produce placebo effects via one or more of the three core psychological mechanisms. Initial findings were shared with the remaining

For peer review only - http://bmjopen.bmj.com/site/about/guidelines.xhtml

authors and consensus was reached through discussion. Four procedures deemed very unlikely to produce placebo effects (Conveying a Neutral Therapeutic Message; Randomisation; Blinding; Deception) were excluded, leaving 25 procedures that might plausibly contribute to placebo effects. The multidisciplinary team of authors then generated possible clinical applications of each of these 25 procedures.

Validating the Taxonomy

To ensure our taxonomy was comprehensive we surveyed leading placebo researchers (authors of major publications on placebo effects, attendees at an international symposium on placebo effects, and GPs with an interest in placebo effects). These researchers were identified from the systematic reviews and their references used to develop the taxonomy; the list of attendees at Beyond The Placebo: Biomedical Clinical and Philosophical Aspects of the Placebo Effect, held in Ascona Switzerland, August 2012; and GPs within the National Institute for Health Research School for Primary Care Research. Ethical approval for the survey was obtained from the host institution (reference: 4741). Completed electronic surveys including informed consent were received from 33 researchers (52% response rate) experienced in placebo research (M=12 years' experience, SD=9.8). Respondents were shown our draft taxonomy and asked whether, for each domain, they knew of any other procedures that could elicit placebo responses. The proportion answering yes ranged from 22% (Healthcare Setting domain) to 50% (Superficial Treatment Characteristics domain). Respondents suggested 85 additions which were screened against existing procedures and for theoretical plausibility: 80 of the suggested additions were extra details or suggested clinical applications of existing procedures; five were new and distinct plausible procedures that were added to the taxonomy, giving a final total of 30 procedures.

Because of our orientation to clinical applications, we have chosen to use clinically-oriented terminology throughout the taxonomy. However, it is important to note that when used in relation

For peer review only - http://bmjopen.bmj.com/site/about/guidelines.xhtml

to procedures identified from the literature these terms also relate to the experimental equivalent, such that "patient" also refers to subject/participant, "practitioner" also refers to experimenter, and "intervention" also refers to experimental condition.

Analysis

The use of each of the 30 procedures in the taxonomy was assessed across all 169 studies in the review. Two authors independently rated the presence of each procedure in each study (Kappa = 0.93, discrepancies were resolved through discussion).

Results

The taxonomy defines 30 procedures that may contribute to placebo effects observed in clinical and experimental research, and classifies them into 5 domains. Table 1 presents the main taxonomy, listing and defining all 30 procedures within 5 domains. Table 2 suggests clinical applications of each procedure. Table 3 shows the frequency of use of each procedure in clinical and experimental studies, and is intended as both an approximate guide to whether the procedures derived primarily from one or other literature and as a means to highlight those procedures that are very common and very rare in the literature. Below we describe the procedures within each domain in turn.

For peer review only

199 Table 1. Taxonomy of Procedures Which Could Plausibly Elicit Placebo Effects in Non-Malignant Pain

Procedure Derived from Literature	Definition and Use in Research Studies
**The Patient's Beliefs and Characteristics**	
1. Select Patients Based on Treatment History.	Screen and select patients (or subgroups) against inclusion criteria related to issues such as medical/treatment history, e.g. naive to intervention being tested (not just contraindications).
2. Create Positive Expectancy.	Deliberately and explicitly suggest to patients that the intervention will be effective for them (not as part of informed consent process).
3. Reduce Negative Expectancy. [a]	The potentially negative or harmful procedures and characteristics of the treatment are deliberately minimised in information for patients.
4. Convey a Positive Therapeutic Message through Informed Consent Procedures.	Convey (verbally or in writing) a positive therapeutic message through the content of informed consent. The message might be explicit (e.g. "this intervention is usually effective in most people") or implicit (e.g. "this treatment is an antihypertensive").
5. Harness Socio-cultural Context. [a]	Tailor the intervention according to the patient's social and cultural context and history.
**The Practitioner's Beliefs and Characteristics**	
6. Practitioner Expectancy.	The person delivering the treatment expects it to be effective for the patient.
7. Practitioner's Personal Characteristics.	The practitioner's personal and/or professional characteristics (e.g. status) are modified (through selecting practitioners with different characteristics) and/or emphasised to patients.
**The Healthcare Setting**	
8. Active Recruitment.	Actively seek out and recruit patients (e.g. advertising for specific types of patients, writing personally to individual eligible patients identified through medical records).
9. Active Retention.	Make patients feel valued by attempting to keep them in a study (e.g. contact patients if they miss an appointment, incentivise attendance through monetary or non-monetary gifts).
10. Follow-up.	Assess patients after the intervention/experiment to assess long-term maintenance or changes in effects over at least 6 months.
11. Follow a Standardised Protocol.	The intervention is delivered according to a set, scientifically-derived, protocol, lending credibility to the intervention (and is therefore not individualised for each patient).
12. Ethical Oversight.	Study practices and procedures are explicitly regulated and monitored by an institutional ethics committee, lending credibility to the intervention.
13. Participating in Research.	Patients know that they are part of research and contributing to the furthering of human knowledge and/or improvement of healthcare for future patients.
14. Symptom Monitoring.	Monitor patients' symptoms using self-report measures, practitioner assessment, or objective measures

For peer review only - http://bmjopen.bmj.com/site/about/guidelines.xhtml

	repeated over time at least twice; patients are aware of the resulting measurements.
15. Enhanced Environment. [a]	The physical and interpersonal environment where the intervention is delivered is deliberately enhanced.
**Treatment Characteristics**	
16. Sham Intervention – Medication.	An inert substance is administered which is manufactured to appear identical to an active medication (e.g. sugar pill, saline IV, topical agent).
17. Sham Interventions – Physical.	A sham physical intervention is administered which is designed to appear identical to the genuine intervention (e.g. de-activated TENS, non-penetrative acupuncture needles at non-acupuncture points).
18. Sham Interventions – Attention Only.	Patients receive study-specific attention in terms of numbers of visits and time spent with study staff but no additional intervention.
19. Ineffective substances. [a]	Products unlikely to be effective or not indicated are administered (e.g. vitamins in the absence of vitamin deficiency).
20. Use Salient Side-Effects.	Potential side-effects are highlighted such that the patient can interpret them as evidence of a potent intervention.
21. Matched Treatments.	To secure blinding, placebo/sham treatments are matched to ‘real’ treatments (e.g. on mode of administration, dosage, frequency of administration, visual appearance, taste, smell, individual titration procedures).
22. Maximised Treatment Procedures.	The procedures and characteristics of the treatment are exaggerated, e.g. through high dose, use of colour, high frequency, large pill size, lengthy duration of intervention, ritualistic administration.
23. Conditioning.	A desired response (e.g. pain relief) is paired with an intervention stimulus (e.g. placebo cream) so that the patient associates the response with the stimulus.
**The Patient-Practitioner Interaction**	
24. The Process of Informed Consent.	The patient’s formal written and/or verbal informed consent is discussed and obtained.
25. Detailed History.	A detailed personal and/or medical and/or psychosocial history is obtained from the patient.
26. Diagnosis/tests.	Additional tests, examinations, or confirmatory diagnostic procedures are undertaken to establish eligibility for the study.
27. Care.	The practitioner deliberately engages the patient with warmth, compassion and empathy.
28. Patient-Centred Communication. [a]	The practitioner adopts a style of consultation that they consider to be appropriate for a particular patient.
29. Extra Attention.	The patient receives extra attention from being in the study, for example is seen more frequently or for longer than usual.
30. Continuity of Care.	Efforts are made for the same practitioner to see the same patient at each contact.

[a] procedures added following survey of researchers.

For peer review only - http://bmjopen.bmj.com/site/about/guidelines.xhtml

Table 2. Suggested Potential Clinical Applications of Procedures to Elicit Placebo Effects in Non-Malignant Pain, Subject to Further Research

Procedure	Suggested Clinical Applications
**The Patient's Beliefs and Characteristics**	
1. Select Patients Based on Treatment History.	Stop prescribing interventions of a type that a patient has previously not responded to (e.g. tablets); instead, prescribe a different, new, type of treatment (e.g. psychological therapy).
2. Create Positive Expectancy.	Tell the patient the intervention is likely to be effective.<br>Elicit patients' treatment and illness beliefs and expectations, and dispel any misconceptions.<br>Empower patients to self-care.
3. Reduce Negative Expectancy.	Limit emphasis on major potential side effects, and describe how uncommon they are.<br>Hide cessation of analgesia administration (e.g. as in Benedetti [42]), after obtaining advanced consent and ensuring patients are aware they can request additional analgesia if needed.
4. Convey a Positive Therapeutic Message through Informed Consent Procedures.	Provide written and/or verbal information that conveys a positive therapeutic message about treatment.<br>Provide clear rationale for treatment.<br>Provide patient testimonials and supporting literature/media.
5. Harness Socio-cultural Context.	Elicit patients' culturally embedded treatment and illness beliefs, preferences and expectations, dispelling any potentially harmful misconceptions.<br>Involve significant others in care.
**The Practitioner's Beliefs and Characteristics**	
6. Practitioner Expectancy.	Only prescribe a treatment to patients when the practitioner expects it will be effective; communicate that expectation to patients.
7. Practitioner's Personal Characteristics.	Honour patient preferences for particular practitioners.<br>Use indicators of expertise/high status in offices, in correspondence, and when referring to other practitioners.<br>Ensure the patient is seen by a practitioner whose views/values are congruent with the patient's.
**The Healthcare Setting**	
8. Active Recruitment.	Actively seek out patients and invite them to attend clinic regarding a particular intervention (as opposed to waiting for patients to present).
9. Active Retention.	Personally contact patients if they miss an appointment.<br>Use incentives to encourage patients to keep appointments.
10. Follow-up.	Routinely invite patients to book a follow up appointment after an intervention has finished and prior to repeat prescription.<br>Encourage the patient to take responsibility for and self-manage their condition following an intervention.
11. Follow a Standardised Protocol.	Use patient-friendly treatment protocols and share with patients where they fit in that protocol.

For peer review only - http://bmjopen.bmj.com/site/about/guidelines.xhtml

12. Ethical Oversight.	Ensure that patients understand that their treatment protocol is sanctioned by a higher authority e.g. NICE.
13. Participating in Research.	Inform patients that all outcomes and practitioner performance is audited and can contribute to improved knowledge and treatment for future patients.
14. Symptom Monitoring.	Ask patients to monitor their symptoms regularly, for example using email, phone apps, web-based systems, paper forms.<br>Assess treatment outcome.<br>Give patients feedback on symptom improvements following monitoring.
15. Enhanced Environment.	Ensure that the environment is professional, pleasant and peaceful.<br>Employ friendly and helpful support staff.
**Treatment Characteristics**	
16. Sham Intervention – Medication.	Openly prescribe sham medication.<br>With advanced prior consent, prescribe sham medication.
17. Sham Interventions – Physical.	Openly prescribe sham physical treatments.<br>With advanced prior consent, prescribe sham physical treatments.
18. Sham Interventions – Attention Only.	Increase frequency and duration of consultations.
19. Ineffective substances.	Prescribe substances that are likely not to cause harm but not clearly indicated or substances unlikely to be effective e.g. simple linctus.
20. Use Side-Effects.	Tell patients about side effects associated with positive clinical outcome.
21. Matched treatments.	Design appearance of prescribed substance (e.g. colour, packaging, taste) to match known effective treatments.
22. Maximised treatment procedures.	Within safety limits prescribe higher dose/higher frequency/larger pill.<br>Use different colour treatments.<br>Instigate ritualistic procedures patients can perform when taking medicines.<br>Maximise adherence to treatment through education, easy follow up appointments, easy repeat prescription arrangements etc.
23. Conditioning.	Prescribe highest tolerated dose first, then titrate downwards.<br>With consent, begin with active intervention, pair with a seemingly identical placebo then substitute for placebo alone.(e.g. as in Sandler[43])
**The Patient-Practitioner Interaction**	
24. The Process of Informed Consent.	Actively seek patient consent.<br>Provide treatment options and encourage the patient to choose from these options if they so desire.
25. Detailed History.	Take a detailed medical and psychosocial history/update.<br>Ensure the patient feels listened to, e.g. through non-verbal communication and/or capturing information.<br>Ask questions about the meaning of symptoms.
26. Diagnosis/tests.	Provide a definitive/confident diagnosis.<br>Examine the patient fully.
27. Care.	Allow patient adequate time to tell their story and listen to them.<br>Validate the patient's concerns.<br>Use non-verbal techniques to convey empathy, compassion, warmth.<br>Use touch judiciously.

For peer review only - http://bmjopen.bmj.com/site/about/guidelines.xhtml

28.	Patient-Centred Communication.	Individualise consultation style according to a patient's preference e.g. collaborative vs authoritative.<br>Engage in collaborative decision-making with the patient.<br>Develop shared treatment goals that you and the patient agree on.
29.	Extra Attention.	Give extra attention to or show more interest in a patient by seeing them more frequently, having longer consultations or visiting at home.<br>Do not rush the patient.
30.	Continuity of Care.	Ensure patient is cared for by the same practitioner.<br>Read records before consultation.

Note. Suggestions for clinical applications pending research into effectiveness and ethical acceptability in clinical settings.

For peer review only

For peer review only - http://bmjopen.bmj.com/site/about/guidelines.xhtml

Table 3. Use of Procedures in Placebo Groups of Clinical and Experimental Studies

Procedure	% of studies that used each procedure: Experimental (n=58)	Clinical (n=111)
**The Patient's Beliefs and Characteristics**		
1. Select Intervention Based on Patient's Treatment History.	55%	75%
2. Create Positive Expectancy.	76%	5%
3. Reduce Negative Expectancy.	3%	0%
4. Convey a Positive Therapeutic Message through Informed Consent Procedures	43%	1%
5. Harness Socio-cultural Context.	0%	0%
**The Practitioner's Beliefs and Characteristics**		
6. Practitioner Expectancy.	0%	1%
7. Practitioner's Personal Characteristics.	9%	0%
**The Healthcare Setting**		
8. Active Recruitment.	14%	16%
9. Active Retention.	3%	2%
10. Follow-up.	2%	16%
11. Follow a Standardised Protocol.	85%	63%
12. Ethical Oversight.	78%	69%
13. Participating in Research.	86%	84%
14. Symptom Monitoring.	95%	89%
15. Enhanced Environment.	5%	0%
**Treatment Characteristics**		
16. Sham Intervention – Medication.	71%	55%
17. Sham Interventions – Physical.	33%	41%
18. Sham Interventions – Attention Only.	2%	5%
19. Ineffective substances.	0%	1%
20. Use Side-Effects.	0%	1%
21. Matched treatments.	40%	82%
22. Maximised treatment procedures.	22%	3%
23. Conditioning.	41%	0%
**The Patient-Practitioner Interaction**		
24. The Process of Informed Consent.	88%	77%
25. Detailed History.	19%	33%
26. Diagnosis/tests.	36%	41%
27. Care.	0%	1%
28. Patient-Centred Communication.	0%	0%
29. Extra Attention.	2%	63%
30. Continuity of Care.	7%	14%

For peer review only - http://bmjopen.bmj.com/site/about/guidelines.xhtml

The Patient's Characteristics and Beliefs

The taxonomy specifies five procedures that act directly on the patient's characteristics and/or beliefs in ways that might contribute to placebo effects. Procedure 1 involves selecting patients who are most likely to benefit from an intervention based on their history of similar treatments (where similarity is construed broadly at multiple levels, including appearance, modality, style, and pharmacology). For example, one might select those patients who have not experienced disappointing results from a similar intervention in the past (as the latter group might have learned to expect the intervention to fail). This procedure was commonly used by clinical trials and (to a lesser degree) experimental studies.

Procedures 2 (create positive expectancy), 3 (reduce negative expectancy), and 4 (convey a positive therapeutic message), all involve communicating with patients to encourage them to expect beneficial effects of treatment or not to expect side-effects. The majority of experimental studies in our review explicitly encouraged patients to expect treatment benefits, while very few clinical studies explicitly targeted patients' expectations and hardly any studies at all attempted to minimise patients' expectations of side-effects. Procedure 5 involves tailoring the intervention to the patient's socio-cultural context. This approach emerged from the expert feedback and while it seems plausible and ethical to translate into clinical practice, it was not used by any of the reviewed studies.

The procedures in the patients' beliefs and characteristics domain are thought to contribute to placebo effects primarily through altering patients' response expectancy. Selecting patients based on treatment history and tailoring to socio-cultural context are also predicated on learning mechanisms, i.e., learned associations between treatment outcome and treatment properties.

For peer review only - http://bmjopen.bmj.com/site/about/guidelines.xhtml

There is some evidence that clinicians can give verbal suggestions to alter patients' expectations in practice and in doing so to reduce patients' pain, particularly acute procedural pain [4 44]. As part of work to implement these procedures more widely in practice it would be important to investigate how to secure ethically valid consent for treatment. For example, clinicians might want to encourage realistically positive patient expectations while providing information about possible harms without inducing the negative expectations that could trigger nocebo effects [45 46].

The Practitioner's Characteristics and Beliefs

The two procedures in this domain are about using or modifying health care practitioners' characteristics and/or beliefs. Procedure 6 requires a practitioner to expect a treatment to benefit the patient. This might contribute to observed placebo effects in patients by influencing a practitioner's communication about the treatment and hence a patient's response expectations and/or affective response to the consultation. Only 1% of clinical studies and no experimental studies reported modifying practitioners' expectations. This procedure has received little attention in the placebo literature but clinical research in musculoskeletal settings suggests practitioners' outcome expectations can predict patients' pain outcomes [47]. One way to implement this procedure in practice would be for practitioners to communicate explicitly that they believe a treatment is effective, an approach which clearly overlaps with communication interventions designed to help doctors encourage patients to have positive expectations. Implementing Procedure 6 also depends on practitioners having relevant high quality evidence readily available and accessible and understanding this evidence as it applies to the patient.

A small proportion of studies (9% of experimental studies and no clinical studies) emphasised a practitioner's status or other characteristics (Procedure 7). For some patients a high status practitioner might elicit more confidence in the treatment (and thus higher expectations) and/or a

For peer review only - http://bmjopen.bmj.com/site/about/guidelines.xhtml

more positive affective response to the consultation [48]. Some aspects of this procedure are already part of clinical practice, for example the routine display of medical certificates in doctors' offices; others are inherent in the tools of the doctor, such as the symbolic properties of the stethoscope [49]. However, there is likely to be scope for testing their effects and augmenting their use if appropriate.

The Healthcare Setting

Procedures 8 and 9 relate to the active efforts made in studies to recruit and retain patients respectively. Clinical and experimental studies both reportedly used these procedures sparingly (<20% for active recruitment and <5% for active retention). Such efforts may make patients feel valued and could be implemented in practice through the use of personalised communications from practitioners to encourage attendance at appointments.

Three of the eight procedures in this domain were used by over half of clinical and experimental studies and relate to basic structural features of research: following a protocol, ethical oversight, and participating in research (procedures 11 to 13). They are thought to impact patients' expectations, by emphasising the legitimacy of the intervention that is being provided and the importance of the patient's contribution to a bigger project, i.e. generating knowledge. Translating these procedures into practice could involve, for example, clinicians explicitly talking with patients about official guidance and treatment protocols that they are following.

Symptom monitoring (procedure 14) was commonly used in both clinical and experimental studies. This could be implemented in practice for example through repeatedly using patient reported outcome measures (see [50]) and might contribute to placebo effects through learning mechanisms (e.g. regular symptom monitoring acts as feedback to motivate health behaviours and/or modify

For peer review only - http://bmjopen.bmj.com/site/about/guidelines.xhtml

patients' goals). Alternatively, the mere act of asking a patient to monitor their symptoms could convey an expectation of treatment benefit, altering the meaning of a clinical interaction for the patient. Traditionally such effects of the act of measurement are dismissed as Hawthorne effects but they may also be encompassed in broader definitions of placebo effects as meaning effects [51] and could thus enhance effects in clinical practice despite being considered a nuisance in clinical research.

Very few placebo studies (5% of experimental and no clinical studies) reported enhancing the physical or interpersonal environment (procedure 15). There is a separate and distinct literature on environment modifications in health settings that might be usefully integrated with the placebo literature when developing clinical applications in this area and modelling mechanisms of action [52 53].

Treatment Characteristics

Eight procedures in the taxonomy involve modifying the characteristics of a treatment. Three involve prescribing sham interventions (sham medication – procedure 16, sham physical interventions – procedure 17, and extra attention – procedure 18) while a fourth involves prescribing a substance unlikely to be effective for the symptom in question (procedure 19). These four procedures represent variations in control conditions used in research and were frequently used by both clinical and experimental studies (with the exception of extra attention which was only used by 2-5% of studies). Such controls are thought to operate primarily via expectations, while affective pathways may also be important when extra attention from trial personnel/medical staff is involved. Of all the procedures in the taxonomy these four that represent control conditions come closest to the traditional notion of how placebos could be applied in practice. Given ethical concerns around deceptive prescribing we suggest that translational research might continue to focus on openly prescribing sham interventions including placebo pills (as in [14 15]). Other options should not

For peer review only - http://bmjopen.bmj.com/site/about/guidelines.xhtml

be dismissed entirely though: advanced consent and even waiving consent are acceptable to some patients and so it is vital for translational research to continue exploring patients', practitioners', and other stakeholders' views on the acceptability and ethics of diverse ways of prescribing placebos [54-57].

Three procedures in this domain modify the superficial (non-pharmacological or non-defining) characteristics of treatments. Procedure 20 is to highlight treatment side-effects to patients in order to encourage patients to see the treatment as potent; this procedure was very rare, used by only 1% of clinical studies. Procedure 21 was much more commonly used and involves matching the appearance of real and control treatments (used by 40% of experimental and 82% of clinical studies), in order to maintain patient blinding. This could be translated into clinical practice by designing the appearance of interventions to match patients' beliefs about what effective interventions look like. Procedure 22 involves maximising or exaggerating the superficial characteristics of treatment in order to generate larger placebo effects for example by using colour, large pill size, or ritualistic administration of medicines, manipulations which could alter the meaning of a treatment for a patient and/or enhance their expectations. 22% of experimental studies reported using this procedure and one way to translate it into practice would be to create (and test) ritualistic procedures for patients to engage in when taking medicines.

The final procedure in this domain – procedure 23, conditioning to generate placebo effects - was used commonly and exclusively by experimental studies (41%). Conditioning protocols generate placebo effects through learning mechanisms and perhaps could be implemented in practice to reduce pharmaceutical dosages, as was achieved in a pilot study in children with attention deficit disorder [43].

For peer review only - http://bmjopen.bmj.com/site/about/guidelines.xhtml

The Patient-Practitioner Interaction

The *Patient-Practitioner Interaction* domain incorporates seven procedures related to the interpersonal relationship or interactions between a patient and their health care practitioner. These procedures are thought to operate primarily through affective mechanisms such as reduced anxiety after telling one's story and being listened to with empathy and acknowledged, although more cognitive pathways via expectations are also plausible[58]. Three procedures are about specific processes that can occur during consultations – obtaining informed consent (procedure 24), taking a detailed history (procedure 25), and performing additional diagnoses or tests (procedure 26). Arguably these procedures indicate to the patient that the practitioner respects them, is interested in their perspective, and is thorough in their diagnosis. They occur in both clinical and experimental research settings and could be relatively directly translated into practice or optimised if already used.

Two procedures are about the way in which the practitioner engages with the patient: communicating care (procedure 27), and patient-centred communication (procedure 28). These procedures were surprisingly very rarely described in the studies included in our review, although recently the nocebo effects of *not* validating a patient's experiences have been shown to be particularly potent [59]. There is of course a distinct and large literature on doctor-patient communication and fruitful dialogue is beginning to bridge these fields [60].

The final two procedures in this domain refer to more structural aspects of consultations: extra attention (procedure 29, i.e. longer or more frequent appointments) and continuity of care (procedure 30). 63% of clinical studies used extra attention while a small proportion of clinical (14%) and experimental (7%) studies reported providing continuity of care. Directly implementing these procedures in practice might be challenging given ever increasing constraints on healthcare resources and drives to reduce cost.

For peer review only - http://bmjopen.bmj.com/site/about/guidelines.xhtml

Discussion

The taxonomy names and describes 30 procedures that may contribute to placebo effects in experimental and clinical studies and classifies them into five domains. It includes 60 theoretically plausible clinical applications, subject to further research on their effectiveness and ethical acceptability in practice. Some of the clinical applications derived from the placebo literature have already been investigated in their own right under other auspices, highlighting the need for the burgeoning translational science of placebo effects to be broad-ranging and interdisciplinary.

We have used rigorous systematic review and qualitative analytic methods complemented by a survey to develop the taxonomy. Investigators often combine multiple techniques in any one 'placebo' (e.g. Create Positive Expectancy + Detailed History + Symptom Monitoring) making it beyond the scope of this project to unpack the effectiveness of individual techniques. Procedures did not always fit neatly into single domains. For example, "Screen for Treatment History" was used to select patients for studies of specific treatments (and was thus placed in the Patient's Beliefs and Characteristics domain), but its clinical application involves selecting a treatment for a specific patient and so could be considered a Treatment Characteristic. Conceptually we would expect interactions between these domains; for example, some procedures categorised in other domains probably operate through causal pathways involving patients' beliefs as proximal determinants of placebo effects [61]. We feel the benefits of having a hierarchical structure (modifiable as the taxonomy is refined with use) outweigh the difficulties inherent in classification. We could have used many published reviews of placebo studies in non-malignant pain to identify original studies to review. Selecting seven such reviews means not using others, thus we might have missed original studies that would have suggested additional procedures. Surveying leading researchers and incorporating their suggestions somewhat mitigates this limitation. Our sample of researchers was

For peer review only - http://bmjopen.bmj.com/site/about/guidelines.xhtml

intended to be purposive, in that we wanted to obtain the views of leading researchers in the field. By using multiple means of identifying such individuals internationally we feel we have achieved this. The reviews that we selected as the source of our papers and the papers themselves are now somewhat old examples of the literature; and our choice to exclude papers published before 1983 was arguably somewhat arbitrary. Future work should review very recent papers and iteratively improve the taxonomy accordingly.

This review extends previous work by Di Blasi et al. [31], building on their five domains to systematically develop a detailed taxonomy. We provide a new overarching framework that avoids the controversial and limited distinction between pure and impure placebos [62 63] and integrates ideas from the rich clinical and experimental literatures on placebo effects in non-malignant pain. Many of the components we have identified are likely to be important in other placebo-responsive conditions including depression [64], irritable bowel syndrome [65 66], and insomnia [67]. This taxonomy can guide two important and related applied research agendas: 1) to understand the components of placebo effects in clinical settings [48 68-71] and 2) to ethically harness evidence-based placebo effects to benefit patients [14 15 72]. We hope future studies might draw on the taxonomy to fully describe their methods and develop new applications, thus facilitating future systematic reviews and the development of a systematic and theory driven cumulative evidence-base in this complex field.

The taxonomy identifies and classifies procedures that may contribute to placebo effects in clinical trials and experiments, providing an overarching framework for individual components. However, we do not suggest that every technique in this taxonomy will produce a placebo effect in every patient and we do not know from this project which techniques are more effective or how they might be combined to form ethically acceptable and effective complex interventions. This taxonomy

For peer review only - http://bmjopen.bmj.com/site/about/guidelines.xhtml

provides the first attempt at a necessary conceptual tool to facilitate future research on these questions. For example, systematic reviews could use the taxonomy to code procedures in original studies, using this information in meta-regression analysis to examine the contribution of different procedures to placebo effects [73]. New clinical trials and experiments could extend existing work by systematically examining and comparing the effects and ethical acceptability of different procedures in the taxonomy, building a cumulative evidence-base that has real pragmatic applicability to clinical practice. Some of the suggested clinical applications have been investigated more extensively in other literatures, in particular doctor-patient communication and the healthcare environment. This emphasises the need for a multi-disciplinary approach to the translation of placebo research into practice. One fruitful way forward would be to draw on placebo theories to develop and test more mechanistic models of complex interventions intended to alter the context of healthcare encounters.

Placebo recipients in clinical trials and experiments are exposed to a large number and variety of procedures, many of which might contribute to placebo effects. Researchers seeking to develop a translational science of placebo effects are thus faced with myriad possibilities. We have systematically identified and defined these procedures, classified them into five domains, and suggested possible clinical applications. The resulting taxonomy is presented as a preliminary but detailed and systematic guide for future research, which should in turn further refine the taxonomy. Ultimately we hope to better conceptualise investigations of clinical applications of placebo effects in order to maximise opportunities for patient benefit.

For peer review only

For peer review only - http://bmjopen.bmj.com/site/about/guidelines.xhtml

Funding

The project “Creating a Taxonomy to Harness the Placebo effect in UK primary care” was funded by the National Institute of Health Research (NIHR) School for Primary Care Research (SPCR) (project number 161). This paper presents independent research funded by the National Institute of Health Research (NIHR). The views expressed are those of the author(s) and not necessarily those of the NHS, the NIHR or the Department of Health. Additional funding for BC was provided by Solent NHS Trust. The funders had no role in design and conduct of the study; collection, management, analysis, and interpretation of the data; preparation, review, or approval of the manuscript; and decision to submit the manuscript for publication.

Acknowledgements

We would like to acknowledge the contribution of all of the researchers who shared their views in the survey, including Przemyslaw Babel PhD, Luana Colloca MD PhD, Professor Michael Doherty, Vanda Faria PhD, Professor Magne Arve Flaten PhD, Sarah Goldingay PhD, John Hughes PhD, Professor Dr Robert Juette, Irving Kirsch PhD, Karin Meissner PD Dr. med. Habil., Daniel E Moerman PhD, Meike Shedden Mora PhD, Donald D. Price PhD, Professor Dr Dr Harald Walach. We thank Professor Ted Kaptchuk for comments on an earlier draft of this manuscript.

Competing Interests

All authors have completed the ICMJE uniform disclosure form at http://www.icmje.org/coi_disclosure.pdf and declare: no support from any organisation for the submitted work; no financial relationships with any organisations that might have an interest in the

For peer review only - http://bmjopen.bmj.com/site/about/guidelines.xhtml

submitted work in the previous three years, no other relationships or activities that could appear to have influenced the submitted work.

Exclusive Licence

The Corresponding Author has the right to grant on behalf of all authors and does grant on behalf of all authors, a worldwide licence (http://www.bmj.com/sites/default/files/BMJ%20Author%20Licence%20March%202013.doc) to the Publishers and its licensees in perpetuity, in all forms, formats and media (whether known now or created in the future), to i) publish, reproduce, distribute, display and store the Contribution, ii) translate the Contribution into other languages, create adaptations, reprints, include within collections and create summaries, extracts and/or, abstracts of the Contribution and convert or allow conversion into any format including without limitation audio, iii) create any other derivative work(s) based in whole or part on the on the Contribution, iv) to exploit all subsidiary rights to exploit all subsidiary rights that currently exist or as may exist in the future in the Contribution, v) the inclusion of electronic links from the Contribution to third party material where-ever it may be located; and, vi) licence any third party to do any or all of the above.

Contributorship Statement

FB designed and led the study, drafted the manuscript, and is guarantor. FB, GL, AWAG, HE, and PL secured funding for the project. FB designed the study with input and revisions from GL, BC, AWAG, HE, and PL. BC led data collection and analysis with additional data collection and analysis by MH and DS. All authors contributed to data interpretation. FB drafted the manuscript and all authors revised it for important intellectual content. All authors had full access to all of the data in the study and can take responsibility for the integrity of the data and the accuracy of the data analysis.

Data Sharing Statement

No unpublished data available.

For peer review only - http://bmjopen.bmj.com/site/about/guidelines.xhtml

References

1. Zhang W, Robertson J, Jones AC, et al. The placebo effect and its determinants in osteoarthritis: meta-analysis of randomised controlled trials. Ann Rheum Dis 2008;**67**(12):1716-23.
2. Hrobjartsson A, Gotzsche PC. Placebo interventions for all clinical conditions. Cochrane Database of Systematic Reviews 2010;**1**:Art. No.: CD003974-DOI: 10.1002/14651858.CD003974.pub3.
3. Vase L, Riley JL, Price DD. A comparison of placebo effects in clinical analgesic trials versus studies of placebo analgesia. Pain 2002;**99**(3):443-52.
4. Peerdeman KJ, van Laarhoven AIM, Keij SM, et al. Relieving patients' pain with expectation interventions: a meta-analysis. Pain 2016;**157**(6):1179-91.
5. Brody H. The placebo response. J Fam Pract 2000;**49**:649-54.
6. Moerman DE, Jonas WB. Deconstructing the placebo effect and finding the meaning response. Ann Intern Med 2002;**136**(6):471-76.
7. Colloca L, Miller FG. How placebo responses are formed: a learning perspective. Philosophical Transactions of the Royal Society B: Biological Sciences 2011;**366**(1572):1859-69.
8. Howick J, Friedemann C, Tsakok M, et al. Are Treatments More Effective than Placebos? A Systematic Review and Meta-Analysis. PLoS One 2013;**8**(5):e62599.
9. Doherty M, Dieppe P. The "placebo" response in osteoarthritis and its implications for clinical practice. Osteoarthritis Cartilage 2009;**17**(10):1255-62.
10. Lichtenberg P, Heresco-Levy U, Nitzan U. The ethics of the placebo in clinical practice. J Med Ethics 2004;**30**:551-54.
11. Colloca L, Miller FG. Harnessing the placebo effect: the need for translational research. Philosophical Transactions of the Royal Society B: Biological Sciences 2011;**366**(1572):1922-30.
12. Benedetti F, Pollo A, Lopiano L, et al. Conscious expectation and unconscious conditioning in analgesic, motor, and hormonal placebo/nocebo responses. The Journal of Neuroscience 2003;**23**(10):4315-23.
13. Bingel U, Wanigasekera V, Wiech K, et al. The Effect of Treatment Expectation on Drug Efficacy: Imaging the Analgesic Benefit of the Opioid Remifentanil. Science Translational Medicine 2011;**3**(70):70ra14-70ra14.
14. Kaptchuk TJ, Friedlander E, Kelley JM, et al. Placebos without deception: A randomized controlled trial in Irritable Bowel Syndrome. PLoS One 2010;**5**(12):e15591.
15. Kelley JM, Kaptchuk TJ, Cusin C, et al. Open-label placebo for major depressive disorder: a pilot randomized-controlled trial. Psychother Psychosom 2012;**81**:312-14.
16. Blease C, Colloca L, Kaptchuk TJ. Are open-Label Placebos Ethical? Informed Consent and Ethical Equivocations. Bioethics 2016;**30**(6):407-14.
17. Barnhill A, Miller FG. The ethics of placebo treatments in clinical practice: a reply to Glackin. J Med Ethics 2015.
18. Enck P, Bingel U, Schedlowski M, et al. The placebo response in medicine: minimize, maximize or personalize? Nat Rev Drug Discov 2013;**12**(3):191-204.
19. Linde K, F„ssler M, Meissner K. Placebo interventions, placebo effects and clinical practice. Philosophical Transactions of the Royal Society B: Biological Sciences 2011;**366**(1572):1905-12.
20. Klinger R, Colloca L, Bingel U, et al. Placebo analgesia: Clinical applications. Pain 2014;**155**(6):1055-58.
21. Kaptchuk TJ, Miller FG. Placebo Effects in Medicine. N Engl J Med 2015;**373**(1):8-9.
22. Dowell D, Haegerich TM, Chou R. CDc guideline for prescribing opioids for chronic pain—united states, 2016. JAMA 2016;**315**(15):1624-45.
23. Colloca L, Klinger R, Flor H, et al. Placebo analgesia: psychological and neurobiological mechanisms. Pain 2013;**154**(4):511-4.
24. Price DD, Finniss DG, Benedetti F. A comprehensive review of the placebo effect: Recent advances and current thought. Annu Rev Psychol 2008;**59**:565-90.

For peer review only

For peer review only - http://bmjopen.bmj.com/site/about/guidelines.xhtml

25. Finniss DG, Kaptchuk TJ, Miller FG, et al. Biological, clinical, and ethical advances of placebo effects. Lancet 2010;**375**(9715):686-95.
26. Manchikanti L, Giordano J, Fellows B, et al. Placebo and nocebo in interventional pain management: A friend or a foe - or simply foes? Pain Physician 2011;**14**:E157-E75.
27. Puhl AA, Reinhart CJ, Rok ER, et al. An examination of the observed placebo effect associated with the treatment of low back pain - a systematic review. Pain Research & Management 2011;**16**(1):45-52.
28. Faria V, Fredrikson M, Furmark T. Imaging the placebo response: A neurofunctional review. Eur Neuropsychopharmacol 2008;**18**(7):473-85.
29. Vase L, Petersen GL, Riley Iii JL, et al. Factors contributing to large analgesic effects in placebo mechanism studies conducted between 2002 and 2007. Pain 2009;**145**(1-2):36-44.
30. Kirsch I. Placebo psychotherapy: Synonym or oxymoron? J Clin Psychol 2005;**61**(7):791-803.
31. Di Blasi Z, Harkness E, Ernst E, et al. Influence of context effects on health outcomes: a systematic review. Lancet 2001;**357**:757-62.
32. Strauss A, Corbin J. *Basics of Qualitative Research Techniques and Procedures for Developing Grounded Theory*. Thousand Oaks: Sage, 1998.
33. Meissner K, Kohls N, Colloca L. Introduction to placebo effects in medicine: mechanisms and clinical implications. Philosophical Transactions of the Royal Society B: Biological Sciences 2011;**366**(1572):1783-89.
34. Price DD, Chung SK, Robinson ME. Conditioning, expectation, and desire for relief in placebo analgesia. Seminars in Pain Medicine 2005;**3**(1):15-21.
35. Benedetti F, Amanzio M. The placebo response: How words and rituals change the patient's brain. Patient Educ Couns 2011;**84**(3):413-19.
36. Goffaux P, L,onard G, Marchand S, et al. Placebo analgesia. In: Beaulieu P, Lussier D, Porreca F, et al., eds. Pharmacology of Pain. Seattle, WA: IASP Press, 2010:451-73.
37. Kirsch I. Response expectancy theory and application: A decennial review. Appl Prev Psychol 1997;**6**(2):69-79.
38. Hyland ME, Whalley B. Motivational concordance: An important mechanism in self-help therapeutic rituals involving inert (placebo) substances. J Psychosom Res 2008;**65**(5):405-13.
39. Stewart-Williams S, Podd J. The placebo-effect: Dissolving the expectancy versus conditioning debate. Psychol Bull 2004;**130** (2):324-40.
40. Benedetti F. Placebo and the New Physiology of the Doctor-Patient Relationship. Physiol Rev 2013;**93**(3):1207-46.
41. Jubb J, Bensing JM. The sweetest pill to swallow: How patient neurobiology can be harnessed to maximise placebo effects. Neurosci Biobehav Rev 2013;**37**(10, Part 2):2709-20.
42. Benedetti F, Maggi G, Lopiano L, et al. Open versus hidden medical treatments: The patient's knowledge about a therapy affects the therapy outcome. Prevention & Treatment 2003;**6**(1):No.
43. Sandler AD, Bodfish JW. Open-label use of placebos in the treatment of ADHD: a pilot study. Child Care Health Dev 2008;**34**(1):104-10.
44. Mistiaen P, van Osch M, van Vliet L, et al. The effect of patient-practitioner communication on pain: a systematic review. Eur J Pain 2015.
45. Planès S, Villier C, Mallaret M. The nocebo effect of drugs. Pharmacology Research & Perspectives 2016;**4**(2):n/a-n/a.
46. Colloca L, Finniss D. Nocebo effects, patient-clinician communication, and therapeutic outcomes. JAMA 2012;**307**(6):567-8.
47. Witt CM, Martins F, Willich SN, et al. Can I help you? Physicians' expectations as predictor for treatment outcome. European Journal of Pain 2012;**16**(10):1455-66.
48. White P, Bishop FL, Prescott P, et al. Practice, practitioner or placebo? A multifactorial, mixed methods randomized controlled trial of acupuncture. Pain 2012;**153**:455-62.

For peer review only

49. Rice T. 'The hallmark of a doctor': the stethoscope and the making of medical identity. Journal of Material Culture 2010;**15**(3):287-301.
50. Snyder CF, Aaronson NK, Choucair AK, et al. Implementing patient-reported outcomes assessment in clinical practice: a review of the options and considerations. Qual Life Res 2012;**21**(8):1305-14.
51. Benedetti F, Carlino E, Piedimonte A. Increasing uncertainty in CNS clinical trials: the role of placebo, nocebo, and Hawthorne effects. The Lancet Neurology;**15**(7):736-47.
52. Drahota A, Ward D, Mackenzie H, et al. Sensory environment on health-related outcomes of hospital patients (Review). Cochrane Database of Systematic Reviews 2012;**3**:Art. No.: CD005315. DOI: 10.1002/14651858.CD005315.pub2.
53. Dijkstra K, Pieterse M, Pruyn A. Physical environmental stimuli that turn healthcare facilities into healing environments through psychologically mediated effects: systematic review. J Adv Nurs 2006;**56**(2):166-81.
54. Feffer K, Lichtenberg P, Becker G, et al. A comparative study with depressed patients on the acceptability of placebo use. Gen Hosp Psychiatry 2016;**41**:53-6.
55. Ortiz R, Chandros Hull S, Colloca L. Patient attitudes about the clinical use of placebo: qualitative perspectives from a telephone survey. BMJ Open 2016;**6**(4).
56. Bishop FL, Howick J, Heneghan C, et al. Placebo use in the United Kingdom: a qualitative study exploring GPs' views on placebo effects in clinical practice. Fam Pract 2014;**Advance Access**:1-7. doi:10.1093/fampra/cmu016.
57. Bishop FL, Aizlewood L, Adams AEM. When and Why Placebo-Prescribing Is Acceptable and Unacceptable: A Focus Group Study of Patients' Views. PLoS One 2014;**9**(7):e101822.
58. Street, Jr., Makoul G, Arora NK, et al. How does communication heal? Pathways linking clinician-patient communication to health outcomes. Patient Educ Couns 2009;**74**(3):295-301.
59. Greville-Harris M, Dieppe P. Bad Is More Powerful than Good: The Nocebo Response in Medical Consultations. The American Journal of Medicine 2015;**128**(2):126-29.
60. Bensing JM, Verheul W. The silent healer: The role of communication in placebo effects. Patient Educ Couns 2010;**80**(3):293-99.
61. Kirsch I. Conditioning, expectancy, and the placebo effect: Comment on Stewart-Williams and Podd (2004). Psychol Bull 2004;**130**(2):341-43.
62. Fassler M, Gnadinger M, Rosemann T, et al. Use of placebo interventions among Swiss primary care providers. BMC Health Serv Res 2009;**9**(1):144.
63. Howick J, Bishop FL, Heneghan C, et al. Placebo use in the United Kingdom: results from a national survey of primary care practitioners. PLoS One 2013;**8**(3):e58247. doi:10.1371/journal.pone.0058247.
64. Kirsch I, Sapirstein G. Listening to prozac but hearing placebo: A meta-analysis of antidepressant medication. Prevention & Treatment 1998;**1** Article 0002a, posted June 26, 1998.
65. Patel SM, Stason WB, Legedza A, et al. The placebo effect in irritable bowel syndrome trials: a meta-analysis. Neurogastroenterol Motil 2005;**17**(3):332-40.
66. Ford AC, Moayyedi P. Meta-analysis: factors affecting placebo response rate in the irritable bowel syndrome. Aliment Pharmacol Ther 2010;**32**(2):144-58.
67. Bélanger L, Vallières A, Ivers H, et al. Meta-analysis of sleep changes in control groups of insomnia treatment trials. J Sleep Res 2007;**16**(1):77-84.
68. Kaptchuk TJ, Kelley JM, Conboy LA, et al. Components of placebo effect: randomised controlled trial in patients with irritable bowel syndrome. Br Med J 2008;**336**:999-1003.
69. Suarez-Almazor ME, Looney C, Liu Y, et al. A randomized controlled trial of acupuncture for osteoarthritis of the knee: Effects of patient-provider communication. Arthritis Care Res 2010;**62**(9):1229-36.
70. Kaptchuk TJ, Stason WB, Davis RB, et al. Sham device v inert pill: randomised controlled trial of two placebo treatments. Br Med J 2006;**332**(7538):391-94.

For peer review only

71. Vase L, Baram S, Takakura N, et al. Specifying the nonspecific components of acupuncture analgesia. Pain 2013;**154**(9):1659-67.
72. Sandler A, Glesne C, Geller G. Children's and parents' perspectives on open-label use of placebos in the treatment of ADHD. Child Care Health Dev 2008;**34**(1):111-20.
73. Michie S, Abraham C, Whittington C, et al. Effective techniques in healthy eating and physical activity interventions: a meta-regression. Health Psychol 2009;**28**(6):690-701.

For peer review only

For peer review only - http://bmjopen.bmj.com/site/about/guidelines.xhtml

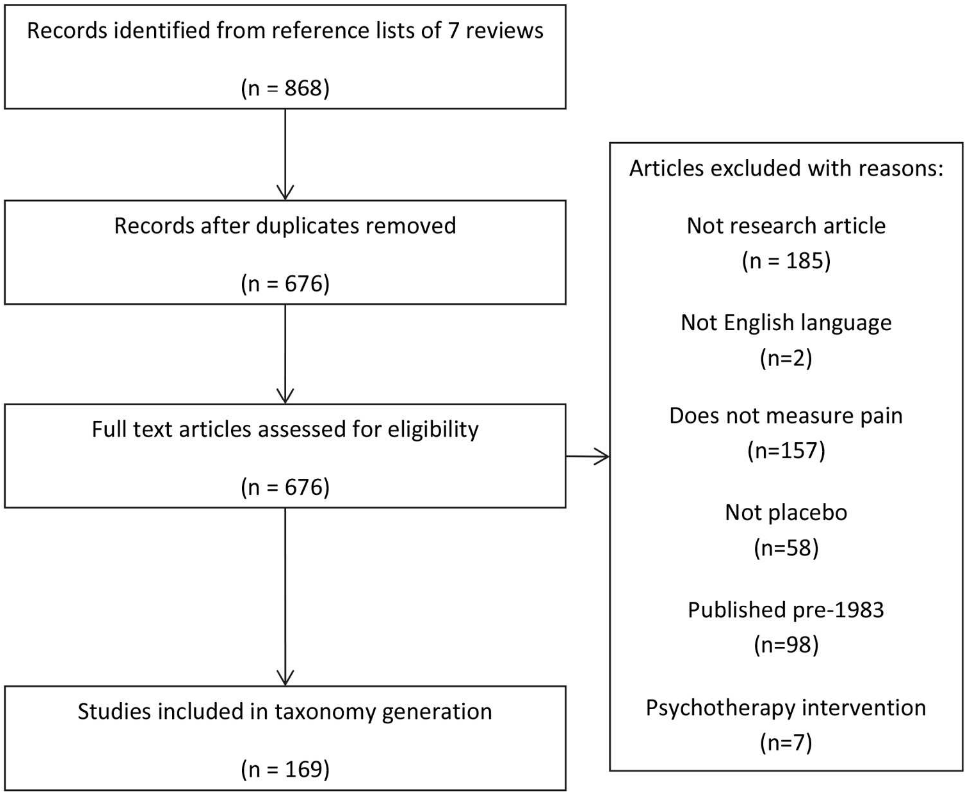

Flowchart Showing Identification of Studies

155x127mm (300 x 300 DPI)

w only

Supplementary Digital Content

List of Included Studies

[1-169]

[1] Alfano AP, Taylor AG, Foresman PA, Dunk PR, McConnell GG, Gillies GT. Static magnetic fields for treatment of fibromyalgia: A randomized controlled trial. . J Altern Complement Med 2001;7(1):53.
[2] Alford JW, Fadale PD. Evaluation of postoperative bupivacaine infusion for pain management after anterior cruciate ligament reconstruction. Arthroscopy-the Journal of Arthroscopic and Related Surgery 2003;19(8):855.
[3] Amanzio M, Benedetti F. Neuropharmacological Dissection of Placebo Analgesia: Expectation-Activated Opioid Systems versus Conditioning-Activated Specific Subsystems. The Journal of Neuroscience 1999;19(1):484-494.
[4] Amanzio M, Pollo A, Maggi G, Benedetti F. Response variability to analgesics: a role for non-specific activation of endogenous opioids. Pain 2001;90(3):205-215.
[5] Amlie E, Weber H, Holme I. Treatment of Acute Low-back Pain with Piroxicam: Results of a Double-blind Placebo-controlled Trial. Spine 1987;12(5):473-476.
[6] Andersen AN, Damm P, Tabor A, Pedersen IM, Harring M. Prevention of Breast Pain and Milk Secretion with Bromocriptine After Second-Trimester Abortion. Acta Obstet Gynecol Scand 1990;69(3):235-238.
[7] Aslaksen PM, Flaten MA. The Roles of Physiological and Subjective Stress in the Effectiveness of a Placebo on Experimentally Induced Pain. Psychosom Med 2008;70(7):811-818.
[8] Atkinson JH, Slater MA, Williams RA, Zisook S, Patterson TL, Grant I, Wahlgren DR, Abramson I, Garfin SR. A placebo-controlled randomized clinical trial of nortriptyline for chronic low back pain. Pain 1998;76(3):287-296.
[9] Bannwarth B, Allaert FA, Avouac B, Rossignol M, Rozenberg S, Valat JP. A randomized, double-blind, placebo controlled triphosphate in study of oral adenosine subacute low back pain. J Rheumatol 2005;32(6):1114-1117.
[10] Basford JR, Sheffield CG, Harmsen WS. Laser therapy: a randomized, controlled trial of the effects of low-intensity Nd:YAG laser irradiation on musculoskeletal back pain. Arch Phys Med Rehabil 1999;80(6):647-652.
[11] Basmajian JV. Acute back pain and spasm. A controlled multicenter trial of combined analgesic and antispasm agents. Spine (Phila Pa 1976) 1989;14(4):438-439.
[12] Benedetti F. The opposite effects of the opiate antagonist naloxone and the cholecystokinin antagonist proglumide on placebo analgesia. Pain 1996;64(3):535-543.
[13] Benedetti F, Amanzio M, Baldi S, Casadio C, Cavallo A, Mancuso M, Ruffini E, Oliaro A, Maggi G. The specific effects of prior opioid exposure on placebo analgesia and placebo respiratory depression. Pain 1998;75(2-3):313-319.
[14] Benedetti F, Amanzio M, Casadio C, Oliaro A, Maggi G. Blockade of nocebo hyperalgesia by the cholecystokinin antagonist proglumide. Pain 1997;71(2):135-140.
[15] Benedetti F, Amanzio M, Maggi G. Potentiation of placebo analgesia by proglumide. The Lancet 1995;346(8984):1231.
[16] Benedetti F, Amanzio M, Vighetti S, Asteggiano G. The Biochemical and Neuroendocrine Bases of the Hyperalgesic Nocebo Effect. The Journal of Neuroscience 2006;26(46):12014-12022.
[17] Benedetti F, Arduino C, Amanzio M. Somatotopic activation of opioid systems by target-directed expectations of analgesia. J Neurosci 1999;19(9):3639-3648.
[18] Benedetti F, Arduino C, Costa S, Vighetti S, Tarenzi L, Rainero I, Asteggiano G. Loss of expectation-related mechanisms in Alzheimer's disease makes analgesic therapies less effective. Pain 2006;121(1):133-144.

[19] Benedetti F, Pollo A, Lopiano L, Lanotte M, Vighetti S, Rainero I. Conscious expectation and unconscious conditioning in analgesic, motor, and hormonal placebo/nocebo responses. The Journal of Neuroscience 2003;23(10):4315-4323.
[20] Benedetti FMD, Amanzio MMS, Casadio CMD, Cavallo AMD, Cianci RMD, Giobbe RMD, Mancuso MMD, Ruffini EMD, Maggi GMD. Control of Postoperative Pain by Transcutaneous Electrical Nerve Stimulation After Thoracic Operations. The Annals of Thoracic Surgery 1997;63(3):773-776.
[21] Berry H, Hutchinson DR. A multicentre placebo-controlled study in general practice to evaluate the efficacy and safety of tizanidine in acute low-back pain. J Int Med Res 1988;16(2):75-82.
[22] Bingel U, Lorenz J, Schoell E, Weiller C, Büchel C. Mechanisms of placebo analgesia: rACC recruitment of a subcortical antinociceptive network. Pain 2006;120(1):8-15.
[23] Birbara CA, Puopolo AD, Munoz DR, Sheldon EA, Mangione A, Bohidar NR, Geba GP. Treatment of chronic low back pain with etoricoxib, a new cyclo-oxygenase-2 selective inhibitor: improvement in pain and disability--a randomized, placebo-controlled, 3-month trial. The journal of pain : official journal of the American Pain Society 2003;4(6):307-315.
[24] Biro P, Meier T, Cummins AS. Comparison of topical anaesthesia methods for venous cannulation in adults. Eur J Pain 1997;1(1):37-42.
[25] Blanchard EB, Appelbaum KA, Radnitz CL, Michultka D, Morrill B, Kirsch C, Hillhouse J, Evans DD, Guarnieri P, Attanasio V, et al. Placebo-controlled evaluation of abbreviated progressive muscle relaxation and of relaxation combined with cognitive therapy in the treatment of tension headache. J Consult Clin Psychol 1990;58(2):210-215.
[26] Blanchard EB, Appelbaum KA, Radnitz CL, Morrill B, Michultka D, Kirsch C, Guarnieri P, Hillhouse J, Evans DD, Jaccard J, et al. A controlled evaluation of thermal biofeedback and thermal biofeedback combined with cognitive therapy in the treatment of vascular headache. J Consult Clin Psychol 1990;58(2):216-224.
[27] Boissel JP, Philippon AM, Gauthier E, Schbath J, Destors JM. Time course of long-term placebo therapy effects in angina pectoris. Eur Heart J 1986;7(12):1030-1036.
[28] Bova JG, Bhattacharjee N, Jurdi R, Bennett WF. Comparison of no medication, placebo, and hyoscyamine for reducing pain during a barium enema. Am J Roentgenol 1999;172(5):1285-1287.
[29] Brinkhaus B, Witt CM, Jena S, Linde K, Streng A, Wagenpfeil S, Irnich D, Walther HU, Melchart D, Willich SN. Acupuncture in patients with chronic low back pain - A randomized controlled trial. Arch Intern Med 2006;166(4):450-457.
[30] Camilleri M, Northcutt AR, Kong S, Dukes GE, McSorley D, Mangel AW. Efficacy and safety of alosetron in women with irritable bowel syndrome: a randomised, placebo-controlled trial. The Lancet 2000;355(9209):1035-1040.
[31] Carbajal R, Chauvet X, Couderc S, Olivier-Martin M. Randomised trial of analgesic effects of sucrose, glucose, and pacifiers in term neonates. BMJ 1999;319(7222):1393-1397.
[32] Carette S, Leclaire R, Marcoux S, Morin F, Blaise GA, St-Pierre A, Truchon R, Parent F, Levesque J, Bergeron V, Montminy P, Blanchette C. Epidural corticosteroid injections for sciatica due to herniated nucleus pulposus. N Engl J Med 1997;336(23):1634-1640.
[33] Charron J, Rainville P, Marchand S. Direct comparison of placebo effects on clinical and experimental pain. Clin J Pain 2006;22(2):204-211.
[34] Cherkin DC, Sherman KJ, Avins AL, et al. A randomized trial comparing acupuncture, simulated acupuncture, and usual care for chronic low back pain. Arch Intern Med 2009;169(9):858-866.
[35] Chrubasik S, Eisenberg E, Balan E, Weinberger T, Luzzati R, Conradt C. Treatment of low back pain exacerbations with willow bark extract: a randomized double-blind study. Am J Med 2000;109(1):9-14.

For peer review only

[36] Chrubasik S, Junck H, Breitschwerdt H, Conradt C, Zappe H. Effectiveness of Harpagophytum extract WS 1531 in the treatment of exacerbation of low back pain: a randomized, placebo-controlled, double-blind study. Eur J Anaesthesiol 1999;16(2):118-129.

[37] Chung SK, Price DD, Verne GN, Robinson ME. Revelation of a personal placebo response: Its effects on mood, attitudes and future placebo responding. Pain 2007;132(3):281-288.

[38] Classen W, Feingold E, Netter P. Influence of sensory suggestibility on treatment outcome in headache patients. Neuropsychobiology 1983;10(1):44-47.

[39] Coats TL, Borenstein DG, Nangia NK, Brown MT. Effects of valdecoxib in the treatment of chronic low back pain: results of a randomized, placebo-controlled trial. Clin Ther 2004;26(8):1249-1260.

[40] Colloca L, Benedetti F. How prior experience shapes placebo analgesia. Pain 2006;124(1-2):126-133.

[41] Colloca L, Benedetti F. Placebo analgesia induced by social observational learning. Pain 2009;144(1-2):28-34.

[42] Colloca L, Petrovic P, Wager TD, Ingvar M, Benedetti F. How the number of learning trials affects placebo and nocebo responses. Pain 2010;151(2):430-439.

[43] Colloca L, Sigaudo M, Benedetti F. The role of learning in nocebo and placebo effects. Pain 2008;136(1-2):211-218.

[44] Conn IG, Marshall AH, Yadav SN, Daly JC, Jaffer M. Transcutaneous electrical nerve stimulation following appendicectomy: the placebo effect. Ann R Coll Surg Engl 1986;68(4):191-192.

[45] Corson SL, Batzer FR, Gocial B, Kelly M, Gutmann JN, Go KJ, English ME. Is paracervical block anesthesia for oocyte retrieval effective? Fertil Steril 1994;62(1):133-136.

[46] Costello M, Ramundo M, Christopher NC, Powell KR. Ethyl Vinyl Chloride Vapocoolant Spray Fails to Decrease Pain Associated with Intravenous Cannulation in Children. Clin Pediatr (Phila) 2006;45(7):628-632.

[47] Coyne PJ, MacMurren M, Izzo T, Kramer T. Transcutaneous electrical nerve stimulator for procedural pain associated with intravenous needlesticks. J Intraven Nurs 1995;18(5):263-267.

[48] Dapas F, Hartman SF, Martinez L, Northrup BE, Nussdorf RT, Silberman HM, Gross H. Baclofen for the treatment of acute low-back syndrome. A double-blind comparison with placebo. Spine (Phila Pa 1976) 1985;10(4):345-349.

[49] De Pascalis V, Chiaradia C, Carotenuto E. The contribution of suggestibility and expectation to placebo analgesia phenomenon in an experimental setting. Pain 2002;96(3):393-402.

[50] deCharms RC, Maeda F, Glover GH, Ludlow D, Pauly JM, Soneji D, Gabrieli JDE, Mackey SC. Control over brain activation and pain learned by using real-time functional MRI. Proc Natl Acad Sci U S A 2005;102(51):18626-18631.

[51] Defrin R, Ariel E, Peretz C. Segmental noxious versus innocuous electrical stimulation for chronic pain relief and the effect of fading sensation during treatment. Pain 2005;115(1):152-160.

[52] Dickens C, Jayson M, Sutton C, Creed F. The relationship between pain and depression in a trial using paroxetine in sufferers of chronic low back pain. Psychosomatics 2000;41(6):490-499.

[53] Ditto B, France CR. The effects of applied tension on symptoms in French-speaking blood donors: a randomized trial. Health Psychol 2006;25(3):433-437.

[54] Ditto B, France CR, Lavoie P, Roussos M, Adler PS. Reducing reactions to blood donation with applied muscle tension: a randomized controlled trial. Transfusion (Paris) 2003;43(9):1269-1275.

[55] Dreiser RL, Marty M, Ionescu E, Gold M, Liu JH. Relief of acute low back pain with diclofenac-K 12.5 mg tablets: a flexible dose, ibuprofen 200 mg and placebo-controlled clinical trial. Int J Clin Pharmacol Ther 2003;41(9):375-385.

[56] Erdogmus CB, Resch KL, Sabitzer R, Muller H, Nuhr M, Schoggl A, Posch M, Osterode W, Ungersbock K, Ebenbichler GR. Physiotherapy-based rehabilitation following disc herniation

For peer review only

operation: results of a randomized clinical trial. Spine (Phila Pa 1976) 2007;32(19):2041-2049.
[57] Faas A, Chavannes AW, van Eijk JT, Gubbels JW. A randomized, placebo-controlled trial of exercise therapy in patients with acute low back pain. Spine (Phila Pa 1976) 1993;18(11):1388-1395.
[58] Fanti L, Gemma M, Passaretti S, Guslandi M, Testoni PA, Casati A, Torri G. Electroacupuncture Analgesia for Colonoscopy: A Prospective, Randomized, Placebo-Controlled Study. Am J Gastroenterol 2003;98(2):312-316.
[59] Forster EL, Kramer JF, Lucy SD, Scudds RA, Novick RJ. EFfect of tens on pain, medications, and pulmonary function following coronary artery bypass graft surgery. CHEST Journal 1994;106(5):1343-1348.
[60] Foster KA, Liskin J, Cen S, Abbott A, Armisen V, Globe D, Knox L, Mitchell M, Shtir C, Azen S. The Trager approach in the treatment of chronic headache: a pilot study. Altern Ther Health Med 2004;10(5):40-46.
[61] Foster NE, Thomas E, Barlas P, Hill JC, Young J, Mason E, Hay EM. Acupuncture as an adjunct to exercise based physiotherapy for osteoarthritis of the knee: randomised controlled trial. BMJ 2007;335(7617):436.
[62] Frega A, Stentella P, Di Renzi F, Gallo G, Palazzetti PL, Del Vescovo M, Ciccarone M, Pachi A. Pain evaluation during carbon dioxide laser vaporization for cervical intraepithelial neoplasia: a randomized trial. Clin Exp Obstet Gynecol 1994;21(3):188-191.
[63] Gale GD, Rothbart PJ, Li Y. Infrared therapy for chronic low back pain: a randomized, controlled trial. Pain Res Manag 2006;11(3):193-196.
[64] Geers A, Helfer S, Weiland P, Kosbab K. Expectations and Placebo Response: A Laboratory Investigation into the Role of Somatic Focus. J Behav Med 2006;29(2):171-178.
[65] Geers AL, Wellman JA, Fowler SL, Helfer SG, France CR. Dispositional optimism predicts placebo analgesia. J Pain 2010;11(11):1165-1171.
[66] Goffaux P, Redmond WJ, Rainville P, Marchand S. Descending analgesia--when the spine echoes what the brain expects. Pain 2007;130(1-2):137-143.
[67] Goodenough B, Kampel L, Champion GD, Laubreaux L, Nicholas MK, Ziegler JB, McInerney M. An investigation of the placebo effect and age-related factors in the report of needle pain from venipuncture in children. Pain 1997;72(3):383-391.
[68] Goodkin K, Gullion CM, Agras WS. A randomized, double-blind, placebo-controlled trial of trazodone hydrochloride in chronic low back pain syndrome. J Clin Psychopharmacol 1990;10(4):269-278.
[69] Grevert P, Albert LH, Goldstein A. Partial antagonism of placebo analgesia by naloxone. Pain 1983;16(2):129-143.
[70] Haake M, Muller HH, Schade-Brittinger C, Basler HD, Schafer H, Maier C, Endres HG, Trampisch HJ, Molsberger A. German acupuncture trials (GERAC) for chronic low back pain. Randomized, multicenter, blinded, parallel-group trial with 3 groups Arch Intern Med 2007;167(17):1892-1898.
[71] Hale ME, Ahdieh H, Ma T, Rauck R. Efficacy and safety of OPANA ER (oxymorphone extended release) for relief of moderate to severe chronic low back pain in opioid-experienced patients: a 12-week, randomized, double-blind, placebo-controlled study. The journal of pain : official journal of the American Pain Society 2007;8(2):175-184.
[72] Hargreaves A, Lander J. Use of Transcutaneous Electrical Nerve Stimulation For Postoperative Pain. Nurs Res 1989;38(3):159-160.
[73] Hargreaves KM, Dionne RA, Mueller GP. Plasma Beta-Endorphin-like Immunoreactivity, Pain and Anxiety Following Administration of Placebo in Oral Surgery Patients. J Dent Res 1983;62(11):1170-1173.
[74] Hashish I, Hai HK, Harvey W, Feinmann C, Harris M. Reduction of postoperative pain and swelling by ultrasound treatment: a placebo effect. Pain 1988;33(3):303-311.

For peer review only

[75] Hashish I, Harvey W, Harris M. Anti-inflammatory effects of ultrasound therapy: evidence for a major placebo effect. Rheumatology (Oxford) 1986;25(1):77-81.

[76] Helms JM. Acupuncture for the management of primary dysmenorrhea. Obstet Gynecol 1987;69(1):51-56.

[77] Hoirus KT, Pfleger B, McDuffie FC, Cotsonis G, Elsangak O, Hinson R, Verzosa GT. A randomized clinical trial comparing chiropractic adjustments to muscle relaxants for subacute low back pain. J Manipulative Physiol Ther 2004;27(6):388-398.

[78] Hong C-Z, Chen Y-C, Pon CH, Yu J. Immediate Effects of Various Physical Medicine Modalities on Pain Threshold of an Active Myofascial Trigger Point. Journal of Musculoskeletal Pain 1993;1(2):37-53.

[79] Hruby G, Ames C, Chen C, Yan Y, Sagar J, Baron P, Landman J. Assessment of efficacy of transcutaneous electrical nerve stimulation for pain management during office-based flexible cystoscopy. Urology 2006;67(5):914-917.

[80] Hyland MR, Webber-Gaffney A, Cohen L, Lichtman SW. Randomized Controlled Trial of Calcaneal Taping, Sham Taping, and Plantar Fascia Stretching for the Short-Term Management of Plantar Heel Pain. J Orthop Sports Phys Ther 2006;36(6):364-371.

[81] Johansen O, Brox J, Flaten MA. Placebo and Nocebo Responses, Cortisol, and Circulating Beta-Endorphin. Psychosom Med 2003;65(5):786-790.

[82] Kaptchuk TJ, Stason WB, Davis RB, Legedza ATR, Schnyer RN, Kerr CE, Stone DA, Nam BH, Kirsch I, Goldman RH. Sham device v inert pill: randomised controlled trial of two placebo treatments. Br Med J 2006;332(7538):391-394.

[83] Katz J, Pennella-Vaughan J, Hetzel RD, Kanazi GE, Dworkin RH. A Randomized, Placebo-Controlled Trial of Bupropion Sustained Release in Chronic Low Back Pain. J Pain 2005;6(10):656-661.

[84] Katz N, Ju WD, Krupa DA, Sperling RS, Bozalis Rodgers D, Gertz BJ, Gimbel J, Coleman S, Fisher C, Nabizadeh S, Borenstein D. Efficacy and safety of rofecoxib in patients with chronic low back pain: results from two 4-week, randomized, placebo-controlled, parallel-group, double-blind trials. Spine (Phila Pa 1976) 2003;28(9):851-858; discussion 859.

[85] Katz N, Rauck R, Ahdieh H, Ma T, Gerritsen van der Hoop R, Kerwin R, Podolsky G. A 12-week, randomized, placebo-controlled trial assessing the safety and efficacy of oxymorphone extended release for opioid-naive patients with chronic low back pain. Curr Med Res Opin 2007;23(1):117-128.

[86] Keltner JR, Furst A, Fan C, Redfern R, Inglis B, Fields HL. Isolating the Modulatory Effect of Expectation on Pain Transmission: A Functional Magnetic Resonance Imaging Study. The Journal of Neuroscience 2006;26(16):4437-4443.

[87] Kerr AR, Drexel CA, Spielman AI. The efficacy and safety of 50 mg penicillin G potassium troches for recurrent aphthous ulcers. Oral Surg Oral Med Oral Pathol Oral Radiol Endod 2003;96(6):685-694.

[88] Ketenci A, Ozcan E, Karamursel S. Assessment of efficacy and psychomotor performances of thiocolchicoside and tizanidine in patients with acute low back pain. Int J Clin Pract 2005;59(7):764-770.

[89] Klaber Moffett JA, Richardson PH, Frost H, Osborn A. A placebo controlled double blind trial to evaluate the effectiveness of pulsed short wave therapy for osteoarthritic hip and knee pain. Pain 1996;67(1):121-127.

[90] Klein RG, Eek BC. Low-energy laser treatment and exercise for chronic low back pain: double-blind controlled trial. Arch Phys Med Rehabil 1990;71(1):34-37.

[91] Kober A, Scheck T, Greher M, Lieba F, Fleischhackl R, Fleischhackl S, Randunsky F, Hoerauf K. Prehospital Analgesia with Acupressure in Victims of Minor Trauma: A Prospective, Randomized, Double-Blinded Trial. Anesth Analg 2002;95(3):723-727 710.1213/00000539-200209000-200200035.

For peer review only

[92] Kong J, Gollub RL, Polich G, Kirsch I, LaViolette P, Vangel M, Rosen B, Kaptchuk TJ. A Functional Magnetic Resonance Imaging Study on the Neural Mechanisms of Hyperalgesic Nocebo Effect. The Journal of Neuroscience 2008;28(49):13354-13362.
[93] Kong J, Gollub RL, Rosman IS, Webb JM, Vangel MG, Kirsch I, Kaptchuk TJ. Brain activity associated with expectancy-enhanced placebo analgesia as measured by functional magnetic resonance imaging. J Neurosci 2006;26(2):381-388.
[94] Kotani N, Kushikata T, Suzuki A, Hashimoto H, Muraoka M, Matsuki A. Insertion of intradermal needles into painful points provides analgesia for intractable abdominal scar pain. Reg Anesth Pain Med 2001;26(6):532-538.
[95] Kupers R, Maeyaert J, Boly M, Faymonville ME, Laureys S. Naloxone-insensitive epidural placebo analgesia in a chronic pain patient. Anesthesiology 2007;106(6):1239-1242.
[96] Lander J, Fowler-Kerry S. TENS for children's procedural pain. Pain 1993;52(2):209-216.
[97] Leibing E, Leonhardt U, Koster G, Goerlitz A, Rosenfeldt J, Hilgers R, Ramadori G. Acupuncture treatment of chronic low-back pain - a randomized, blinded, placebo-controlled trial with 9-month follow-up. Pain 2002;96 189-196.
[98] Levine JD, Gordon NC. Influence of the method of drug administration on analgesic response. Nature 1984;312(5996):755-756.
[99] Licciardone JC, Stoll ST, Fulda KG, Russo DP, Siu J, Winn W, Swift JJ. Osteopathic Manipulative Treatment for Chronic Low Back Pain: A Randomized Controlled Trial. Spine 2003;28(13):1355-1362.
[100] Lieberman MD, Jarcho JM, Berman S, Naliboff BD, Suyenobu BY, Mandelkern M, Mayer EA. The neural correlates of placebo effects: a disruption account. Neuroimage 2004;22(1):447-455.
[101] Limoges MF, Rickabaugh B. Evaluation of TENS During Screening Flexible Sigmoidoscopy. Gastroenterol Nurs 2004;27(2):61-68.
[102] Lin J-G, Lo M-W, Wen Y-R, Hsieh C-L, Tsai S-K, Sun W-Z. The effect of high and low frequency electroacupuncture in pain after lower abdominal surgery. Pain 2002;99(3):509-514.
[103] Linde K, Streng A, Jurgens S, Hoppe A, Brinkhaus B, Witt C, Wagenpfeil S, Pfaffenrath V, Hammes MG, Weidenhammer W, Willich SN, Melchart D. Acupuncture for patients with migraine. A randomized controlled trial. Journal of the American Medical Association 2005;293 (2118):2125.
[104] Liossi C, Hatira P. Clinical Hypnosis in the Alleviation of Procedure-Related Pain in Pediatric Oncology Patients. Int J Clin Exp Hypn 2003;51(1):4-28.
[105] Lipman JJ, Miller BE, Mays KS, Miller MN, North WC, Byrne WL. Peak B endorphin concentration in cerebrospinal fluid: reduced in chronic pain patients and increased during the placebo response. Psychopharmacology (Berl) 1990;102(1):112-116.
[106] Manchikanti L, Pampati V, Damron K. The role of placebo and nocebo effects of perioperative administration of sedatives and opioids in interventional pain management. Pain Physician 2005;8(4):349-355.
[107] Martikainen IK, Hagelberg N, Mansikka H, Hietala J, Någren K, Scheinin H, Pertovaara A. Association of striatal dopamine D2/D3 receptor binding potential with pain but not tactile sensitivity or placebo analgesia. Neurosci Lett 2005;376(3):149-153.
[108] Matre D, Casey KL, Knardahl S. Placebo-Induced Changes in Spinal Cord Pain Processing. The Journal of Neuroscience 2006;26(2):559-563.
[109] Mayberg HS, Silva JA, Brannan SK, Tekell JL, Mahurin RK, McGinnis S, Jerabek PA. The functional neuroanatomy of the placebo effect. A J Psychiatry 2002;159(5):728-737.
[110] Melchart D, Streng A, Hoppe A, Brinkhaus B, Witt C, Wagenpfeil S, Pfaffenrath V, Hammes M, Hummelsberger J, Irnich D, Weidenhammer W, Willich SN, Linde K. Acupuncture in patients with tension-type headache: randomised controlled trial. Br Med J 2005;331 376-382.
[111] Molsberger AF, Mau J, Pawelec DB, Winkler J. Does acupuncture improve the orthopedic management of chronic low back pain – a randomized, blinded, controlled trial with 3 months follow up. Pain 2002;99(3):579-587.

For peer review only

[112] Montgomery G, Kirsch I. Mechanisms of Placebo Pain Reduction: An Empirical Investigation. Psychological Science 1996;7(3):174-176.
[113] Montgomery GH, Kirsch I. Classical conditioning and the placebo effect. Pain 1997;72(1-2):107-113.
[114] Morton DL, Watson A, El-Deredy W, Jones AKP. Reproducibility of placebo analgesia: Effect of dispositional optimism. Pain 2009;146(1â€"2):194-198.
[115] Muehlbacher M, Nickel MK, Kettler C, Tritt K, Lahmann C, Leiberich PK, Nickel C, Krawczyk J, Mitterlehner FO, Rother WK, Loew TH, Kaplan P. Topiramate in treatment of patients with chronic low back pain: a randomized, double-blind, placebo-controlled study. Clin J Pain 2006;22(6):526-531.
[116] Nemoto H, Nemoto Y, Toda H, Mikuni M, Fukuyama H. Placebo analgesia: a PET study. Exp Brain Res 2007;179(4):655-664.
[117] Ockene JK, Barad DH, Cochrane BB, Larson JC, Gass M, Wassertheil-Smoller S, Manson JE, Barnabei VM, Lane DS, Brzyski RG, Rosal MC, Wylie-Rosett J, Hays J. Symptom experience after discontinuing use of estrogen plus progestin. JAMA 2005;294(2):183-193.
[118] Pallay RM, Seger W, Adler JL, Ettlinger RE, Quaidoo EA, Lipetz R, O'Brien K, Mucciola L, Skalky CS, Petruschke RA, Bohidar NR, Geba GP. Etoricoxib reduced pain and disability and improved quality of life in patients with chronic low back pain: a 3 month, randomized, controlled trial. Scand J Rheumatol 2004;33(4):257-266.
[119] Pariente J, White P, Frackowiak RSJ, Lewith G. Expectancy and belief modulate the neuronal substrates of pain treated by acupuncture. Neuroimage 2005;25(4):1161-1167.
[120] Peloso PM, Fortin L, Beaulieu A, Kamin M, Rosenthal N. Analgesic efficacy and safety of tramadol/ acetaminophen combination tablets (Ultracet) in treatment of chronic low back pain: a multicenter, outpatient, randomized, double blind, placebo controlled trial. J Rheumatol 2004;31(12):2454-2463.
[121] Petrovic P, Kalso E, Petersson KM, Ingvar M. Placebo and opioid analgesia-- imaging a shared neuronal network. Science 2002;295(5560):1737-1740.
[122] Pollo A, Amanzio M, Arslanian A, Casadio C, Maggi G, Benedetti F. Response expectancies in placebo analgesia and their clinical relevance. Pain 2001;93(1):77-84.
[123] Pollo A, Vighetti S, Rainero I, Benedetti F. Placebo analgesia and the heart. Pain 2003;102(1-2):125-133.
[124] Preyde M. Effectiveness of massage therapy for subacute low-back pain: a randomized controlled trial. Can Med Assoc J 2000;162(13):1815-1820.
[125] Price DD, Craggs J, Nicholas Verne G, Perlstein WM, Robinson ME. Placebo analgesia is accompanied by large reductions in pain-related brain activity in irritable bowel syndrome patients. Pain 2007;127(1):63-72.
[126] Price DD, Long S, Wilsey B, Rafii A. Analysis of peak magnitude and duration of analgesia produced by local anesthetics injected into sympathetic ganglia of complex regional pain syndrome patients. Clin J Pain 1998;14(3):216-226.
[127] Price DD, Milling LS, Kirsch I, Duff A, Montgomery GH, Nicholls SS. An analysis of factors that contribute to the magnitude of placebo analgesia in an experimental paradigm. Pain 1999;83(2):147-156.
[128] Rainville P, Duncan GH, Price DD, Carrier B, Bushnell MC. Pain affect encoded in human anterior cingulate but not somatosensory cortex. Science 1997;277(5328):968-971.
[129] Rawling MJ, Wiebe ER. A randomized controlled trial of fentanyl for abortion pain. Am J Obstet Gynecol 2001;185(1):103-107.
[130] Ristikankare M, Hartikainen J, Heikkinen M, Janatuinen E, Julkunen R. Is routinely given conscious sedation of benefit during colonoscopy? Gastrointest Endosc 1999;49(5):566-572.
[131] Robinson R, Darlow S, Wright SJ, Watters C, Carr I, Gadsby G, Mayberry J. Is transcutaneous electrical nerve stimulation an effective analgesia during colonoscopy? Postgrad Med J 2001;77(909):445-446.

For peer review only

[132] Rowbotham MC, Davies PS, Verkempinck C, Galer BS. Lidocaine patch: double-blind controlled study of a new treatment method for post-herpetic neuralgia. Pain 1996;65(1):39-44.
[133] Ruoff GE, Rosenthal N, Jordan D, Karim R, Kamin M. Tramadol/Acetaminophen combination tablets for the treatment of chronic lower back pain: A multicenter, randomized, double-blind, placebo-controlled outpatient study. Clin Ther 2003;25(4):1123-1141.
[134] Sanders GE, Reinert O, Tepe R, Maloney P. Chiropractic adjustive manipulation on subjects with acute low back pain: visual analog pain scores and plasma beta-endorphin levels. J Manipulative Physiol Ther 1990;13(7):391-395.
[135] Scharf HP, Mansmann U, Streitberger K, Witte S, Kramer J, Maier C, Trampisch HJ, Victor N. Acupuncture and knee osteoarthritis: A three-armed randomized trial. Ann Intern Med 2006;145(1):12-20.
[136] Scharff L, Marcus DA, Masek BJ. A Controlled Study of Minimal-Contact Thermal Biofeedback Treatment in Children With Migraine. J Pediatr Psychol 2002;27(2):109-119.
[137] Schnebel BE, Simmons JW. The use of oral colchicine for low-back pain. A double-blind study. Spine (Phila Pa 1976) 1988;13(3):354-357.
[138] Schnitzer TJ, Gray WL, Paster RZ, Kamin M. Efficacy of tramadol in treatment of chronic low back pain. J Rheumatol 2000;27(3):772-778.
[139] Scott DJ, Stohler CS, Egnatuk CM, Wang H, Koeppe RA, Zubieta J-K. Individual Differences in Reward Responding Explain Placebo-Induced Expectations and Effects. Neuron 2007;55(2):325-336.
[140] Scott DJ, Stohler CS, Egnatuk CM, Wang H, Koeppe RA, Zubieta J. PLacebo and nocebo effects are defined by opposite opioid and dopaminergic responses. Arch Gen Psychiatry 2008;65(2):220-231.
[141] Snyder-Mackler L, Barry AJ, Perkins AI, Soucek MD. Effects of helium-neon laser irradiation on skin resistance and pain in patients with trigger points in the neck or back. Phys Ther 1989;69(5):336-341.
[142] Soriano F, Rios R. Gallium arsenide laser treatment of chronic low back pain: A prospective, randomized and double blind study. Laser Therapy 1998;10(4):175-180.
[143] Stransky M, Rubin A, Lava NS, Lazaro RP. Treatment of carpal tunnel syndrome with vitamin B6: a double-blind study. South Med J 1989;82(7):841-842.
[144] Szpalski M, Hayez JP. Objective functional assessment of the efficacy of tenoxicam in the treatment of acute low back pain. A double-blind placebo-controlled study. Br J Rheumatol 1994;33(1):74-78.
[145] Tashjian RZ, Banerjee R, Bradley MP, Alford W, Fadale PD. Zolpidem reduces postoperative pain, fatigue, and narcotic consumption following knee arthroscopy: a prospective randomized placebo-controlled double-blinded study. J Knee Surg 2006;19(2):105-111.
[146] Theroux MC, West DW, Corddry DH, Hyde PM, Bachrach SJ, Cronan KM, Kettrick RG. Efficacy of intranasal midazolam in facilitating suturing of lacerations in preschool children in the emergency department. Pediatrics 1993;91(3):624-627.
[147] Thomas KS, Muir KR, Doherty M, Jones AC, O'Reilly SC, Bassey EJ. Home based exercise programme for knee pain and knee osteoarthritis: randomised controlled trial. BMJ 2002;325(7367):752.
[148] Toya S, Motegi M, Inomata K, Ohshiro T, Macda T. Report on a computer-randomized double blind clinical trial to determine the effectiveness of the GaAlAs (830 nm) diode laser for pain attenuation in selected pain groups. Laser Therapy 1994;6:143-148.
[149] Tritrakarn T, Lertakyamanee J, Koompong P, Soontrapa S, Somprakit P, Tantiwong A, Jittapapai S. Both EMLA and Placebo Cream Reduced Pain during Extracorporeal Piezoelectric Shock Wave Lithotripsy with the Piezolith 2300. Anesthesiology 2000;92(4):1049-1054.
[150] Vase L, Robinson ME, Verne GN, Price DD. The contributions of suggestion, desire, and expectation to placebo effects in irritable bowel syndrome patients: An empirical investigation. Pain 2003;105(1-2):17-25.

For peer review only

[151] Vase L, Robinson ME, Verne GN, Price DD. Increased placebo analgesia over time in irritable bowel syndrome (IBS) patients is associated with desire and expectation but not endogenous opioid mechanisms. Pain 2005;115(3):338-347.

[152] Verne GN, Robinson ME, Vase L, Price DD. Reversal of visceral and cutaneous hyperalgesia by local rectal anesthesia in irritable bowel syndrome (IBS) patients. Pain 2003;105(1):223-230.

[153] Vondrackova D, Leyendecker P, Meissner W, Hopp M, Szombati I, Hermanns K, Ruckes C, Weber S, Grothe B, Fleischer W, Reimer K. Analgesic efficacy and safety of oxycodone in combination with naloxone as prolonged release tablets in patients with moderate to severe chronic pain. The journal of pain : official journal of the American Pain Society 2008;9(12):1144-1154.

[154] Vorsanger GJ, Xiang J, Gana TJ, Pascual ML, Fleming RR. Extended-release tramadol (tramadol ER) in the treatment of chronic low back pain. J Opioid Manag 2008;4(2):87-97.

[155] Voudouris NJ, Peck CL, Coleman G. Conditioned placebo responses. J Pers Soc Psychol 1985;48(1):47-53.

[156] Voudouris NJ, Peck CL, Coleman G. Conditioned response models of placebo phenomena: further support. Pain 1989;38(1):109-116.

[157] Voudouris NJ, Peck CL, Coleman G. The role of conditioning and verbal expectancy in the placebo response. Pain 1990;43(1):121-128.

[158] Wager TD, Matre D, Casey KL. Placebo effects in laser-evoked pain potentials. Brain Behav Immun 2006;20(3):219-230.

[159] Wager TD, Rilling JK, Smith EE, Sokolik A, Casey KL, Davidson RJ, Kosslyn SM, Rose RM, Cohen JD. Placebo-Induced Changes in fMRI in the Anticipation and Experience of Pain. Science 2004;303(5661):1162-1167.

[160] Wager TD, Scott DJ, Zubieta J-K. Placebo effects on human μ-opioid activity during pain. Proceedings of the National Academy of Sciences 2007;104(26):11056-11061.

[161] Walton RE, Chiappinelli J. Prophylactic penicillin: effect on posttreatment symptoms following root canal treatment of asymptomatic periapical pathosis. J Endod 1993;19(9):466-470.

[162] Wang B, Tang J, White PF, Naruse R, Sloninsky A, Kariger R, Gold J, Wender RH. Effect of the Intensity of Transcutaneous Acupoint Electrical Stimulation on the Postoperative Analgesic Requirement. Anesth Analg 1997;85(2):406-413.

[163] Watson A, El-Deredy W, Bentley DE, Vogt BA, Jones AKP. Categories of placebo response in the absence of site-specific expectation of analgesia. Pain 2006;126(1):115-122.

[164] Watson A, El-Deredy W, Vogt BA, Jones AKP. Placebo analgesia is not due to compliance or habituation: EEG and behavioural evidence. Neuroreport 2007;18(8):771-775 710.1097/WNR.1090b1013e3280c1091e1092a1098.

[165] Webster LR, Butera PG, Moran LV, Wu N, Burns LH, Friedmann N. Oxytrex minimizes physical dependence while providing effective analgesia: a randomized controlled trial in low back pain. The journal of pain : official journal of the American Pain Society 2006;7(12):937-946.

[166] Witt C, Brinkhaus B, Jena S, Linde K, Streng A, Wagenpfeil S, Hummelsberger J, Walther HU, Melchart D, Willich SN. Acupuncture in patients with osteoarthritis of the knee: a randomised trial. Lancet 2005;366 136-143.

[167] Wu M-T, Sheen J-M, Chuang K-H, Yang P, Chin S-L, Tsai C-Y, Chen C-J, Liao J-R, Lai P-H, Chu K-A, Pan H-B, Yang C-F. Neuronal Specificity of Acupuncture Response: A fMRI Study with Electroacupuncture. Neuroimage 2002;16(4):1028-1037.

[168] Zubieta JK, Bueller JA, Jackson LR, Scott DJ, Xu Y, Koeppe RA, Nichols TE, Stohler CS. Placebo Effects Mediated by Endogenous Opioid Activity on æ-Opioid Receptors. The Journal of Neuroscience 2005;25(34):7754-7762.

[169] Zubieta JK, Yau WY, Scott DJ, Stohler CS. Belief or Need? Accounting for individual variations in the neurochemistry of the placebo effect. Brain Behav Immun 2006;20(1):15-26.

For peer review only

PRISMA 2009 Checklist

Section/topic	#	Checklist item	Reported on page #
**TITLE**			
Title	1	Identify the report as a systematic review, meta-analysis, or both.	n/a
**ABSTRACT**			
Structured summary	2	Provide a structured summary including, as applicable: background; objectives; data sources; study eligibility criteria, participants, and interventions; study appraisal and synthesis methods; results; limitations; conclusions and implications of key findings; systematic review registration number.	n/a
**INTRODUCTION**			
Rationale	3	Describe the rationale for the review in the context of what is already known.	3
Objectives	4	Provide an explicit statement of questions being addressed with reference to participants, interventions, comparisons, outcomes, and study design (PICOS).	n/a
**METHODS**			
Protocol and registration	5	Indicate if a review protocol exists, if and where it can be accessed (e.g., Web address), and, if available, provide registration information including registration number.	n/a
Eligibility criteria	6	Specify study characteristics (e.g., PICOS, length of follow-up) and report characteristics (e.g., years considered, language, publication status) used as criteria for eligibility, giving rationale.	5
Information sources	7	Describe all information sources (e.g., databases with dates of coverage, contact with study authors to identify additional studies) in the search and date last searched.	4
Search	8	Present full electronic search strategy for at least one database, including any limits used, such that it could be repeated.	n/a
Study selection	9	State the process for selecting studies (i.e., screening, eligibility, included in systematic review, and, if applicable, included in the meta-analysis).	4, Fig1
Data collection process	10	Describe method of data extraction from reports (e.g., piloted forms, independently, in duplicate) and any processes for obtaining and confirming data from investigators.	6
Data items	11	List and define all variables for which data were sought (e.g., PICOS, funding sources) and any assumptions and simplifications made.	6
Risk of bias in individual studies	12	Describe methods used for assessing risk of bias of individual studies (including specification of whether this was done at the study or outcome level), and how this information is to be used in any data synthesis.	n/a
Summary measures	13	State the principal summary measures (e.g., risk ratio, difference in means).	n/a
Synthesis of results	14	Describe the methods of handling data and combining results of studies, if done, including measures of consistency (e.g., I^2) for each meta-analysis.	6

For peer review only

PRISMA 2009 Checklist

Section/topic	#	Checklist item	Reported on page #
Risk of bias across studies	15	Specify any assessment of risk of bias that may affect the cumulative evidence (e.g., publication bias, selective reporting within studies).	n/a
Additional analyses	16	Describe methods of additional analyses (e.g., sensitivity or subgroup analyses, meta-regression), if done, indicating which were pre-specified.	n/a
**RESULTS**			
Study selection	17	Give numbers of studies screened, assessed for eligibility, and included in the review, with reasons for exclusions at each stage, ideally with a flow diagram.	Fig1
Study characteristics	18	For each study, present characteristics for which data were extracted (e.g., study size, PICOS, follow-up period) and provide the citations.	n/a
Risk of bias within studies	19	Present data on risk of bias of each study and, if available, any outcome level assessment (see item 12).	n/a
Results of individual studies	20	For all outcomes considered (benefits or harms), present, for each study: (a) simple summary data for each intervention group (b) effect estimates and confidence intervals, ideally with a forest plot.	n/a
Synthesis of results	21	Present results of each meta-analysis done, including confidence intervals and measures of consistency.	n/a
Risk of bias across studies	22	Present results of any assessment of risk of bias across studies (see Item 15).	n/a
Additional analysis	23	Give results of additional analyses, if done (e.g., sensitivity or subgroup analyses, meta-regression [see Item 16]).	n/a
**DISCUSSION**			
Summary of evidence	24	Summarize the main findings including the strength of evidence for each main outcome; consider their relevance to key groups (e.g., healthcare providers, users, and policy makers).	21
Limitations	25	Discuss limitations at study and outcome level (e.g., risk of bias), and at review-level (e.g., incomplete retrieval of identified research, reporting bias).	22-3
Conclusions	26	Provide a general interpretation of the results in the context of other evidence, and implications for future research.	22-3
**FUNDING**			
Funding	27	Describe sources of funding for the systematic review and other support (e.g., supply of data); role of funders for the systematic review.	1

From: Moher D, Liberati A, Tetzlaff J, Altman DG, The PRISMA Group (2009). Preferred Reporting Items for Systematic Reviews and Meta-Analyses: The PRISMA Statement. PLoS Med 6(7): e1000097. doi:10.1371/journal.pmed1000097

For more information, visit: www.prisma-statement.org.

For peer review only - http://bmjopen.bmj.com/site/about/guidelines.xhtml

What Techniques Might be Used to Harness Placebo Effects in Non-Malignant Pain? A Literature Review and Survey to Develop a Taxonomy.

Journal:	*BMJ Open*
Manuscript ID	bmjopen-2016-015516.R2
Article Type:	Research
Date Submitted by the Author:	28-Apr-2017

Complete List of Authors:	Bishop, Felicity; University of Southampton, Psychology<br>Coghlan, Beverly; University of Southampton<br>Geraghty, Adam; University of Southampton, Primary Care and Population Sciences<br>Everitt, Hazel; University of Southampton, Primary Care and Population Sciences, Faculty of Medicine<br>Little, Paul; University of Southampton, Primary Care and Population Science;<br>Holmes, Michelle; University of Southampton, Psychology<br>Seretis , Dionysis ; University of Southampton, Psychology<br>Lewith, George; University of Southampton

**Primary Subject Heading**:	Complementary medicine
Secondary Subject Heading:	General practice / Family practice, Research methods, Rheumatology, Rehabilitation medicine
Keywords:	placebos, placebo effect, nocebo effect, translational research, review, classification

SCHOLARONE™ Manuscripts

What Techniques Might be Used to Harness Placebo Effects in Non-Malignant Pain? A Literature Review and Survey to Develop a Taxonomy.

Running head: Taxonomy of techniques to harness placebo effects

Authors: Felicity L Bishop PhD [a,b], Beverly Coghlan MSc [b], Adam WA Geraghty PhD [b], Hazel Everitt PhD [b], Paul Little FMedSci [b], Michelle M Holmes MRes [a], Dionysis Seretis MRes [a], George Lewith MRCGP [b]

a. Department of Psychology, Faculty of Social Human and Mathematical Sciences, Building 44 Highfield Campus, University of Southampton, Southampton SO17 1BJ United Kingdom.

b. Primary Care and Population Sciences, Aldermoor Health Centre, University of Southampton, Southampton, SO16 5ST, United Kingdom.

Emails: Felicity Bishop: F.L.Bishop@southampton.ac.uk; Beverly Coghlan: beverly101@btinternet.com; Adam Geraghty: A.W.Geraghty@soton.ac.uk; Hazel Everitt: H.A.Everitt@soton.ac.uk; Paul Little: P.Little@soton.ac.uk; Michelle Holmes: M.M.Holmes@soton.ac.uk; Dionysis Seretis: ds2g14@soton.ac.uk; George Lewith: gl3@soton.ac.uk.

Corresponding author: Felicity L Bishop. Email: F.L.Bishop@southampton.ac.uk. Phone: +44 (0)23 8059 9020. Fax: +44 (0)23 8059 4597. Psychology, Faculty of Social and Human Sciences, Building 44 Highfield Campus, University of Southampton, Southampton SO17 1BJ United Kingdom.

For peer review only

For peer review only - http://bmjopen.bmj.com/site/about/guidelines.xhtml

Abstract

Objectives. Placebo effects can be clinically meaningful but are seldom fully exploited in clinical practice. This review aimed to facilitate translational research by producing a taxonomy of techniques that could augment placebo analgesia in clinical practice.

Design. Literature review and survey.

Methods. We systematically analysed methods which could plausibly be used to elicit placebo effects in 169 clinical and laboratory-based studies involving non-malignant pain, drawn from 7 systematic reviews. In a validation exercise we surveyed 33 leading placebo researchers (M=12 years' research experience, SD=9.8), who were asked to comment on and add to the draft taxonomy derived from the literature.

Results. The final taxonomy defines 30 procedures that may contribute to placebo effects in clinical and experimental research, proposes 60 possible clinical applications, and classifies procedures into 5 domains: the *Patient's Characteristics and Belief* (5 procedures and 11 clinical applications); the *Practitioner's Characteristics and Beliefs* (2 procedures and 4 clinical applications); the *Healthcare Setting* (8 procedures and 13 clinical applications); *Treatment Characteristics* (8 procedures and 14 clinical applications); and the *Patient-Practitioner Interaction* (7 procedures and 18 clinical applications).

Conclusions. The taxonomy provides a preliminary and novel tool with potential to guide translational research aiming to harness placebo effects for patient benefit in practice.

Keywords: placebos; placebo effect; nocebo effect; translational research; review; classification

For peer review only - http://bmjopen.bmj.com/site/about/guidelines.xhtml

Article Summary

Strengths and Limitations of this Study

- This is a novel attempt to use existing studies to identify the factors that might contribute to placebo effects and the associated procedures that could be simply and ethically adapted for clinical practice, subject to further testing .
- We drew on both clinical trials and laboratory-based studies of placebo effects, in order to generate a more comprehensive list of factors that might contribute to placebo effects than would be possible by relying on just one literature.
- A systematic approach to data synthesis, based on qualitative research methods, was used to identify and classify procedures that might contribute to placebo effects in clinical trials.
- The development of the taxonomy did not incorporate very recent placebo trials or studies and the selection of reviews used to determine which original studies to include in the development process was somewhat arbitrary.
- Our taxonomy is presented not as an exhaustive compilation of current methods used in placebo research but as a detailed and systematic guide for future research, which can in turn further refine the taxonomy.

For peer review only - http://bmjopen.bmj.com/site/about/guidelines.xhtml

Introduction

There is compelling evidence that factors other than the so-called active components of treatment can have clinically meaningful effects on symptoms, particularly non-malignant pain [1-4]. Such 'placebo effects' can be defined as the physiological and/or psychological changes that result from the meaning derived by a person in a health care setting [5 6]. Expectations – which can be generated, for example, by verbal suggestion or previous experience - play a key role in placebo effects.[7] These effects may be as large as treatment effects [8] and occur throughout medicine, especially when doctors and patients interact with each other. They are not routinely deliberately harnessed for patient benefit in clinical practice [9], possibly because doctors often assume they must deceive patients in order to elicit placebo effects [10 11]. However, this assumption is mistaken because it is not necessary to prescribe placebos in order to elicit placebo effects. For example, the overall analgesic effect of an opioid derives not only from its specific pharmaceutical actions but also from its psychological components, i.e. the *expectations* and *meaning* that the patient derives when consulting the doctor and taking the medicine[12 13]. The same is true for other types of intervention including physical, surgical, and psychotherapies. One approach that has received initial support is for doctors to use positive suggestion to enhance patients' expectations of benefit [4]. Furthermore, preliminary evidence suggests that openly prescribing placebos might elicit clinically meaningful placebo effects in IBS and depression [14 15] although this approach entails its own set of ethical challenges[16 17].

Placebo researchers have called for more translational research in this field [11 18-20]. Such work has thus far typically focused on ethical considerations and narrative approaches to drawing out implications for clinical practice from the placebo literature. We suggest a systematic approach to translational research might be helpful. Many techniques or procedures contribute to placebo effects and could potentially be simply and ethically adapted for clinical practice, subject to further

For peer review only - http://bmjopen.bmj.com/site/about/guidelines.xhtml

testing in practice settings [21]. In order to identify and describe such techniques, and thus provide some direction for future research, we reviewed experimental and clinical studies of placebo effects in non-malignant pain. We focused on non-malignant pain because it can be difficult to manage (particularly with current concerns about opioids [22]), the mechanisms underpinning placebo analgesia are reasonably well understood [23], laboratory-based experimental studies often focus on placebo analgesia, and patients with pain have been shown to display substantial and clinically significant placebo effects [1]. The aim of this project was to facilitate translational research by producing a taxonomy of techniques that may contribute to placebo effects observed in research settings and could be studied as options for augmenting placebo enhancement of analgesia in clinical practice.

Methods

Literature Search

We selected seven systematic reviews of different aspects of the placebo literature, chosen from recent reviews available at the time (2012) and based on expert opinion (within the research team) to enable the extraction of information on placebo procedures from a broad range of settings - comprehensive reviews [24-26], reviews of placebo effects in clinical populations [2 27] and reviews of laboratory-based experimental placebo studies [28 29]. The key consideration was that this collection of reviews should cite a diverse set of studies likely to be using diverse methods to directly (e.g. placebo mechanisms studies) or indirectly (e.g. clinical trials with placebo controls) study placebo effects. After removing duplicates and ineligible studies (see Figure 1), 169 studies were used to develop the taxonomy (for a list of included studies see Supplementary Digital Content).

For peer review only - http://bmjopen.bmj.com/site/about/guidelines.xhtml

Studies were eligible for inclusion if they: reported original research in which some participants received a placebo intervention; reported a non-malignant pain outcome; were published since 1983; and were published in English language. Studies were excluded if they: were published before 1983 (because (a) means of generating context-dependent placebo effects may be sensitive to social and cultural changes over time, e.g. patient preferences for particular communication styles and thus their effectiveness in modifying expectations may have changed over time and (b) this yielded a manageable number of papers to analyse which had been published during the 30 years preceding this analysis); or examined any type of psychotherapeutic interventions (because it is difficult to disentangle the active components of psychotherapy from the effect of the meaning of the intervention [30]).

Data Extraction and Synthesis

Descriptions of all events that occurred in the placebo groups during each of the 169 studies (e.g. medical, administrative, and ethical procedures) were extracted into a piloted form by one author and checked by a second. These events were reviewed for duplication and overlap. This resulted in an initial list of 43 procedures that might contribute to placebo effects (e.g. informed consent processes, taking placebo pills, conditioning protocols). Study authors were not contacted for further information about method used.

To synthesise the data and develop our taxonomy we used systematic and rigorous methods derived from qualitative research. We began with a deductive analysis, which aimed to categorise the

For peer review only - http://bmjopen.bmj.com/site/about/guidelines.xhtml

procedures in a way that is intuitively appealing, accessible, and clinically relevant by sorting them into five previously-identified contextual domains of healthcare: patients' characteristics/beliefs, practitioners' characteristics/beliefs, practitioner-patient relationship, superficial treatment characteristics, and the healthcare environment [31]. Two authors (BC, FB) performed the initial categorisation which was then reviewed in detail by three other authors (GL, HE, AG). We then engaged in a constant comparative analysis, a technique that originates in grounded theory [32]. The aim of this part of the analysis was to consolidate the list of procedures and ensure that we only retained those that were distinct from each other. Procedures and examples of their use were all systematically compared to each other; similar procedures were then merged and all procedures were classified into one of the five domains. Two authors (BC, FB) led this work and presented initial findings to the rest of the team for discussion. All authors discussed and agreed on which procedures to merge, which to retain, and how to classify them. During this process, the definitions of the five domains were iteratively modified in order to reduce ambiguity over which procedures should be classified into which domain. This resulted in a more parsimonious list of 29 procedures classified by domain. All authors discussed and agreed on the final classification of these procedures. These 29 procedures were then critically examined to ensure they were theoretically plausible means of producing placebo effects. We focused on three core psychological mechanisms [25 33-36]: response expectancy [37]; conditioning and social learning [7]; and affect, including motivation and anxiety-reduction [24 38]. However, we acknowledge that these mechanisms are difficult to tease apart [39] and that alternative mechanisms have been proposed [6] and so we erred on the side of inclusivity. Neurobiological mechanisms of placebo analgesia have been described [23 40] but a detailed consideration of how these might apply to the procedures in the taxonomy would be highly speculative and was beyond the scope of this project (for discussion of clinical applications of the neuroscience of placebo effects see [41]). Four authors (FB, BC, AG, GL) reviewed all procedures and considered the extent to which each procedure could plausibly produce placebo effects via one or more of the three core psychological mechanisms. Initial findings were shared with the remaining

For peer review only - http://bmjopen.bmj.com/site/about/guidelines.xhtml

authors and consensus was reached through discussion. Four procedures deemed very unlikely to produce placebo effects (Conveying a Neutral Therapeutic Message; Randomisation; Blinding; Deception) were excluded, leaving 25 procedures that might plausibly contribute to placebo effects. The multidisciplinary team of authors (including for example GPs, clinical and health psychologists, and complementary medicine specialists) then generated possible clinical applications of each of these 25 procedures.

Validating the Taxonomy

To ensure our taxonomy was comprehensive we surveyed leading placebo researchers (authors of major publications on placebo effects, attendees at an international symposium on placebo effects, and GPs with an interest in placebo effects). These researchers were identified from the systematic reviews and their references used to develop the taxonomy; the list of attendees at Beyond The Placebo: Biomedical Clinical and Philosophical Aspects of the Placebo Effect, held in Ascona Switzerland, August 2012; and GPs within the National Institute for Health Research School for Primary Care Research. Ethical approval for the survey was obtained from the host institution (reference: 4741). Completed electronic surveys including informed consent were received from 33 researchers (52% response rate) experienced in placebo research (M=12 years' experience, SD=9.8). Respondents were shown our draft taxonomy and asked whether, for each domain, they knew of any other procedures that could elicit placebo responses. The proportion answering yes ranged from 22% (Healthcare Setting domain) to 50% (Superficial Treatment Characteristics domain). Respondents suggested 85 additions which were screened against existing procedures and for theoretical plausibility: 80 of the suggested additions were extra details or suggested clinical applications of existing procedures; five were new and distinct plausible procedures that were added to the taxonomy, giving a final total of 30 procedures.

For peer review only

For peer review only - http://bmjopen.bmj.com/site/about/guidelines.xhtml

Because of our orientation to clinical applications, we have chosen to use clinically-oriented terminology throughout the taxonomy. However, it is important to note that when used in relation to procedures identified from the literature these terms also relate to the experimental equivalent, such that “patient” also refers to subject/participant, “practitioner” also refers to experimenter, and “intervention” also refers to experimental condition.

Analysis

The use of each of the 30 procedures in the taxonomy was assessed across all 169 studies in the review. Two authors independently rated the presence of each procedure in each study (Kappa = 0.93, discrepancies were resolved through discussion).

Results

The taxonomy defines 30 procedures that may contribute to placebo effects observed in clinical and experimental research, and classifies them into 5 domains. Table 1 presents the main taxonomy, listing and defining all 30 procedures within 5 domains. Table 2 suggests clinical applications of each procedure. Table 3 shows the frequency of use of each procedure in clinical and experimental studies, and is intended as both an approximate guide to whether the procedures derived primarily from one or other literature and as a means to highlight those procedures that are very common and very rare in the literature. Below we describe the procedures within each domain in turn.

For peer review only

For peer review only - http://bmjopen.bmj.com/site/about/guidelines.xhtml

201 Table 1. Taxonomy of Procedures Which Could Plausibly Elicit Placebo Effects in Non-Malignant Pain

Procedure Derived from Literature	Definition and Use in Research Studies
**The Patient's Beliefs and Characteristics**	
1. Select Patients Based on Treatment History.	Screen and select patients (or subgroups) against inclusion criteria related to issues such as medical/treatment history, e.g. naive to intervention being tested (not just contraindications).
2. Create Positive Expectancy.	Deliberately and explicitly suggest to patients that the intervention will be effective for them (not as part of informed consent process).
3. Reduce Negative Expectancy. [a]	The potentially negative or harmful procedures and characteristics of the treatment are deliberately minimised in information for patients.
4. Convey a Positive Therapeutic Message through Informed Consent Procedures.	Convey (verbally or in writing) a positive therapeutic message through the content of informed consent. The message might be explicit (e.g. "this intervention is usually effective in most people") or implicit (e.g. "this treatment is an antihypertensive").
5. Harness Socio-cultural Context. [a]	Tailor the intervention according to the patient's social and cultural context and history.
**The Practitioner's Beliefs and Characteristics**	
6. Practitioner Expectancy.	The person delivering the treatment expects it to be effective for the patient.
7. Practitioner's Personal Characteristics.	The practitioner's personal and/or professional characteristics (e.g. status) are modified (through selecting practitioners with different characteristics) and/or emphasised to patients.
**The Healthcare Setting**	
8. Active Recruitment.	Actively seek out and recruit patients (e.g. advertising for specific types of patients, writing personally to individual eligible patients identified through medical records).
9. Active Retention.	Make patients feel valued by attempting to keep them in a study (e.g. contact patients if they miss an appointment, incentivise attendance through monetary or non-monetary gifts).
10. Follow-up.	Assess patients after the intervention/experiment to assess long-term maintenance or changes in effects over at least 6 months.
11. Follow a Standardised Protocol.	The intervention is delivered according to a set, scientifically-derived, protocol, lending credibility to the intervention (and is therefore not individualised for each patient).
12. Ethical Oversight.	Study practices and procedures are explicitly regulated and monitored by an institutional ethics committee, lending credibility to the intervention.
13. Participating in Research.	Patients know that they are part of research and contributing to the furthering of human knowledge and/or improvement of healthcare for future patients.
14. Symptom Monitoring.	Monitor patients' symptoms using self-report measures, practitioner assessment, or objective measures

For peer review only - http://bmjopen.bmj.com/site/about/guidelines.xhtml

	repeated over time at least twice; patients are aware of the resulting measurements.
15. Enhanced Environment. [a]	The physical and interpersonal environment where the intervention is delivered is deliberately enhanced.
**Treatment Characteristics**	
16. Sham Intervention – Medication.	An inert substance is administered which is manufactured to appear identical to an active medication (e.g. sugar pill, saline IV, topical agent).
17. Sham Interventions – Physical.	A sham physical intervention is administered which is designed to appear identical to the genuine intervention (e.g. de-activated TENS, non-penetrative acupuncture needles at non-acupuncture points).
18. Sham Interventions – Attention Only.	Patients receive study-specific attention in terms of numbers of visits and time spent with study staff but no additional intervention.
19. Ineffective substances. [a]	Products unlikely to be effective or not indicated are administered (e.g. vitamins in the absence of vitamin deficiency).
20. Use Salient Side-Effects.	Potential side-effects are highlighted such that the patient can interpret them as evidence of a potent intervention.
21. Matched Treatments.	To secure blinding, placebo/sham treatments are matched to 'real' treatments (e.g. on mode of administration, dosage, frequency of administration, visual appearance, taste, smell, individual titration procedures).
22. Maximised Treatment Procedures.	The procedures and characteristics of the treatment are exaggerated, e.g. through high dose, use of colour, high frequency, large pill size, lengthy duration of intervention, ritualistic administration.
23. Conditioning.	A desired response (e.g. pain relief) is paired with an intervention stimulus (e.g. placebo cream) so that the patient associates the response with the stimulus.
**The Patient-Practitioner Interaction**	
24. The Process of Informed Consent.	The patient's formal written and/or verbal informed consent is discussed and obtained.
25. Detailed History.	A detailed personal and/or medical and/or psychosocial history is obtained from the patient.
26. Diagnosis/tests.	Additional tests, examinations, or confirmatory diagnostic procedures are undertaken to establish eligibility for the study.
27. Care.	The practitioner deliberately engages the patient with warmth, compassion and empathy.
28. Patient-Centred Communication. [a]	The practitioner adopts a style of consultation that they consider to be appropriate for a particular patient.
29. Extra Attention.	The patient receives extra attention from being in the study, for example is seen more frequently or for longer than usual.
30. Continuity of Care.	Efforts are made for the same practitioner to see the same patient at each contact.

[a] procedures added following survey of researchers.

For peer review only - http://bmjopen.bmj.com/site/about/guidelines.xhtml

Table 2. Suggested Potential Clinical Applications of Procedures to Elicit Placebo Effects in Non-Malignant Pain, Subject to Further Research

Procedure		Suggested Clinical Applications
**The Patient's Beliefs and Characteristics**		
1.	Select Patients Based on Treatment History.	Stop prescribing interventions of a type that a patient has previously not responded to (e.g. tablets); instead, prescribe a different, new, type of treatment (e.g. psychological therapy).
2.	Create Positive Expectancy.	Tell the patient the intervention is likely to be effective.<br>Elicit patients' treatment and illness beliefs and expectations, and dispel any misconceptions.<br>Empower patients to self-care.
3.	Reduce Negative Expectancy.	Limit emphasis on major potential side effects, and describe how uncommon they are.<br>Hide cessation of analgesia administration (e.g. as in Benedetti [42]), after obtaining advanced consent and ensuring patients are aware they can request additional analgesia if needed.
4.	Convey a Positive Therapeutic Message through Informed Consent Procedures.	Provide written and/or verbal information that conveys a positive therapeutic message about treatment.<br>Provide clear rationale for treatment.<br>Provide patient testimonials and supporting literature/media.
5.	Harness Socio-cultural Context.	Elicit patients' culturally embedded treatment and illness beliefs, preferences and expectations, dispelling any potentially harmful misconceptions.<br>Involve significant others in care.
**The Practitioner's Beliefs and Characteristics**		
6.	Practitioner Expectancy.	Only prescribe a treatment to patients when the practitioner expects it will be effective; communicate that expectation to patients.
7.	Practitioner's Personal Characteristics.	Honour patient preferences for particular practitioners.<br>Use indicators of expertise/high status in offices, in correspondence, and when referring to other practitioners.<br>Ensure the patient is seen by a practitioner whose views/values are congruent with the patient's.
**The Healthcare Setting**		
8.	Active Recruitment.	Actively seek out patients and invite them to attend clinic regarding a particular intervention (as opposed to waiting for patients to present).
9.	Active Retention.	Personally contact patients if they miss an appointment.<br>Use incentives to encourage patients to keep appointments.
10.	Follow-up.	Routinely invite patients to book a follow up appointment after an intervention has finished and prior to repeat prescription.<br>Encourage the patient to take responsibility for and self-manage their condition following an intervention.
11.	Follow a Standardised Protocol.	Use patient-friendly treatment protocols and share with patients where they fit in that protocol.

For peer review only - http://bmjopen.bmj.com/site/about/guidelines.xhtml

12. Ethical Oversight.	Ensure that patients understand that their treatment protocol is sanctioned by a higher authority e.g. NICE.
13. Participating in Research.	Inform patients that all outcomes and practitioner performance is audited and can contribute to improved knowledge and treatment for future patients.
14. Symptom Monitoring.	Ask patients to monitor their symptoms regularly, for example using email, phone apps, web-based systems, paper forms.<br>Assess treatment outcome.<br>Give patients feedback on symptom improvements following monitoring.
15. Enhanced Environment.	Ensure that the environment is professional, pleasant and peaceful.<br>Employ friendly and helpful support staff.
**Treatment Characteristics**	
16. Sham Intervention – Medication.	Openly prescribe sham medication.<br>With advanced prior consent, prescribe sham medication.
17. Sham Interventions – Physical.	Openly prescribe sham physical treatments.<br>With advanced prior consent, prescribe sham physical treatments.
18. Sham Interventions – Attention Only.	Increase frequency and duration of consultations.
19. Ineffective substances.	Prescribe substances that are likely not to cause harm but not clearly indicated or substances unlikely to be effective e.g. simple linctus.
20. Use Side-Effects.	Tell patients about side effects associated with positive clinical outcome.
21. Matched treatments.	Design appearance of prescribed substance (e.g. colour, packaging, taste) to match known effective treatments.
22. Maximised treatment procedures.	Within safety limits prescribe higher dose/higher frequency/larger pill.<br>Use different colour treatments.<br>Instigate ritualistic procedures patients can perform when taking medicines.<br>Maximise adherence to treatment through education, easy follow up appointments, easy repeat prescription arrangements etc.
23. Conditioning.	Prescribe highest tolerated dose first, then titrate downwards.<br>With consent, begin with active intervention, pair with a seemingly identical placebo then substitute for placebo alone.(e.g. as in Sandler[43])
**The Patient-Practitioner Interaction**	
24. The Process of Informed Consent.	Actively seek patient consent.<br>Provide treatment options and encourage the patient to choose from these options if they so desire.
25. Detailed History.	Take a detailed medical and psychosocial history/update.<br>Ensure the patient feels listened to, e.g. through non-verbal communication and/or capturing information.<br>Ask questions about the meaning of symptoms.
26. Diagnosis/tests.	Provide a definitive/confident diagnosis.<br>Examine the patient fully.
27. Care.	Allow patient adequate time to tell their story and listen to them.<br>Validate the patient’s concerns.<br>Use non-verbal techniques to convey empathy, compassion, warmth.<br>Use touch judiciously.

For peer review only - http://bmjopen.bmj.com/site/about/guidelines.xhtml

28.	Patient-Centred Communication.	Individualise consultation style according to a patient's preference e.g. collaborative vs authoritative.<br>Engage in collaborative decision-making with the patient.<br>Develop shared treatment goals that you and the patient agree on.
29.	Extra Attention.	Give extra attention to or show more interest in a patient by seeing them more frequently, having longer consultations or visiting at home.<br>Do not rush the patient.
30.	Continuity of Care.	Ensure patient is cared for by the same practitioner.<br>Read records before consultation.

Note. Suggestions for clinical applications pending research into effectiveness and ethical acceptability in clinical settings.

For peer review only

208 Table 3. Use of Procedures in Placebo Groups of Clinical and Experimental Studies

Procedure	% of studies that used each procedure: Experimental (n=58)	Clinical (n=111)
**The Patient's Beliefs and Characteristics**		
1. Select Intervention Based on Patient's Treatment History.	55%	75%
2. Create Positive Expectancy.	76%	5%
3. Reduce Negative Expectancy.	3%	0%
4. Convey a Positive Therapeutic Message through Informed Consent Procedures	43%	1%
5. Harness Socio-cultural Context.	0%	0%
**The Practitioner's Beliefs and Characteristics**		
6. Practitioner Expectancy.	0%	1%
7. Practitioner's Personal Characteristics.	9%	0%
**The Healthcare Setting**		
8. Active Recruitment.	14%	16%
9. Active Retention.	3%	2%
10. Follow-up.	2%	16%
11. Follow a Standardised Protocol.	85%	63%
12. Ethical Oversight.	78%	69%
13. Participating in Research.	86%	84%
14. Symptom Monitoring.	95%	89%
15. Enhanced Environment.	5%	0%
**Treatment Characteristics**		
16. Sham Intervention – Medication.	71%	55%
17. Sham Interventions – Physical.	33%	41%
18. Sham Interventions – Attention Only.	2%	5%
19. Ineffective substances.	0%	1%
20. Use Side-Effects.	0%	1%
21. Matched treatments.	40%	82%
22. Maximised treatment procedures.	22%	3%
23. Conditioning.	41%	0%
**The Patient-Practitioner Interaction**		
24. The Process of Informed Consent.	88%	77%
25. Detailed History.	19%	33%
26. Diagnosis/tests.	36%	41%
27. Care.	0%	1%
28. Patient-Centred Communication.	0%	0%
29. Extra Attention.	2%	63%
30. Continuity of Care.	7%	14%

209

For peer review only - http://bmjopen.bmj.com/site/about/guidelines.xhtml

The Patient’s Characteristics and Beliefs

The taxonomy specifies five procedures that act directly on the patient’s characteristics and/or beliefs in ways that might contribute to placebo effects. Procedure 1 involves selecting patients who are most likely to benefit from an intervention based on their history of similar treatments (where similarity is construed broadly at multiple levels, including appearance, modality, style, and pharmacology). For example, one might select those patients who have not experienced disappointing results from a similar intervention in the past (as the latter group might have learned to expect the intervention to fail). This procedure was commonly used by clinical trials and (to a lesser degree) experimental studies.

Procedures 2 (create positive expectancy), 3 (reduce negative expectancy), and 4 (convey a positive therapeutic message), all involve communicating with patients to encourage them to expect beneficial effects of treatment or not to expect side-effects. The majority of experimental studies in our review explicitly encouraged patients to expect treatment benefits, while very few clinical studies explicitly targeted patients’ expectations and hardly any studies attempted to minimise patients’ expectations of side-effects. Procedure 5 involves tailoring the intervention to the patient’s socio-cultural context. This approach emerged from the expert feedback and while it seems plausible and ethical to translate into clinical practice, it was not used by any of the reviewed studies.

The procedures in the patients’ beliefs and characteristics domain are thought to contribute to placebo effects primarily through altering patients’ response expectancy. Selecting patients based on treatment history and tailoring to socio-cultural context are also predicated on learning mechanisms, i.e., learned associations between treatment outcome and treatment properties.

For peer review only - http://bmjopen.bmj.com/site/about/guidelines.xhtml

There is some evidence that clinicians can give verbal suggestions to alter patients' expectations in practice and that this reduces patients' pain, particularly acute procedural pain [4 44]. As part of work to implement these procedures more widely in practice it would be important to investigate how to secure ethically valid consent for treatment. For example, clinicians might want to encourage realistically positive patient expectations while providing information about possible harms without inducing the negative expectations that could trigger nocebo effects [45 46].

The Practitioner's Characteristics and Beliefs

The two procedures in this domain are about using or modifying health care practitioners' characteristics and/or beliefs. Procedure 6 requires a practitioner to expect a treatment to benefit the patient. This might contribute to observed placebo effects in patients by influencing a practitioner's communication about the treatment and hence a patient's response expectations and/or affective response to the consultation. Only 1% of clinical studies and no experimental studies reported modifying practitioners' expectations. This procedure has received little attention in the placebo literature but clinical research in musculoskeletal settings suggests practitioners' outcome expectations can predict patients' pain outcomes [47]. One way to implement this procedure in practice would be for practitioners to communicate explicitly that they believe a treatment is effective, an approach which clearly overlaps with communication interventions designed to help doctors encourage patients to have positive expectations. Implementing Procedure 6 also depends on practitioners having relevant high quality evidence readily available and accessible and understanding this evidence as it applies to the patient.

A small proportion of studies (9% of experimental studies and no clinical studies) emphasised a practitioner's status or other characteristics (Procedure 7). For some patients a high status practitioner might elicit more confidence in the treatment (and thus higher expectations) and/or a

For peer review only - http://bmjopen.bmj.com/site/about/guidelines.xhtml

more positive affective response to the consultation [48]. Some aspects of this procedure are already part of clinical practice, for example the routine display of medical certificates in doctors' offices; others are inherent in the tools of the doctor, such as the symbolic properties of the stethoscope [49]. However, there is likely to be scope for testing their effects and augmenting their use if appropriate.

The Healthcare Setting

Procedures 8 and 9 relate to the efforts made in studies to actively recruit and retain patients respectively. Clinical and experimental studies both reportedly used these procedures sparingly (<20% for active recruitment and <5% for active retention). Such efforts may make patients feel valued and could be implemented in practice through the use of personalised communications from practitioners to encourage attendance at appointments.

Three of the eight procedures in this domain were used by over half of clinical and experimental studies and relate to basic structural features of research: following a protocol, ethical oversight, and participating in research (procedures 11 to 13). They are thought to impact patients' expectations, by emphasising the legitimacy of the intervention that is being provided and the importance of the patient's contribution to a bigger project, i.e. generating knowledge. Translating these procedures into practice could involve, for example, clinicians explicitly talking with patients about official guidance and treatment protocols that they are following.

Symptom monitoring (procedure 14) was commonly used in both clinical and experimental studies. This could be implemented in practice for example through repeatedly using patient reported outcome measures (see [50]) and might contribute to placebo effects through learning mechanisms (e.g. regular symptom monitoring acts as feedback to motivate health behaviours and/or modify

For peer review only - http://bmjopen.bmj.com/site/about/guidelines.xhtml

patients' goals). Alternatively, the mere act of asking a patient to monitor their symptoms could convey an expectation of treatment benefit, altering the meaning of a clinical interaction for the patient. Traditionally such effects of the act of measurement are dismissed as Hawthorne effects but they may also be encompassed in broader definitions of placebo effects as meaning effects [51] and could thus enhance effects in clinical practice despite being considered a nuisance in clinical research.

Very few placebo studies (5% of experimental and no clinical studies) reported enhancing the physical or interpersonal environment (procedure 15). There is a separate and distinct literature on environment modifications in health settings that might be usefully integrated with the placebo literature when developing clinical applications in this area and modelling mechanisms of action [52 53].

Treatment Characteristics

Eight procedures in the taxonomy involve modifying the characteristics of a treatment. Three involve prescribing sham interventions (sham medication – procedure 16, sham physical interventions – procedure 17, and extra attention – procedure 18) while a fourth involves prescribing a substance unlikely to be effective for the symptom in question (procedure 19). These four procedures represent variations in control conditions used in research and were frequently used by both clinical and experimental studies (with the exception of extra attention which was only used by 2-5% of studies). Such controls are thought to operate primarily via expectations, while affective pathways may also be important when extra attention from trial personnel/medical staff is involved. Of all the procedures in the taxonomy these four that represent control conditions come closest to the traditional notion of how placebos could be applied in practice. Given ethical concerns around deceptive prescribing we suggest that translational research might continue to focus on openly prescribing sham interventions including placebo pills (as in [14 15]). Other options should not

For peer review only - http://bmjopen.bmj.com/site/about/guidelines.xhtml

be dismissed entirely though: advanced consent and even waiving consent are acceptable to some patients and so it is vital for translational research to continue exploring patients', practitioners', and other stakeholders' views on the acceptability and ethics of diverse ways of prescribing placebos [54-57].

Three procedures in this domain modify the superficial (non-pharmacological or non-defining) characteristics of treatments. Procedure 20 is to highlight treatment side-effects to patients in order to encourage patients to see the treatment as potent; this procedure was very rare, used by only 1% of clinical studies. Procedure 21 was much more commonly used and involves matching the appearance of real and control treatments (used by 40% of experimental and 82% of clinical studies), in order to maintain patient blinding. This could be translated into clinical practice by designing the appearance of interventions to match patients' beliefs about what effective interventions look like. Procedure 22 involves maximising or exaggerating the superficial characteristics of treatment in order to generate larger placebo effects for example by using colour, large pill size, or ritualistic administration of medicines, manipulations which could alter the meaning of a treatment for a patient and/or enhance their expectations. 22% of experimental studies reported using this procedure and one way to translate it into practice would be to create (and test) ritualistic procedures for patients to engage in when taking medicines.

The final procedure in this domain – procedure 23, conditioning to generate placebo effects - was used commonly and exclusively by experimental studies (41%). Conditioning protocols generate placebo effects through learning mechanisms and perhaps could be implemented in practice to reduce pharmaceutical dosages, as was achieved in a pilot study in children with attention deficit disorder [43].

For peer review only

The Patient-Practitioner Interaction

The *Patient-Practitioner Interaction* domain incorporates seven procedures related to the interpersonal relationship or interactions between a patient and their health care practitioner. These procedures are thought to operate primarily through affective mechanisms such as reduced anxiety after telling one's story and being listened to with empathy and acknowledged, although more cognitive pathways via expectations are also plausible[58]. Three procedures are about specific processes that can occur during consultations – obtaining informed consent (procedure 24), taking a detailed history (procedure 25), and performing additional diagnoses or tests (procedure 26). Arguably these procedures indicate to the patient that the practitioner respects them, is interested in their perspective, and is thorough in their diagnosis. They occur in both clinical and experimental research settings and could be relatively directly translated into practice or optimised if already used.

Two procedures are about the way in which the practitioner engages with the patient: communicating care (procedure 27), and patient-centred communication (procedure 28). These procedures were surprisingly very rarely described in the studies included in our review, although recently the nocebo effects of *not* validating a patient's experiences have been shown to be particularly potent [59]. There is of course a distinct and large literature on doctor-patient communication and fruitful dialogue is beginning to bridge these fields [60].

The final two procedures in this domain refer to more structural aspects of consultations: extra attention (procedure 29, i.e. longer or more frequent appointments) and continuity of care (procedure 30). 63% of clinical studies used extra attention while a small proportion of clinical (14%) and experimental (7%) studies reported providing continuity of care. Directly implementing these procedures in practice might be challenging given ever increasing constraints on healthcare resources and drives to reduce cost.

For peer review only - http://bmjopen.bmj.com/site/about/guidelines.xhtml

Discussion

The taxonomy names and describes 30 procedures that may contribute to placebo effects in experimental and clinical studies and classifies them into five domains. It includes 60 theoretically plausible clinical applications, subject to further research on their effectiveness and ethical acceptability in practice. Some of the clinical applications derived from the placebo literature have already been investigated in their own right under other auspices, highlighting the need for the burgeoning translational science of placebo effects to be broad-ranging and interdisciplinary.

We have used rigorous systematic review and qualitative analytic methods complemented by a survey to develop the taxonomy. Investigators often combine multiple techniques in any one 'placebo' (e.g. Create Positive Expectancy + Detailed History + Symptom Monitoring) making it beyond the scope of this project to unpack the effectiveness of individual techniques. Procedures did not always fit neatly into single domains. For example, "Screen for Treatment History" was used to select patients for studies of specific treatments (and was thus placed in the Patient's Beliefs and Characteristics domain), but its clinical application involves selecting a treatment for a specific patient and so could be considered a Treatment Characteristic. Conceptually we would expect interactions between these domains; for example, some procedures categorised in other domains probably operate through causal pathways involving patients' beliefs as proximal determinants of placebo effects [61]. We feel the benefits of having a hierarchical structure (modifiable as the taxonomy is refined with use) outweigh the difficulties inherent in classification. We could have used many published reviews of placebo studies in non-malignant pain to identify original studies to review. Selecting seven such reviews means not using others, thus we might have missed original studies that would have suggested additional procedures. Surveying leading researchers and incorporating their suggestions somewhat mitigates this limitation. Our sample of researchers was

For peer review only - http://bmjopen.bmj.com/site/about/guidelines.xhtml

intended to be purposive, in that we wanted to obtain the views of leading researchers in the field. By using multiple means of identifying such individuals internationally we feel we have achieved this. The reviews that we selected as the source of our papers and the papers themselves are now somewhat old examples of the literature; and our choice to exclude papers published before 1983 was arguably somewhat arbitrary. Future work should review very recent papers and iteratively improve the taxonomy accordingly.

This review extends previous work by Di Blasi et al. [31], building on their five domains to systematically develop a detailed taxonomy. We provide a new overarching framework that avoids the controversial and limited distinction between pure and impure placebos [62 63] and integrates ideas from the rich clinical and experimental literatures on placebo effects in non-malignant pain. Many of the components we have identified are likely to be important in other placebo-responsive conditions including depression [64], irritable bowel syndrome [65 66], and insomnia [67]. This taxonomy can guide two important and related applied research agendas: 1) to understand the components of placebo effects in clinical settings [48 68-71] and 2) to ethically harness evidence-based placebo effects to benefit patients [14 15 72]. We hope future studies might draw on the taxonomy to fully describe their methods and develop new applications, thus facilitating future systematic reviews and the development of a systematic and theory driven cumulative evidence-base in this complex field.

The taxonomy identifies and classifies procedures that may contribute to placebo effects in clinical trials and experiments, providing an overarching framework for individual components. However, we do not suggest that every technique in this taxonomy will produce a placebo effect in every patient and we do not know from this project which techniques are more effective or how they might be combined to form ethically acceptable and effective complex interventions. This taxonomy

For peer review only - http://bmjopen.bmj.com/site/about/guidelines.xhtml

provides the first attempt at a necessary conceptual tool to facilitate future research on these questions. For example, systematic reviews could use the taxonomy to code procedures in original studies, using this information in meta-regression analysis to examine the contribution of different procedures to placebo effects [73]. New clinical trials and experiments could extend existing work by systematically examining and comparing the effects and ethical acceptability of different procedures in the taxonomy, building a cumulative evidence-base that has real pragmatic applicability to clinical practice. Some of the suggested clinical applications have been investigated more extensively in other literatures, in particular doctor-patient communication and the healthcare environment. This emphasises the need for a multi-disciplinary approach to the translation of placebo research into practice. One fruitful way forward would be to draw on placebo theories to develop and test more mechanistic models of complex interventions intended to alter the context of healthcare encounters.

Placebo recipients in clinical trials and experiments are exposed to a large number and variety of procedures, many of which might contribute to placebo effects. Researchers seeking to develop a translational science of placebo effects are thus faced with myriad possibilities. We have systematically identified and defined these procedures, classified them into five domains, and suggested possible clinical applications. The resulting taxonomy is presented as a preliminary but detailed and systematic guide for future research, which should in turn further refine the taxonomy. Ultimately we hope to better conceptualise investigations of clinical applications of placebo effects in order to maximise opportunities for patient benefit.

For peer review only - http://bmjopen.bmj.com/site/about/guidelines.xhtml

Funding

The project “Creating a Taxonomy to Harness the Placebo effect in UK primary care” was funded by the National Institute of Health Research (NIHR) School for Primary Care Research (SPCR) (project number 161). This paper presents independent research funded by the National Institute of Health Research (NIHR). The views expressed are those of the author(s) and not necessarily those of the NHS, the NIHR or the Department of Health. Additional funding for BC was provided by Solent NHS Trust. The funders had no role in design and conduct of the study; collection, management, analysis, and interpretation of the data; preparation, review, or approval of the manuscript; and decision to submit the manuscript for publication.

Acknowledgements

We would like to acknowledge the contribution of all of the researchers who shared their views in the survey, including Przemyslaw Babel PhD, Luana Colloca MD PhD, Professor Michael Doherty, Vanda Faria PhD, Professor Magne Arve Flaten PhD, Sarah Goldingay PhD, John Hughes PhD, Professor Dr Robert Juette, Irving Kirsch PhD, Karin Meissner PD Dr. med. Habil., Daniel E Moerman PhD, Meike Shedden Mora PhD, Donald D. Price PhD, Professor Dr Dr Harald Walach. We thank Professor Ted Kaptchuk for comments on an earlier draft of this manuscript.

Competing Interests

All authors have completed the ICMJE uniform disclosure form at http://www.icmje.org/coi_disclosure.pdf and declare: no support from any organisation for the submitted work; no financial relationships with any organisations that might have an interest in the

For peer review only

For peer review only - http://bmjopen.bmj.com/site/about/guidelines.xhtml

submitted work in the previous three years, no other relationships or activities that could appear to have influenced the submitted work.

Exclusive Licence

The Corresponding Author has the right to grant on behalf of all authors and does grant on behalf of all authors, a worldwide licence (http://www.bmj.com/sites/default/files/BMJ%20Author%20Licence%20March%202013.doc) to the Publishers and its licensees in perpetuity, in all forms, formats and media (whether known now or created in the future), to i) publish, reproduce, distribute, display and store the Contribution, ii) translate the Contribution into other languages, create adaptations, reprints, include within collections and create summaries, extracts and/or, abstracts of the Contribution and convert or allow conversion into any format including without limitation audio, iii) create any other derivative work(s) based in whole or part on the on the Contribution, iv) to exploit all subsidiary rights to exploit all subsidiary rights that currently exist or as may exist in the future in the Contribution, v) the inclusion of electronic links from the Contribution to third party material where-ever it may be located; and, vi) licence any third party to do any or all of the above.

Contributorship Statement

FB designed and led the study, drafted the manuscript, and is guarantor. FB, GL, AWAG, HE, and PL secured funding for the project. FB designed the study with input and revisions from GL, BC, AWAG, HE, and PL. BC led data collection and analysis with additional data collection and analysis by MH and DS. All authors contributed to data interpretation. FB drafted the manuscript and all authors revised it for important intellectual content. All authors had full access to all of the data in the study and can take responsibility for the integrity of the data and the accuracy of the data analysis.

Data Sharing Statement

No unpublished data available.

For peer review only - http://bmjopen.bmj.com/site/about/guidelines.xhtml

References

1. Zhang W, Robertson J, Jones AC, et al. The placebo effect and its determinants in osteoarthritis: meta-analysis of randomised controlled trials. Ann Rheum Dis 2008;**67**(12):1716-23.
2. Hrobjartsson A, Gotzsche PC. Placebo interventions for all clinical conditions. Cochrane Database of Systematic Reviews 2010;**1**:Art. No.: CD003974-DOI: 10.1002/14651858.CD003974.pub3.
3. Vase L, Riley JL, Price DD. A comparison of placebo effects in clinical analgesic trials versus studies of placebo analgesia. Pain 2002;**99**(3):443-52.
4. Peerdeman KJ, van Laarhoven AIM, Keij SM, et al. Relieving patients' pain with expectation interventions: a meta-analysis. Pain 2016;**157**(6):1179-91.
5. Brody H. The placebo response. J Fam Pract 2000;**49**:649-54.
6. Moerman DE, Jonas WB. Deconstructing the placebo effect and finding the meaning response. Ann Intern Med 2002;**136**(6):471-76.
7. Colloca L, Miller FG. How placebo responses are formed: a learning perspective. Philosophical Transactions of the Royal Society B: Biological Sciences 2011;**366**(1572):1859-69.
8. Howick J, Friedemann C, Tsakok M, et al. Are Treatments More Effective than Placebos? A Systematic Review and Meta-Analysis. PLoS One 2013;**8**(5):e62599.
9. Doherty M, Dieppe P. The "placebo" response in osteoarthritis and its implications for clinical practice. Osteoarthritis Cartilage 2009;**17**(10):1255-62.
10. Lichtenberg P, Heresco-Levy U, Nitzan U. The ethics of the placebo in clinical practice. J Med Ethics 2004;**30**:551-54.
11. Colloca L, Miller FG. Harnessing the placebo effect: the need for translational research. Philosophical Transactions of the Royal Society B: Biological Sciences 2011;**366**(1572):1922-30.
12. Benedetti F, Pollo A, Lopiano L, et al. Conscious expectation and unconscious conditioning in analgesic, motor, and hormonal placebo/nocebo responses. The Journal of Neuroscience 2003;**23**(10):4315-23.
13. Bingel U, Wanigasekera V, Wiech K, et al. The Effect of Treatment Expectation on Drug Efficacy: Imaging the Analgesic Benefit of the Opioid Remifentanil. Science Translational Medicine 2011;**3**(70):70ra14-70ra14.
14. Kaptchuk TJ, Friedlander E, Kelley JM, et al. Placebos without deception: A randomized controlled trial in Irritable Bowel Syndrome. PLoS One 2010;**5**(12):e15591.
15. Kelley JM, Kaptchuk TJ, Cusin C, et al. Open-label placebo for major depressive disorder: a pilot randomized-controlled trial. Psychother Psychosom 2012;**81**:312-14.
16. Blease C, Colloca L, Kaptchuk TJ. Are open-Label Placebos Ethical? Informed Consent and Ethical Equivocations. Bioethics 2016;**30**(6):407-14.
17. Barnhill A, Miller FG. The ethics of placebo treatments in clinical practice: a reply to Glackin. J Med Ethics 2015.
18. Enck P, Bingel U, Schedlowski M, et al. The placebo response in medicine: minimize, maximize or personalize? Nat Rev Drug Discov 2013;**12**(3):191-204.
19. Linde K, F„ssler M, Meissner K. Placebo interventions, placebo effects and clinical practice. Philosophical Transactions of the Royal Society B: Biological Sciences 2011;**366**(1572):1905-12.
20. Klinger R, Colloca L, Bingel U, et al. Placebo analgesia: Clinical applications. Pain 2014;**155**(6):1055-58.
21. Kaptchuk TJ, Miller FG. Placebo Effects in Medicine. N Engl J Med 2015;**373**(1):8-9.
22. Dowell D, Haegerich TM, Chou R. CDc guideline for prescribing opioids for chronic pain—united states, 2016. JAMA 2016;**315**(15):1624-45.
23. Colloca L, Klinger R, Flor H, et al. Placebo analgesia: psychological and neurobiological mechanisms. Pain 2013;**154**(4):511-4.
24. Price DD, Finniss DG, Benedetti F. A comprehensive review of the placebo effect: Recent advances and current thought. Annu Rev Psychol 2008;**59**:565-90.

For peer review only - http://bmjopen.bmj.com/site/about/guidelines.xhtml

25. Finniss DG, Kaptchuk TJ, Miller FG, et al. Biological, clinical, and ethical advances of placebo effects. Lancet 2010;**375**(9715):686-95.
26. Manchikanti L, Giordano J, Fellows B, et al. Placebo and nocebo in interventional pain management: A friend or a foe - or simply foes? Pain Physician 2011;**14**:E157-E75.
27. Puhl AA, Reinhart CJ, Rok ER, et al. An examination of the observed placebo effect associated with the treatment of low back pain - a systematic review. Pain Research & Management 2011;**16**(1):45-52.
28. Faria V, Fredrikson M, Furmark T. Imaging the placebo response: A neurofunctional review. Eur Neuropsychopharmacol 2008;**18**(7):473-85.
29. Vase L, Petersen GL, Riley Iii JL, et al. Factors contributing to large analgesic effects in placebo mechanism studies conducted between 2002 and 2007. Pain 2009;**145**(1-2):36-44.
30. Kirsch I. Placebo psychotherapy: Synonym or oxymoron? J Clin Psychol 2005;**61**(7):791-803.
31. Di Blasi Z, Harkness E, Ernst E, et al. Influence of context effects on health outcomes: a systematic review. Lancet 2001;**357**:757-62.
32. Strauss A, Corbin J. *Basics of Qualitative Research Techniques and Procedures for Developing Grounded Theory*. Thousand Oaks: Sage, 1998.
33. Meissner K, Kohls N, Colloca L. Introduction to placebo effects in medicine: mechanisms and clinical implications. Philosophical Transactions of the Royal Society B: Biological Sciences 2011;**366**(1572):1783-89.
34. Price DD, Chung SK, Robinson ME. Conditioning, expectation, and desire for relief in placebo analgesia. Seminars in Pain Medicine 2005;**3**(1):15-21.
35. Benedetti F, Amanzio M. The placebo response: How words and rituals change the patient's brain. Patient Educ Couns 2011;**84**(3):413-19.
36. Goffaux P, L,onard G, Marchand S, et al. Placebo analgesia. In: Beaulieu P, Lussier D, Porreca F, et al., eds. Pharmacology of Pain. Seattle, WA: IASP Press, 2010:451-73.
37. Kirsch I. Response expectancy theory and application: A decennial review. Appl Prev Psychol 1997;**6**(2):69-79.
38. Hyland ME, Whalley B. Motivational concordance: An important mechanism in self-help therapeutic rituals involving inert (placebo) substances. J Psychosom Res 2008;**65**(5):405-13.
39. Stewart-Williams S, Podd J. The placebo-effect: Dissolving the expectancy versus conditioning debate. Psychol Bull 2004;**130** (2):324-40.
40. Benedetti F. Placebo and the New Physiology of the Doctor-Patient Relationship. Physiol Rev 2013;**93**(3):1207-46.
41. Jubb J, Bensing JM. The sweetest pill to swallow: How patient neurobiology can be harnessed to maximise placebo effects. Neurosci Biobehav Rev 2013;**37**(10, Part 2):2709-20.
42. Benedetti F, Maggi G, Lopiano L, et al. Open versus hidden medical treatments: The patient's knowledge about a therapy affects the therapy outcome. Prevention & Treatment 2003;**6**(1):No.
43. Sandler AD, Bodfish JW. Open-label use of placebos in the treatment of ADHD: a pilot study. Child Care Health Dev 2008;**34**(1):104-10.
44. Mistiaen P, van Osch M, van Vliet L, et al. The effect of patient-practitioner communication on pain: a systematic review. Eur J Pain 2015.
45. Planès S, Villier C, Mallaret M. The nocebo effect of drugs. Pharmacology Research & Perspectives 2016;**4**(2):n/a-n/a.
46. Colloca L, Finniss D. Nocebo effects, patient-clinician communication, and therapeutic outcomes. JAMA 2012;**307**(6):567-8.
47. Witt CM, Martins F, Willich SN, et al. Can I help you? Physicians' expectations as predictor for treatment outcome. European Journal of Pain 2012;**16**(10):1455-66.
48. White P, Bishop FL, Prescott P, et al. Practice, practitioner or placebo? A multifactorial, mixed methods randomized controlled trial of acupuncture. Pain 2012;**153**:455-62.

For peer review only - http://bmjopen.bmj.com/site/about/guidelines.xhtml

49. Rice T. ‘The hallmark of a doctor’: the stethoscope and the making of medical identity. Journal of Material Culture 2010;**15**(3):287-301.
50. Snyder CF, Aaronson NK, Choucair AK, et al. Implementing patient-reported outcomes assessment in clinical practice: a review of the options and considerations. Qual Life Res 2012;**21**(8):1305-14.
51. Benedetti F, Carlino E, Piedimonte A. Increasing uncertainty in CNS clinical trials: the role of placebo, nocebo, and Hawthorne effects. The Lancet Neurology;**15**(7):736-47.
52. Drahota A, Ward D, Mackenzie H, et al. Sensory environment on health-related outcomes of hospital patients (Review). Cochrane Database of Systematic Reviews 2012;**3**:Art. No.: CD005315. DOI: 10.1002/14651858.CD005315.pub2.
53. Dijkstra K, Pieterse M, Pruyn A. Physical environmental stimuli that turn healthcare facilities into healing environments through psychologically mediated effects: systematic review. J Adv Nurs 2006;**56**(2):166-81.
54. Feffer K, Lichtenberg P, Becker G, et al. A comparative study with depressed patients on the acceptability of placebo use. Gen Hosp Psychiatry 2016;**41**:53-6.
55. Ortiz R, Chandros Hull S, Colloca L. Patient attitudes about the clinical use of placebo: qualitative perspectives from a telephone survey. BMJ Open 2016;**6**(4).
56. Bishop FL, Howick J, Heneghan C, et al. Placebo use in the United Kingdom: a qualitative study exploring GPs' views on placebo effects in clinical practice. Fam Pract 2014;**Advance Access**:1-7. doi:10.1093/fampra/cmu016.
57. Bishop FL, Aizlewood L, Adams AEM. When and Why Placebo-Prescribing Is Acceptable and Unacceptable: A Focus Group Study of Patients' Views. PLoS One 2014;**9**(7):e101822.
58. Street, Jr., Makoul G, Arora NK, et al. How does communication heal? Pathways linking clinician-patient communication to health outcomes. Patient Educ Couns 2009;**74**(3):295-301.
59. Greville-Harris M, Dieppe P. Bad Is More Powerful than Good: The Nocebo Response in Medical Consultations. The American Journal of Medicine 2015;**128**(2):126-29.
60. Bensing JM, Verheul W. The silent healer: The role of communication in placebo effects. Patient Educ Couns 2010;**80**(3):293-99.
61. Kirsch I. Conditioning, expectancy, and the placebo effect: Comment on Stewart-Williams and Podd (2004). Psychol Bull 2004;**130**(2):341-43.
62. Fassler M, Gnadinger M, Rosemann T, et al. Use of placebo interventions among Swiss primary care providers. BMC Health Serv Res 2009;**9**(1):144.
63. Howick J, Bishop FL, Heneghan C, et al. Placebo use in the United Kingdom: results from a national survey of primary care practitioners. PLoS One 2013;**8**(3):e58247. doi:10.1371/journal.pone.0058247.
64. Kirsch I, Sapirstein G. Listening to prozac but hearing placebo: A meta-analysis of antidepressant medication. Prevention & Treatment 1998;**1** Article 0002a, posted June 26, 1998.
65. Patel SM, Stason WB, Legedza A, et al. The placebo effect in irritable bowel syndrome trials: a meta-analysis. Neurogastroenterol Motil 2005;**17**(3):332-40.
66. Ford AC, Moayyedi P. Meta-analysis: factors affecting placebo response rate in the irritable bowel syndrome. Aliment Pharmacol Ther 2010;**32**(2):144-58.
67. Bélanger L, Vallières A, Ivers H, et al. Meta-analysis of sleep changes in control groups of insomnia treatment trials. J Sleep Res 2007;**16**(1):77-84.
68. Kaptchuk TJ, Kelley JM, Conboy LA, et al. Components of placebo effect: randomised controlled trial in patients with irritable bowel syndrome. Br Med J 2008;**336**:999-1003.
69. Suarez-Almazor ME, Looney C, Liu Y, et al. A randomized controlled trial of acupuncture for osteoarthritis of the knee: Effects of patient-provider communication. Arthritis Care Res 2010;**62**(9):1229-36.
70. Kaptchuk TJ, Stason WB, Davis RB, et al. Sham device v inert pill: randomised controlled trial of two placebo treatments. Br Med J 2006;**332**(7538):391-94.

For peer review only - http://bmjopen.bmj.com/site/about/guidelines.xhtml

624 71. Vase L, Baram S, Takakura N, et al. Specifying the nonspecific components of acupuncture analgesia. Pain 2013;**154**(9):1659-67.

626 72. Sandler A, Glesne C, Geller G. Children's and parents' perspectives on open-label use of placebos in the treatment of ADHD. Child Care Health Dev 2008;**34**(1):111-20.

628 73. Michie S, Abraham C, Whittington C, et al. Effective techniques in healthy eating and physical activity interventions: a meta-regression. Health Psychol 2009;**28**(6):690-701.

630

For peer review only

For peer review only - http://bmjopen.bmj.com/site/about/guidelines.xhtml

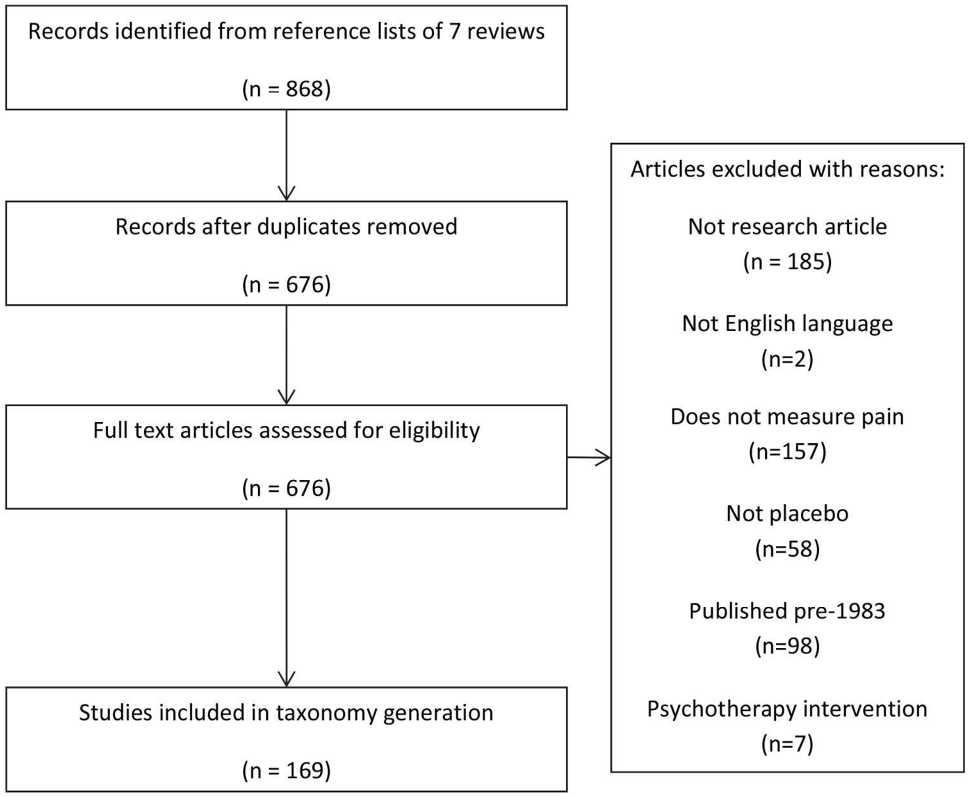

Flowchart Showing Identification of Studies

155x127mm (300 x 300 DPI)

For peer review only

Supplementary Digital Content

List of Included Studies

[1-169]

[1] Alfano AP, Taylor AG, Foresman PA, Dunk PR, McConnell GG, Gillies GT. Static magnetic fields for treatment of fibromyalgia: A randomized controlled trial. . J Altern Complement Med 2001;7(1):53.

[2] Alford JW, Fadale PD. Evaluation of postoperative bupivacaine infusion for pain management after anterior cruciate ligament reconstruction. Arthroscopy-the Journal of Arthroscopic and Related Surgery 2003;19(8):855.

[3] Amanzio M, Benedetti F. Neuropharmacological Dissection of Placebo Analgesia: Expectation-Activated Opioid Systems versus Conditioning-Activated Specific Subsystems. The Journal of Neuroscience 1999;19(1):484-494.

[4] Amanzio M, Pollo A, Maggi G, Benedetti F. Response variability to analgesics: a role for non-specific activation of endogenous opioids. Pain 2001;90(3):205-215.

[5] Amlie E, Weber H, Holme I. Treatment of Acute Low-back Pain with Piroxicam: Results of a Double-blind Placebo-controlled Trial. Spine 1987;12(5):473-476.

[6] Andersen AN, Damm P, Tabor A, Pedersen IM, Harring M. Prevention of Breast Pain and Milk Secretion with Bromocriptine After Second-Trimester Abortion. Acta Obstet Gynecol Scand 1990;69(3):235-238.

[7] Aslaksen PM, Flaten MA. The Roles of Physiological and Subjective Stress in the Effectiveness of a Placebo on Experimentally Induced Pain. Psychosom Med 2008;70(7):811-818.

[8] Atkinson JH, Slater MA, Williams RA, Zisook S, Patterson TL, Grant I, Wahlgren DR, Abramson I, Garfin SR. A placebo-controlled randomized clinical trial of nortriptyline for chronic low back pain. Pain 1998;76(3):287-296.

[9] Bannwarth B, Allaert FA, Avouac B, Rossignol M, Rozenberg S, Valat JP. A randomized, double-blind, placebo controlled triphosphate in study of oral adenosine subacute low back pain. J Rheumatol 2005;32(6):1114-1117.

[10] Basford JR, Sheffield CG, Harmsen WS. Laser therapy: a randomized, controlled trial of the effects of low-intensity Nd:YAG laser irradiation on musculoskeletal back pain. Arch Phys Med Rehabil 1999;80(6):647-652.

[11] Basmajian JV. Acute back pain and spasm. A controlled multicenter trial of combined analgesic and antispasm agents. Spine (Phila Pa 1976) 1989;14(4):438-439.

[12] Benedetti F. The opposite effects of the opiate antagonist naloxone and the cholecystokinin antagonist proglumide on placebo analgesia. Pain 1996;64(3):535-543.

[13] Benedetti F, Amanzio M, Baldi S, Casadio C, Cavallo A, Mancuso M, Ruffini E, Oliaro A, Maggi G. The specific effects of prior opioid exposure on placebo analgesia and placebo respiratory depression. Pain 1998;75(2-3):313-319.

[14] Benedetti F, Amanzio M, Casadio C, Oliaro A, Maggi G. Blockade of nocebo hyperalgesia by the cholecystokinin antagonist proglumide. Pain 1997;71(2):135-140.

[15] Benedetti F, Amanzio M, Maggi G. Potentiation of placebo analgesia by proglumide. The Lancet 1995;346(8984):1231.

[16] Benedetti F, Amanzio M, Vighetti S, Asteggiano G. The Biochemical and Neuroendocrine Bases of the Hyperalgesic Nocebo Effect. The Journal of Neuroscience 2006;26(46):12014-12022.

[17] Benedetti F, Arduino C, Amanzio M. Somatotopic activation of opioid systems by target-directed expectations of analgesia. J Neurosci 1999;19(9):3639-3648.

[18] Benedetti F, Arduino C, Costa S, Vighetti S, Tarenzi L, Rainero I, Asteggiano G. Loss of expectation-related mechanisms in Alzheimer's disease makes analgesic therapies less effective. Pain 2006;121(1):133-144.

For peer review only

[19] Benedetti F, Pollo A, Lopiano L, Lanotte M, Vighetti S, Rainero I. Conscious expectation and unconscious conditioning in analgesic, motor, and hormonal placebo/nocebo responses. The Journal of Neuroscience 2003;23(10):4315-4323.
[20] Benedetti FMD, Amanzio MMS, Casadio CMD, Cavallo AMD, Cianci RMD, Giobbe RMD, Mancuso MMD, Ruffini EMD, Maggi GMD. Control of Postoperative Pain by Transcutaneous Electrical Nerve Stimulation After Thoracic Operations. The Annals of Thoracic Surgery 1997;63(3):773-776.
[21] Berry H, Hutchinson DR. A multicentre placebo-controlled study in general practice to evaluate the efficacy and safety of tizanidine in acute low-back pain. J Int Med Res 1988;16(2):75-82.
[22] Bingel U, Lorenz J, Schoell E, Weiller C, Büchel C. Mechanisms of placebo analgesia: rACC recruitment of a subcortical antinociceptive network. Pain 2006;120(1):8-15.
[23] Birbara CA, Puopolo AD, Munoz DR, Sheldon EA, Mangione A, Bohidar NR, Geba GP. Treatment of chronic low back pain with etoricoxib, a new cyclo-oxygenase-2 selective inhibitor: improvement in pain and disability--a randomized, placebo-controlled, 3-month trial. The journal of pain : official journal of the American Pain Society 2003;4(6):307-315.
[24] Biro P, Meier T, Cummins AS. Comparison of topical anaesthesia methods for venous cannulation in adults. Eur J Pain 1997;1(1):37-42.
[25] Blanchard EB, Appelbaum KA, Radnitz CL, Michultka D, Morrill B, Kirsch C, Hillhouse J, Evans DD, Guarnieri P, Attanasio V, et al. Placebo-controlled evaluation of abbreviated progressive muscle relaxation and of relaxation combined with cognitive therapy in the treatment of tension headache. J Consult Clin Psychol 1990;58(2):210-215.
[26] Blanchard EB, Appelbaum KA, Radnitz CL, Morrill B, Michultka D, Kirsch C, Guarnieri P, Hillhouse J, Evans DD, Jaccard J, et al. A controlled evaluation of thermal biofeedback and thermal biofeedback combined with cognitive therapy in the treatment of vascular headache. J Consult Clin Psychol 1990;58(2):216-224.
[27] Boissel JP, Philippon AM, Gauthier E, Schbath J, Destors JM. Time course of long-term placebo therapy effects in angina pectoris. Eur Heart J 1986;7(12):1030-1036.
[28] Bova JG, Bhattacharjee N, Jurdi R, Bennett WF. Comparison of no medication, placebo, and hyoscyamine for reducing pain during a barium enema. Am J Roentgenol 1999;172(5):1285-1287.
[29] Brinkhaus B, Witt CM, Jena S, Linde K, Streng A, Wagenpfeil S, Irnich D, Walther HU, Melchart D, Willich SN. Acupuncture in patients with chronic low back pain - A randomized controlled trial. Arch Intern Med 2006;166(4):450-457.
[30] Camilleri M, Northcutt AR, Kong S, Dukes GE, McSorley D, Mangel AW. Efficacy and safety of alosetron in women with irritable bowel syndrome: a randomised, placebo-controlled trial. The Lancet 2000;355(9209):1035-1040.
[31] Carbajal R, Chauvet X, Couderc S, Olivier-Martin M. Randomised trial of analgesic effects of sucrose, glucose, and pacifiers in term neonates. BMJ 1999;319(7222):1393-1397.
[32] Carette S, Leclaire R, Marcoux S, Morin F, Blaise GA, St-Pierre A, Truchon R, Parent F, Levesque J, Bergeron V, Montminy P, Blanchette C. Epidural corticosteroid injections for sciatica due to herniated nucleus pulposus. N Engl J Med 1997;336(23):1634-1640.
[33] Charron J, Rainville P, Marchand S. Direct comparison of placebo effects on clinical and experimental pain. Clin J Pain 2006;22(2):204-211.
[34] Cherkin DC, Sherman KJ, Avins AL, et al. A randomized trial comparing acupuncture, simulated acupuncture, and usual care for chronic low back pain. Arch Intern Med 2009;169(9):858-866.
[35] Chrubasik S, Eisenberg E, Balan E, Weinberger T, Luzzati R, Conradt C. Treatment of low back pain exacerbations with willow bark extract: a randomized double-blind study. Am J Med 2000;109(1):9-14.

For peer review only

[36] Chrubasik S, Junck H, Breitschwerdt H, Conradt C, Zappe H. Effectiveness of Harpagophytum extract WS 1531 in the treatment of exacerbation of low back pain: a randomized, placebo-controlled, double-blind study. Eur J Anaesthesiol 1999;16(2):118-129.
[37] Chung SK, Price DD, Verne GN, Robinson ME. Revelation of a personal placebo response: Its effects on mood, attitudes and future placebo responding. Pain 2007;132(3):281-288.
[38] Classen W, Feingold E, Netter P. Influence of sensory suggestibility on treatment outcome in headache patients. Neuropsychobiology 1983;10(1):44-47.
[39] Coats TL, Borenstein DG, Nangia NK, Brown MT. Effects of valdecoxib in the treatment of chronic low back pain: results of a randomized, placebo-controlled trial. Clin Ther 2004;26(8):1249-1260.
[40] Colloca L, Benedetti F. How prior experience shapes placebo analgesia. Pain 2006;124(1-2):126-133.
[41] Colloca L, Benedetti F. Placebo analgesia induced by social observational learning. Pain 2009;144(1-2):28-34.
[42] Colloca L, Petrovic P, Wager TD, Ingvar M, Benedetti F. How the number of learning trials affects placebo and nocebo responses. Pain 2010;151(2):430-439.
[43] Colloca L, Sigaudo M, Benedetti F. The role of learning in nocebo and placebo effects. Pain 2008;136(1-2):211-218.
[44] Conn IG, Marshall AH, Yadav SN, Daly JC, Jaffer M. Transcutaneous electrical nerve stimulation following appendicectomy: the placebo effect. Ann R Coll Surg Engl 1986;68(4):191-192.
[45] Corson SL, Batzer FR, Gocial B, Kelly M, Gutmann JN, Go KJ, English ME. Is paracervical block anesthesia for oocyte retrieval effective? Fertil Steril 1994;62(1):133-136.
[46] Costello M, Ramundo M, Christopher NC, Powell KR. Ethyl Vinyl Chloride Vapocoolant Spray Fails to Decrease Pain Associated with Intravenous Cannulation in Children. Clin Pediatr (Phila) 2006;45(7):628-632.
[47] Coyne PJ, MacMurren M, Izzo T, Kramer T. Transcutaneous electrical nerve stimulator for procedural pain associated with intravenous needlesticks. J Intraven Nurs 1995;18(5):263-267.
[48] Dapas F, Hartman SF, Martinez L, Northrup BE, Nussdorf RT, Silberman HM, Gross H. Baclofen for the treatment of acute low-back syndrome. A double-blind comparison with placebo. Spine (Phila Pa 1976) 1985;10(4):345-349.
[49] De Pascalis V, Chiaradia C, Carotenuto E. The contribution of suggestibility and expectation to placebo analgesia phenomenon in an experimental setting. Pain 2002;96(3):393-402.
[50] deCharms RC, Maeda F, Glover GH, Ludlow D, Pauly JM, Soneji D, Gabrieli JDE, Mackey SC. Control over brain activation and pain learned by using real-time functional MRI. Proc Natl Acad Sci U S A 2005;102(51):18626-18631.
[51] Defrin R, Ariel E, Peretz C. Segmental noxious versus innocuous electrical stimulation for chronic pain relief and the effect of fading sensation during treatment. Pain 2005;115(1):152-160.
[52] Dickens C, Jayson M, Sutton C, Creed F. The relationship between pain and depression in a trial using paroxetine in sufferers of chronic low back pain. Psychosomatics 2000;41(6):490-499.
[53] Ditto B, France CR. The effects of applied tension on symptoms in French-speaking blood donors: a randomized trial. Health Psychol 2006;25(3):433-437.
[54] Ditto B, France CR, Lavoie P, Roussos M, Adler PS. Reducing reactions to blood donation with applied muscle tension: a randomized controlled trial. Transfusion (Paris) 2003;43(9):1269-1275.
[55] Dreiser RL, Marty M, Ionescu E, Gold M, Liu JH. Relief of acute low back pain with diclofenac-K 12.5 mg tablets: a flexible dose, ibuprofen 200 mg and placebo-controlled clinical trial. Int J Clin Pharmacol Ther 2003;41(9):375-385.
[56] Erdogmus CB, Resch KL, Sabitzer R, Muller H, Nuhr M, Schoggl A, Posch M, Osterode W, Ungersbock K, Ebenbichler GR. Physiotherapy-based rehabilitation following disc herniation

For peer review only

operation: results of a randomized clinical trial. Spine (Phila Pa 1976) 2007;32(19):2041-2049.
[57] Faas A, Chavannes AW, van Eijk JT, Gubbels JW. A randomized, placebo-controlled trial of exercise therapy in patients with acute low back pain. Spine (Phila Pa 1976) 1993;18(11):1388-1395.
[58] Fanti L, Gemma M, Passaretti S, Guslandi M, Testoni PA, Casati A, Torri G. Electroacupuncture Analgesia for Colonoscopy: A Prospective, Randomized, Placebo-Controlled Study. Am J Gastroenterol 2003;98(2):312-316.
[59] Forster EL, Kramer JF, Lucy SD, Scudds RA, Novick RJ. EFfect of tens on pain, medications, and pulmonary function following coronary artery bypass graft surgery. CHEST Journal 1994;106(5):1343-1348.
[60] Foster KA, Liskin J, Cen S, Abbott A, Armisen V, Globe D, Knox L, Mitchell M, Shtir C, Azen S. The Trager approach in the treatment of chronic headache: a pilot study. Altern Ther Health Med 2004;10(5):40-46.
[61] Foster NE, Thomas E, Barlas P, Hill JC, Young J, Mason E, Hay EM. Acupuncture as an adjunct to exercise based physiotherapy for osteoarthritis of the knee: randomised controlled trial. BMJ 2007;335(7617):436.
[62] Frega A, Stentella P, Di Renzi F, Gallo G, Palazzetti PL, Del Vescovo M, Ciccarone M, Pachi A. Pain evaluation during carbon dioxide laser vaporization for cervical intraepithelial neoplasia: a randomized trial. Clin Exp Obstet Gynecol 1994;21(3):188-191.
[63] Gale GD, Rothbart PJ, Li Y. Infrared therapy for chronic low back pain: a randomized, controlled trial. Pain Res Manag 2006;11(3):193-196.
[64] Geers A, Helfer S, Weiland P, Kosbab K. Expectations and Placebo Response: A Laboratory Investigation into the Role of Somatic Focus. J Behav Med 2006;29(2):171-178.
[65] Geers AL, Wellman JA, Fowler SL, Helfer SG, France CR. Dispositional optimism predicts placebo analgesia. J Pain 2010;11(11):1165-1171.
[66] Goffaux P, Redmond WJ, Rainville P, Marchand S. Descending analgesia--when the spine echoes what the brain expects. Pain 2007;130(1-2):137-143.
[67] Goodenough B, Kampel L, Champion GD, Laubreaux L, Nicholas MK, Ziegler JB, McInerney M. An investigation of the placebo effect and age-related factors in the report of needle pain from venipuncture in children. Pain 1997;72(3):383-391.
[68] Goodkin K, Gullion CM, Agras WS. A randomized, double-blind, placebo-controlled trial of trazodone hydrochloride in chronic low back pain syndrome. J Clin Psychopharmacol 1990;10(4):269-278.
[69] Grevert P, Albert LH, Goldstein A. Partial antagonism of placebo analgesia by naloxone. Pain 1983;16(2):129-143.
[70] Haake M, Muller HH, Schade-Brittinger C, Basler HD, Schafer H, Maier C, Endres HG, Trampisch HJ, Molsberger A. German acupuncture trials (GERAC) for chronic low back pain. Randomized, multicenter, blinded, parallel-group trial with 3 groups Arch Intern Med 2007;167(17):1892-1898.
[71] Hale ME, Ahdieh H, Ma T, Rauck R. Efficacy and safety of OPANA ER (oxymorphone extended release) for relief of moderate to severe chronic low back pain in opioid-experienced patients: a 12-week, randomized, double-blind, placebo-controlled study. The journal of pain : official journal of the American Pain Society 2007;8(2):175-184.
[72] Hargreaves A, Lander J. Use of Transcutaneous Electrical Nerve Stimulation For Postoperative Pain. Nurs Res 1989;38(3):159-160.
[73] Hargreaves KM, Dionne RA, Mueller GP. Plasma Beta-Endorphin-like Immunoreactivity, Pain and Anxiety Following Administration of Placebo in Oral Surgery Patients. J Dent Res 1983;62(11):1170-1173.
[74] Hashish I, Hai HK, Harvey W, Feinmann C, Harris M. Reduction of postoperative pain and swelling by ultrasound treatment: a placebo effect. Pain 1988;33(3):303-311.

For peer review only

[75] Hashish I, Harvey W, Harris M. Anti-inflammatory effects of ultrasound therapy: evidence for a major placebo effect. Rheumatology (Oxford) 1986;25(1):77-81.

[76] Helms JM. Acupuncture for the management of primary dysmenorrhea. Obstet Gynecol 1987;69(1):51-56.

[77] Hoirus KT, Pfleger B, McDuffie FC, Cotsonis G, Elsangak O, Hinson R, Verzosa GT. A randomized clinical trial comparing chiropractic adjustments to muscle relaxants for subacute low back pain. J Manipulative Physiol Ther 2004;27(6):388-398.

[78] Hong C-Z, Chen Y-C, Pon CH, Yu J. Immediate Effects of Various Physical Medicine Modalities on Pain Threshold of an Active Myofascial Trigger Point. Journal of Musculoskeletal Pain 1993;1(2):37-53.

[79] Hruby G, Ames C, Chen C, Yan Y, Sagar J, Baron P, Landman J. Assessment of efficacy of transcutaneous electrical nerve stimulation for pain management during office-based flexible cystoscopy. Urology 2006;67(5):914-917.

[80] Hyland MR, Webber-Gaffney A, Cohen L, Lichtman SW. Randomized Controlled Trial of Calcaneal Taping, Sham Taping, and Plantar Fascia Stretching for the Short-Term Management of Plantar Heel Pain. J Orthop Sports Phys Ther 2006;36(6):364-371.

[81] Johansen O, Brox J, Flaten MA. Placebo and Nocebo Responses, Cortisol, and Circulating Beta-Endorphin. Psychosom Med 2003;65(5):786-790.

[82] Kaptchuk TJ, Stason WB, Davis RB, Legedza ATR, Schnyer RN, Kerr CE, Stone DA, Nam BH, Kirsch I, Goldman RH. Sham device v inert pill: randomised controlled trial of two placebo treatments. Br Med J 2006;332(7538):391-394.

[83] Katz J, Pennella-Vaughan J, Hetzel RD, Kanazi GE, Dworkin RH. A Randomized, Placebo-Controlled Trial of Bupropion Sustained Release in Chronic Low Back Pain. J Pain 2005;6(10):656-661.

[84] Katz N, Ju WD, Krupa DA, Sperling RS, Bozalis Rodgers D, Gertz BJ, Gimbel J, Coleman S, Fisher C, Nabizadeh S, Borenstein D. Efficacy and safety of rofecoxib in patients with chronic low back pain: results from two 4-week, randomized, placebo-controlled, parallel-group, double-blind trials. Spine (Phila Pa 1976) 2003;28(9):851-858; discussion 859.

[85] Katz N, Rauck R, Ahdieh H, Ma T, Gerritsen van der Hoop R, Kerwin R, Podolsky G. A 12-week, randomized, placebo-controlled trial assessing the safety and efficacy of oxymorphone extended release for opioid-naive patients with chronic low back pain. Curr Med Res Opin 2007;23(1):117-128.

[86] Keltner JR, Furst A, Fan C, Redfern R, Inglis B, Fields HL. Isolating the Modulatory Effect of Expectation on Pain Transmission: A Functional Magnetic Resonance Imaging Study. The Journal of Neuroscience 2006;26(16):4437-4443.

[87] Kerr AR, Drexel CA, Spielman AI. The efficacy and safety of 50 mg penicillin G potassium troches for recurrent aphthous ulcers. Oral Surg Oral Med Oral Pathol Oral Radiol Endod 2003;96(6):685-694.

[88] Ketenci A, Ozcan E, Karamursel S. Assessment of efficacy and psychomotor performances of thiocolchicoside and tizanidine in patients with acute low back pain. Int J Clin Pract 2005;59(7):764-770.

[89] Klaber Moffett JA, Richardson PH, Frost H, Osborn A. A placebo controlled double blind trial to evaluate the effectiveness of pulsed short wave therapy for osteoarthritic hip and knee pain. Pain 1996;67(1):121-127.

[90] Klein RG, Eek BC. Low-energy laser treatment and exercise for chronic low back pain: double-blind controlled trial. Arch Phys Med Rehabil 1990;71(1):34-37.

[91] Kober A, Scheck T, Greher M, Lieba F, Fleischhackl R, Fleischhackl S, Randunsky F, Hoerauf K. Prehospital Analgesia with Acupressure in Victims of Minor Trauma: A Prospective, Randomized, Double-Blinded Trial. Anesth Analg 2002;95(3):723-727 710.1213/00000539-200209000-200200035.

For peer review only

[92] Kong J, Gollub RL, Polich G, Kirsch I, LaViolette P, Vangel M, Rosen B, Kaptchuk TJ. A Functional Magnetic Resonance Imaging Study on the Neural Mechanisms of Hyperalgesic Nocebo Effect. The Journal of Neuroscience 2008;28(49):13354-13362.
[93] Kong J, Gollub RL, Rosman IS, Webb JM, Vangel MG, Kirsch I, Kaptchuk TJ. Brain activity associated with expectancy-enhanced placebo analgesia as measured by functional magnetic resonance imaging. J Neurosci 2006;26(2):381-388.
[94] Kotani N, Kushikata T, Suzuki A, Hashimoto H, Muraoka M, Matsuki A. Insertion of intradermal needles into painful points provides analgesia for intractable abdominal scar pain. Reg Anesth Pain Med 2001;26(6):532-538.
[95] Kupers R, Maeyaert J, Boly M, Faymonville ME, Laureys S. Naloxone-insensitive epidural placebo analgesia in a chronic pain patient. Anesthesiology 2007;106(6):1239-1242.
[96] Lander J, Fowler-Kerry S. TENS for children's procedural pain. Pain 1993;52(2):209-216.
[97] Leibing E, Leonhardt U, Koster G, Goerlitz A, Rosenfeldt J, Hilgers R, Ramadori G. Acupuncture treatment of chronic low-back pain - a randomized, blinded, placebo-controlled trial with 9-month follow-up. Pain 2002;96 189-196.
[98] Levine JD, Gordon NC. Influence of the method of drug administration on analgesic response. Nature 1984;312(5996):755-756.
[99] Licciardone JC, Stoll ST, Fulda KG, Russo DP, Siu J, Winn W, Swift JJ. Osteopathic Manipulative Treatment for Chronic Low Back Pain: A Randomized Controlled Trial. Spine 2003;28(13):1355-1362.
[100] Lieberman MD, Jarcho JM, Berman S, Naliboff BD, Suyenobu BY, Mandelkern M, Mayer EA. The neural correlates of placebo effects: a disruption account. Neuroimage 2004;22(1):447-455.
[101] Limoges MF, Rickabaugh B. Evaluation of TENS During Screening Flexible Sigmoidoscopy. Gastroenterol Nurs 2004;27(2):61-68.
[102] Lin J-G, Lo M-W, Wen Y-R, Hsieh C-L, Tsai S-K, Sun W-Z. The effect of high and low frequency electroacupuncture in pain after lower abdominal surgery. Pain 2002;99(3):509-514.
[103] Linde K, Streng A, Jurgens S, Hoppe A, Brinkhaus B, Witt C, Wagenpfeil S, Pfaffenrath V, Hammes MG, Weidenhammer W, Willich SN, Melchart D. Acupuncture for patients with migraine. A randomized controlled trial. Journal of the American Medical Association 2005;293 (2118):2125.
[104] Liossi C, Hatira P. Clinical Hypnosis in the Alleviation of Procedure-Related Pain in Pediatric Oncology Patients. Int J Clin Exp Hypn 2003;51(1):4-28.
[105] Lipman JJ, Miller BE, Mays KS, Miller MN, North WC, Byrne WL. Peak B endorphin concentration in cerebrospinal fluid: reduced in chronic pain patients and increased during the placebo response. Psychopharmacology (Berl) 1990;102(1):112-116.
[106] Manchikanti L, Pampati V, Damron K. The role of placebo and nocebo effects of perioperative administration of sedatives and opioids in interventional pain management. Pain Physician 2005;8(4):349-355.
[107] Martikainen IK, Hagelberg N, Mansikka H, Hietala J, Någren K, Scheinin H, Pertovaara A. Association of striatal dopamine D2/D3 receptor binding potential with pain but not tactile sensitivity or placebo analgesia. Neurosci Lett 2005;376(3):149-153.
[108] Matre D, Casey KL, Knardahl S. Placebo-Induced Changes in Spinal Cord Pain Processing. The Journal of Neuroscience 2006;26(2):559-563.
[109] Mayberg HS, Silva JA, Brannan SK, Tekell JL, Mahurin RK, McGinnis S, Jerabek PA. The functional neuroanatomy of the placebo effect. A J Psychiatry 2002;159(5):728-737.
[110] Melchart D, Streng A, Hoppe A, Brinkhaus B, Witt C, Wagenpfeil S, Pfaffenrath V, Hammes M, Hummelsberger J, Irnich D, Weidenhammer W, Willich SN, Linde K. Acupuncture in patients with tension-type headache: randomised controlled trial. Br Med J 2005;331 376-382.
[111] Molsberger AF, Mau J, Pawelec DB, Winkler J. Does acupuncture improve the orthopedic management of chronic low back pain – a randomized, blinded, controlled trial with 3 months follow up. Pain 2002;99(3):579-587.

For peer review only

[112] Montgomery G, Kirsch I. Mechanisms of Placebo Pain Reduction: An Empirical Investigation. Psychological Science 1996;7(3):174-176.
[113] Montgomery GH, Kirsch I. Classical conditioning and the placebo effect. Pain 1997;72(1-2):107-113.
[114] Morton DL, Watson A, El-Deredy W, Jones AKP. Reproducibility of placebo analgesia: Effect of dispositional optimism. Pain 2009;146(1â€"2):194-198.
[115] Muehlbacher M, Nickel MK, Kettler C, Tritt K, Lahmann C, Leiberich PK, Nickel C, Krawczyk J, Mitterlehner FO, Rother WK, Loew TH, Kaplan P. Topiramate in treatment of patients with chronic low back pain: a randomized, double-blind, placebo-controlled study. Clin J Pain 2006;22(6):526-531.
[116] Nemoto H, Nemoto Y, Toda H, Mikuni M, Fukuyama H. Placebo analgesia: a PET study. Exp Brain Res 2007;179(4):655-664.
[117] Ockene JK, Barad DH, Cochrane BB, Larson JC, Gass M, Wassertheil-Smoller S, Manson JE, Barnabei VM, Lane DS, Brzyski RG, Rosal MC, Wylie-Rosett J, Hays J. Symptom experience after discontinuing use of estrogen plus progestin. JAMA 2005;294(2):183-193.
[118] Pallay RM, Seger W, Adler JL, Ettlinger RE, Quaidoo EA, Lipetz R, O'Brien K, Mucciola L, Skalky CS, Petruschke RA, Bohidar NR, Geba GP. Etoricoxib reduced pain and disability and improved quality of life in patients with chronic low back pain: a 3 month, randomized, controlled trial. Scand J Rheumatol 2004;33(4):257-266.
[119] Pariente J, White P, Frackowiak RSJ, Lewith G. Expectancy and belief modulate the neuronal substrates of pain treated by acupuncture. Neuroimage 2005;25(4):1161-1167.
[120] Peloso PM, Fortin L, Beaulieu A, Kamin M, Rosenthal N. Analgesic efficacy and safety of tramadol/ acetaminophen combination tablets (Ultracet) in treatment of chronic low back pain: a multicenter, outpatient, randomized, double blind, placebo controlled trial. J Rheumatol 2004;31(12):2454-2463.
[121] Petrovic P, Kalso E, Petersson KM, Ingvar M. Placebo and opioid analgesia-- imaging a shared neuronal network. Science 2002;295(5560):1737-1740.
[122] Pollo A, Amanzio M, Arslanian A, Casadio C, Maggi G, Benedetti F. Response expectancies in placebo analgesia and their clinical relevance. Pain 2001;93(1):77-84.
[123] Pollo A, Vighetti S, Rainero I, Benedetti F. Placebo analgesia and the heart. Pain 2003;102(1-2):125-133.
[124] Preyde M. Effectiveness of massage therapy for subacute low-back pain: a randomized controlled trial. Can Med Assoc J 2000;162(13):1815-1820.
[125] Price DD, Craggs J, Nicholas Verne G, Perlstein WM, Robinson ME. Placebo analgesia is accompanied by large reductions in pain-related brain activity in irritable bowel syndrome patients. Pain 2007;127(1):63-72.
[126] Price DD, Long S, Wilsey B, Rafii A. Analysis of peak magnitude and duration of analgesia produced by local anesthetics injected into sympathetic ganglia of complex regional pain syndrome patients. Clin J Pain 1998;14(3):216-226.
[127] Price DD, Milling LS, Kirsch I, Duff A, Montgomery GH, Nicholls SS. An analysis of factors that contribute to the magnitude of placebo analgesia in an experimental paradigm. Pain 1999;83(2):147-156.
[128] Rainville P, Duncan GH, Price DD, Carrier B, Bushnell MC. Pain affect encoded in human anterior cingulate but not somatosensory cortex. Science 1997;277(5328):968-971.
[129] Rawling MJ, Wiebe ER. A randomized controlled trial of fentanyl for abortion pain. Am J Obstet Gynecol 2001;185(1):103-107.
[130] Ristikankare M, Hartikainen J, Heikkinen M, Janatuinen E, Julkunen R. Is routinely given conscious sedation of benefit during colonoscopy? Gastrointest Endosc 1999;49(5):566-572.
[131] Robinson R, Darlow S, Wright SJ, Watters C, Carr I, Gadsby G, Mayberry J. Is transcutaneous electrical nerve stimulation an effective analgesia during colonoscopy? Postgrad Med J 2001;77(909):445-446.

For peer review only

[132] Rowbotham MC, Davies PS, Verkempinck C, Galer BS. Lidocaine patch: double-blind controlled study of a new treatment method for post-herpetic neuralgia. Pain 1996;65(1):39-44.
[133] Ruoff GE, Rosenthal N, Jordan D, Karim R, Kamin M. Tramadol/Acetaminophen combination tablets for the treatment of chronic lower back pain: A multicenter, randomized, double-blind, placebo-controlled outpatient study. Clin Ther 2003;25(4):1123-1141.
[134] Sanders GE, Reinert O, Tepe R, Maloney P. Chiropractic adjustive manipulation on subjects with acute low back pain: visual analog pain scores and plasma beta-endorphin levels. J Manipulative Physiol Ther 1990;13(7):391-395.
[135] Scharf HP, Mansmann U, Streitberger K, Witte S, Kramer J, Maier C, Trampisch HJ, Victor N. Acupuncture and knee osteoarthritis: A three-armed randomized trial. Ann Intern Med 2006;145(1):12-20.
[136] Scharff L, Marcus DA, Masek BJ. A Controlled Study of Minimal-Contact Thermal Biofeedback Treatment in Children With Migraine. J Pediatr Psychol 2002;27(2):109-119.
[137] Schnebel BE, Simmons JW. The use of oral colchicine for low-back pain. A double-blind study. Spine (Phila Pa 1976) 1988;13(3):354-357.
[138] Schnitzer TJ, Gray WL, Paster RZ, Kamin M. Efficacy of tramadol in treatment of chronic low back pain. J Rheumatol 2000;27(3):772-778.
[139] Scott DJ, Stohler CS, Egnatuk CM, Wang H, Koeppe RA, Zubieta J-K. Individual Differences in Reward Responding Explain Placebo-Induced Expectations and Effects. Neuron 2007;55(2):325-336.
[140] Scott DJ, Stohler CS, Egnatuk CM, Wang H, Koeppe RA, Zubieta J. PLacebo and nocebo effects are defined by opposite opioid and dopaminergic responses. Arch Gen Psychiatry 2008;65(2):220-231.
[141] Snyder-Mackler L, Barry AJ, Perkins AI, Soucek MD. Effects of helium-neon laser irradiation on skin resistance and pain in patients with trigger points in the neck or back. Phys Ther 1989;69(5):336-341.
[142] Soriano F, Rios R. Gallium arsenide laser treatment of chronic low back pain: A prospective, randomized and double blind study. Laser Therapy 1998;10(4):175-180.
[143] Stransky M, Rubin A, Lava NS, Lazaro RP. Treatment of carpal tunnel syndrome with vitamin B6: a double-blind study. South Med J 1989;82(7):841-842.
[144] Szpalski M, Hayez JP. Objective functional assessment of the efficacy of tenoxicam in the treatment of acute low back pain. A double-blind placebo-controlled study. Br J Rheumatol 1994;33(1):74-78.
[145] Tashjian RZ, Banerjee R, Bradley MP, Alford W, Fadale PD. Zolpidem reduces postoperative pain, fatigue, and narcotic consumption following knee arthroscopy: a prospective randomized placebo-controlled double-blinded study. J Knee Surg 2006;19(2):105-111.
[146] Theroux MC, West DW, Corddry DH, Hyde PM, Bachrach SJ, Cronan KM, Kettrick RG. Efficacy of intranasal midazolam in facilitating suturing of lacerations in preschool children in the emergency department. Pediatrics 1993;91(3):624-627.
[147] Thomas KS, Muir KR, Doherty M, Jones AC, O'Reilly SC, Bassey EJ. Home based exercise programme for knee pain and knee osteoarthritis: randomised controlled trial. BMJ 2002;325(7367):752.
[148] Toya S, Motegi M, Inomata K, Ohshiro T, Macda T. Report on a computer-randomized double blind clinical trial to determine the effectiveness of the GaAlAs (830 nm) diode laser for pain attenuation in selected pain groups. Laser Therapy 1994;6:143-148.
[149] Tritrakarn T, Lertakyamanee J, Koompong P, Soontrapa S, Somprakit P, Tantiwong A, Jittapapai S. Both EMLA and Placebo Cream Reduced Pain during Extracorporeal Piezoelectric Shock Wave Lithotripsy with the Piezolith 2300. Anesthesiology 2000;92(4):1049-1054.
[150] Vase L, Robinson ME, Verne GN, Price DD. The contributions of suggestion, desire, and expectation to placebo effects in irritable bowel syndrome patients: An empirical investigation. Pain 2003;105(1-2):17-25.

For peer review only

[151] Vase L, Robinson ME, Verne GN, Price DD. Increased placebo analgesia over time in irritable bowel syndrome (IBS) patients is associated with desire and expectation but not endogenous opioid mechanisms. Pain 2005;115(3):338-347.

[152] Verne GN, Robinson ME, Vase L, Price DD. Reversal of visceral and cutaneous hyperalgesia by local rectal anesthesia in irritable bowel syndrome (IBS) patients. Pain 2003;105(1):223-230.

[153] Vondrackova D, Leyendecker P, Meissner W, Hopp M, Szombati I, Hermanns K, Ruckes C, Weber S, Grothe B, Fleischer W, Reimer K. Analgesic efficacy and safety of oxycodone in combination with naloxone as prolonged release tablets in patients with moderate to severe chronic pain. The journal of pain : official journal of the American Pain Society 2008;9(12):1144-1154.

[154] Vorsanger GJ, Xiang J, Gana TJ, Pascual ML, Fleming RR. Extended-release tramadol (tramadol ER) in the treatment of chronic low back pain. J Opioid Manag 2008;4(2):87-97.

[155] Voudouris NJ, Peck CL, Coleman G. Conditioned placebo responses. J Pers Soc Psychol 1985;48(1):47-53.

[156] Voudouris NJ, Peck CL, Coleman G. Conditioned response models of placebo phenomena: further support. Pain 1989;38(1):109-116.

[157] Voudouris NJ, Peck CL, Coleman G. The role of conditioning and verbal expectancy in the placebo response. Pain 1990;43(1):121-128.

[158] Wager TD, Matre D, Casey KL. Placebo effects in laser-evoked pain potentials. Brain Behav Immun 2006;20(3):219-230.

[159] Wager TD, Rilling JK, Smith EE, Sokolik A, Casey KL, Davidson RJ, Kosslyn SM, Rose RM, Cohen JD. Placebo-Induced Changes in fMRI in the Anticipation and Experience of Pain. Science 2004;303(5661):1162-1167.

[160] Wager TD, Scott DJ, Zubieta J-K. Placebo effects on human μ-opioid activity during pain. Proceedings of the National Academy of Sciences 2007;104(26):11056-11061.

[161] Walton RE, Chiappinelli J. Prophylactic penicillin: effect on posttreatment symptoms following root canal treatment of asymptomatic periapical pathosis. J Endod 1993;19(9):466-470.

[162] Wang B, Tang J, White PF, Naruse R, Sloninsky A, Kariger R, Gold J, Wender RH. Effect of the Intensity of Transcutaneous Acupoint Electrical Stimulation on the Postoperative Analgesic Requirement. Anesth Analg 1997;85(2):406-413.

[163] Watson A, El-Deredy W, Bentley DE, Vogt BA, Jones AKP. Categories of placebo response in the absence of site-specific expectation of analgesia. Pain 2006;126(1):115-122.

[164] Watson A, El-Deredy W, Vogt BA, Jones AKP. Placebo analgesia is not due to compliance or habituation: EEG and behavioural evidence. Neuroreport 2007;18(8):771-775 710.1097/WNR.1090b1013e3280c1091e1092a1098.

[165] Webster LR, Butera PG, Moran LV, Wu N, Burns LH, Friedmann N. Oxytrex minimizes physical dependence while providing effective analgesia: a randomized controlled trial in low back pain. The journal of pain : official journal of the American Pain Society 2006;7(12):937-946.

[166] Witt C, Brinkhaus B, Jena S, Linde K, Streng A, Wagenpfeil S, Hummelsberger J, Walther HU, Melchart D, Willich SN. Acupuncture in patients with osteoarthritis of the knee: a randomised trial. Lancet 2005;366 136-143.

[167] Wu M-T, Sheen J-M, Chuang K-H, Yang P, Chin S-L, Tsai C-Y, Chen C-J, Liao J-R, Lai P-H, Chu K-A, Pan H-B, Yang C-F. Neuronal Specificity of Acupuncture Response: A fMRI Study with Electroacupuncture. Neuroimage 2002;16(4):1028-1037.

[168] Zubieta JK, Bueller JA, Jackson LR, Scott DJ, Xu Y, Koeppe RA, Nichols TE, Stohler CS. Placebo Effects Mediated by Endogenous Opioid Activity on æ-Opioid Receptors. The Journal of Neuroscience 2005;25(34):7754-7762.

[169] Zubieta JK, Yau WY, Scott DJ, Stohler CS. Belief or Need? Accounting for individual variations in the neurochemistry of the placebo effect. Brain Behav Immun 2006;20(1):15-26.

For peer review only

PRISMA 2009 Checklist

Section/topic	#	Checklist item	Reported on page #
**TITLE**			
Title	1	Identify the report as a systematic review, meta-analysis, or both.	n/a
**ABSTRACT**			
Structured summary	2	Provide a structured summary including, as applicable: background; objectives; data sources; study eligibility criteria, participants, and interventions; study appraisal and synthesis methods; results; limitations; conclusions and implications of key findings; systematic review registration number.	n/a
**INTRODUCTION**			
Rationale	3	Describe the rationale for the review in the context of what is already known.	3
Objectives	4	Provide an explicit statement of questions being addressed with reference to participants, interventions, comparisons, outcomes, and study design (PICOS).	n/a
**METHODS**			
Protocol and registration	5	Indicate if a review protocol exists, if and where it can be accessed (e.g., Web address), and, if available, provide registration information including registration number.	n/a
Eligibility criteria	6	Specify study characteristics (e.g., PICOS, length of follow-up) and report characteristics (e.g., years considered, language, publication status) used as criteria for eligibility, giving rationale.	5
Information sources	7	Describe all information sources (e.g., databases with dates of coverage, contact with study authors to identify additional studies) in the search and date last searched.	4
Search	8	Present full electronic search strategy for at least one database, including any limits used, such that it could be repeated.	n/a
Study selection	9	State the process for selecting studies (i.e., screening, eligibility, included in systematic review, and, if applicable, included in the meta-analysis).	4, Fig1
Data collection process	10	Describe method of data extraction from reports (e.g., piloted forms, independently, in duplicate) and any processes for obtaining and confirming data from investigators.	6
Data items	11	List and define all variables for which data were sought (e.g., PICOS, funding sources) and any assumptions and simplifications made.	6
Risk of bias in individual studies	12	Describe methods used for assessing risk of bias of individual studies (including specification of whether this was done at the study or outcome level), and how this information is to be used in any data synthesis.	n/a
Summary measures	13	State the principal summary measures (e.g., risk ratio, difference in means).	n/a
Synthesis of results	14	Describe the methods of handling data and combining results of studies, if done, including measures of consistency (e.g., I^2) for each meta-analysis.	6

For peer review only

PRISMA 2009 Checklist

Section/topic	#	Checklist item	Reported on page #
Risk of bias across studies	15	Specify any assessment of risk of bias that may affect the cumulative evidence (e.g., publication bias, selective reporting within studies).	n/a
Additional analyses	16	Describe methods of additional analyses (e.g., sensitivity or subgroup analyses, meta-regression), if done, indicating which were pre-specified.	n/a
**RESULTS**			
Study selection	17	Give numbers of studies screened, assessed for eligibility, and included in the review, with reasons for exclusions at each stage, ideally with a flow diagram.	Fig1
Study characteristics	18	For each study, present characteristics for which data were extracted (e.g., study size, PICOS, follow-up period) and provide the citations.	n/a
Risk of bias within studies	19	Present data on risk of bias of each study and, if available, any outcome level assessment (see item 12).	n/a
Results of individual studies	20	For all outcomes considered (benefits or harms), present, for each study: (a) simple summary data for each intervention group (b) effect estimates and confidence intervals, ideally with a forest plot.	n/a
Synthesis of results	21	Present results of each meta-analysis done, including confidence intervals and measures of consistency.	n/a
Risk of bias across studies	22	Present results of any assessment of risk of bias across studies (see Item 15).	n/a
Additional analysis	23	Give results of additional analyses, if done (e.g., sensitivity or subgroup analyses, meta-regression [see Item 16]).	n/a
**DISCUSSION**			
Summary of evidence	24	Summarize the main findings including the strength of evidence for each main outcome; consider their relevance to key groups (e.g., healthcare providers, users, and policy makers).	21
Limitations	25	Discuss limitations at study and outcome level (e.g., risk of bias), and at review-level (e.g., incomplete retrieval of identified research, reporting bias).	22-3
Conclusions	26	Provide a general interpretation of the results in the context of other evidence, and implications for future research.	22-3
**FUNDING**			
Funding	27	Describe sources of funding for the systematic review and other support (e.g., supply of data); role of funders for the systematic review.	1

From: Moher D, Liberati A, Tetzlaff J, Altman DG, The PRISMA Group (2009). Preferred Reporting Items for Systematic Reviews and Meta-Analyses: The PRISMA Statement. PLoS Med 6(7): e1000097. doi:10.1371/journal.pmed1000097

For more information, visit: **www.prisma-statement.org**.

For peer review only - http://bmjopen.bmj.com/site/about/guidelines.xhtml
